# NMR Analysis and Assignment of a Biosynthesis Gene Cluster for Trichokonins VI and VIII, Antiplasmodial Large Peptaibols Produced by a *Trichoderma* sp. Fungus

**DOI:** 10.1021/acsomega.5c05271

**Published:** 2025-10-10

**Authors:** Ariane F. Bertonha, David E. Williams, Karen J. Nicacio, Marcelo R. de Amorim, Sydney M. Schoellhorn, Anna Caroline C. Aguiar, Talita Alvarenga Valdes, Giovana Rossi Mendes, Igor M. R. Moura, Lamonielli F. Michaliski, Matheus Gotha, Caue A. W. Zuccarino, Lara D. Sette, Antônio G. Ferreira, Raymond J. Andersen, Rafael Victorio Carvalho Guido, Roberto G. S. Berlinck

**Affiliations:** † Instituto de Química de São Carlos, Universidade de São Paulo, São Carlos, SP 13560-970, Brazil; ⊥ Departments of Chemistry and EOAS, 8166 University of British Columbia, 2036 Main Mall, Vancouver, BC V6T 1Z6, Canada; ‡ Brazilian Biosciences National Laboratory (LNBio), Brazilian Center for Research in Energy and Materials (CNPEM), Campinas, SP 13083-970, Brazil; ∧ Universidade Federal de Mato Grosso (UFMT), Cuiabá, MT 78060-900, Brazil; ∇ Department of Chemistry and BioDiscovery Institute, 3404 University of North Texas, 1155 Union Circle, Denton, Texas 76203, United States; O Department of Microbiology, Immunology and Parasitology, Federal University of São Paulo, São Paulo, SP 04023-062, Brazil; § Departamento de Biologia Geral e Aplicada, Universidade Estadual Paulista (UNESP), Instituto de Biociências, Rio Claro, SP 13506-900, Brazil; ∥ Departamento de Química, Universidade Federal de São Carlos, São Carlos, SP 13565-905, Brazil; # São Carlos Institute of Physics, University of São Paulo, São Carlos, SP 13566-590, Brazil

## Abstract

Peptaibols are modified linear peptides that typically include an acyl fragment connected at the *N*-terminal moiety, a reduced acid extremity and α-aminoisobutyric acid residues as common features, as well as other structural modifications. Peptaibols very often display potent biological activity, in particular, antimicrobial activity. Trichokonins VI (**1**) and VIII (**2**) are large peptaibols composed of 20 amino acids, which were previously only characterized by analysis of MS/MS data. We herein report the first full characterization of trichokonins VI (**1**) and VIII (**2**) by analysis of NMR data, along with an analysis by electronic circular dichroism and HRMS/MS data. Also, full genome sequencing and analysis of *Trichoderma* sp. L2-2 allowed us to identify a peptaibol biosynthesis gene cluster and the proposal for a biosynthetic assembly of trichokonins VI and VIII. Trichokonins VI and VIII also demonstrated antiplasmodial activity at the submicromolar range against *Plasmodium falciparum*.

## Graphical abstract

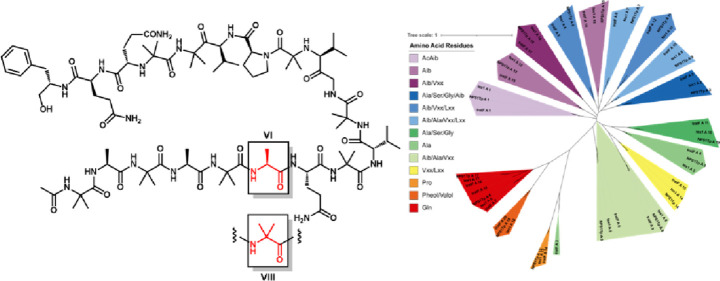

## Introduction

Polar organisms have been of increasing interest in biotechnology and natural products chemistry because of their unique biological traits, including the expression of enzymes and secondary metabolites, resulting from adaptations in an extreme environment.^1,2^ Among the most investigated polar organisms, fungi from Antarctica stand up as producers of a number of unique enzymes and bioactive natural products.^1–4^ New peptaibols of nine amino acid residues isolated from cultures of *Tridocherma asperellum*, new highly oxygenated polyketides produced in culture by *Penicillium crustosum* PRB-2, new epipolythiodioxopiperazine alkaloids produced by *Oidiodendron truncatum* GW3-13, the highly complex heptacyclic polyketide talaverrucin A from cultures of *Talaromyces* sp. HDN151403, and the very uncommon sesquiterpenoids bearing a very rare nitrobenzyl group isolated from cultures of *Aspergillus insulicola* HDN151418 represent some of the various outstanding metabolites produced by fungi from Antarctica.^4^

*Trichoderma*, and its anamorph *Hypocrea*, is a fungal genus found in a wide range of habitats. It is reported to include more than 200 individual described species.^5,6^ Secondary metabolites produced by *Trichoderma* spp. have mainly been used commercially in biocontrol.^7–9^ Additionally, *Trichoderma* spp. secondary metabolites present cytotoxic, antimicrobial, and antioxidant activities, as well as metabolites enhancing plant growth.^10,11^ Modified peptides known as peptaibols are commonly produced by *Trichoderma* species.^12–15^ Peptaibols comprise a large peptide family, with over 1000 described peptidic entities, which can be linear or cyclic and are constructed from between 7 and 20 amino acid residues, many of which display cytotoxic and antibacterial activity.^16^ Common features of peptaibols include the incorporation of α-aminoisobutyrate (Aib) residues, additional uncommon amino acids, an acylated *N*-terminus, and an amino alcohol of the reduced acid at the *C*-terminus.^16,17^

In our continuing search for bioactive secondary metabolites produced in culture by diverse fungi,^18–22^ we have isolated trichokonins VI (**1**) and VIII (**2**), as well as additional peptaibols (**3**–**6**) from cultures of *Trichoderma* sp. L2-2, a strain obtained from a lichen sample collected at Admiralty Bay in Antarctica. Since no NMR data have been reported for trichokonins VI and VIII, we have performed complete NMR analyses to assign ^1^H and ^13^C, also the electronic circular dichroism (ECD) spectra, for both peptaibols, as well as their antiplasmodial activity. Additionally, we have also performed a bioinformatic analysis of *Trichoderma* sp. L2-2 genome and a putative biosynthetic gene cluster (BGC) related to the biosynthesis of trichokonins VI (**1**) and VIII (**2**) was assigned.

Previous investigations on trichokonins VI (**1**) and VIII (**2**) include isolation and identification by mass spectrometry analysis.^23^ In that investigation, only trichokonin VII was identified by NMR analysis, but not trichokonins VI and VIII.^23^ Trichokonin VI was reported with agonistic activity on L-type Ca^2+^ channels of cardiac membranes.^24^ Optimal conditions for the production of trichokonins VI and VIII have been reported.^25^ The cytotoxicity mechanism of trichokonin VI on hepatocellular carcinoma (HCC) cells has been investigated. Trichokonin VI inhibits the growth of HCC cells in a dose-dependent manner. Apoptosis and autophagy are the likely mechanisms involved in trichokonin VI cytotoxic activity.^26^ Trichokonin VI also induces apoptosis in cells of the plant fungal pathogen *Fusarium oxysporum* ^27^ and presents a growth-inhibitory effect on *Arabidopsis* primary roots by inhibiting cell division and elongation processes.^28^ It was also verified that trichokonin VI increased the auxin content and promoted disturbance in auxin signaling gradients within root tips.^28^ The same peptaibol promotes the growth and induces a systemic resistance against the gray mold disease caused by the fungus *Botrytis cinerea* in *Phalaenopsis* orchids.^29^ Also, both peptaibols **1** and **2** displayed genotoxic activity on ovary CHO-K1 cancer cells.^30^ In the case of trichokonin VIII (**2**), its ^1^H and ^13^C NMR spectra were recently reported, but the data were not assigned, although its extensive MS/MS analysis was presented.^31^

## Results and Discussion

### Isolation and Complete Characterization of Trichokonins VI and VIII

The EtOAc-soluble extract of cultures of *Trichoderma* sp. L2-2 was fractionated on a silica-gel-bonded cyanopropyl column to give six fractions. Fraction AT1M5 indicated compounds with a molecular weight higher than 500 Da by HPLC-MS analyses. This fraction was subjected to a separation and purification procedure (see the Experimental Section) that led to the isolation of trichokonin VI (**1**) and trichokonin VIII (**2**). The peptaibols trichogin A IV (**3**),^32,33^ hypocrin NPDG F (**4**),^34^ hypocrin NPDG H (**5**),^35^ and trikoningin KB I (**6**)^35,36^ were also isolated and identified by analysis of spectroscopic and spectrometric data and by comparison with literature data.

Trichokonins VI and VIII were isolated as white, amorphous powders. Their HRMS-(+) spectra exhibited pseudomolecular ions at *m*/*z* 774.4507 for **1** and *m*/*z* 774.4497 for **2** (Figures 1 and 2). This ion was likewise observed in the mass spectra of various peptaibol compounds produced by members of the *Longibrachiatum* clade of the filamentous fungus genus *Trichoderma*, such as alamethicin F50, trilongins BI and BIII, some trichoaureocins, suzukacillins, longibrachins, trichokonins, paracelsins, and others.^37,38^ The mass spectra of 20-residue peptaibols often display characteristic features, including the fragmentation of the secondary amide bond between Aib^13^-Pro^14^.^39^ Proline residues are often found in long-chain peptaibols (7% of the composition), usually in the middle of the amino acid chain.^17^ MS cleavage at the Pro residue leads to the formation of “acylium ion”-type fragments, which are usually considered as diagnostic peaks for the characterization of long-chain peptaibols. The MS values at *m*/*z* 774.4507 for **1** and *m*/*z* 774.4497 for **2** correspond to the *C*-terminal sequence Pro-Val-Aib-Aib-Gln-Gln-Pheol. To determine the complete sequence of the two complementary charged fragments, b_13_ and y_7_, fragmentation of the complementary pseudomolecular ions of the isolated peptides was performed. Fragmentation of the pseudomolecular ions at *m*/*z* 1163.6771 for **1** (Figure S8) indicated the AA sequence Ac-Aib-Ala-Aib-Ala-Aib-Ala-Gln-Aib-Val-Aib-Gly-Leu-Aib, known as trichokonin VI. MS fragmentation of the pseudomolecular ion at *m*/*z* 1177.6910 of compound **2** indicated the sequence Ac-Aib-Ala-Aib-Ala-Aib-Aib-Gln-Aib-Val-Aib-Gly-Leu-Aib, corresponding to trichokonin VIII (Figure S21).

**Figure 1:**
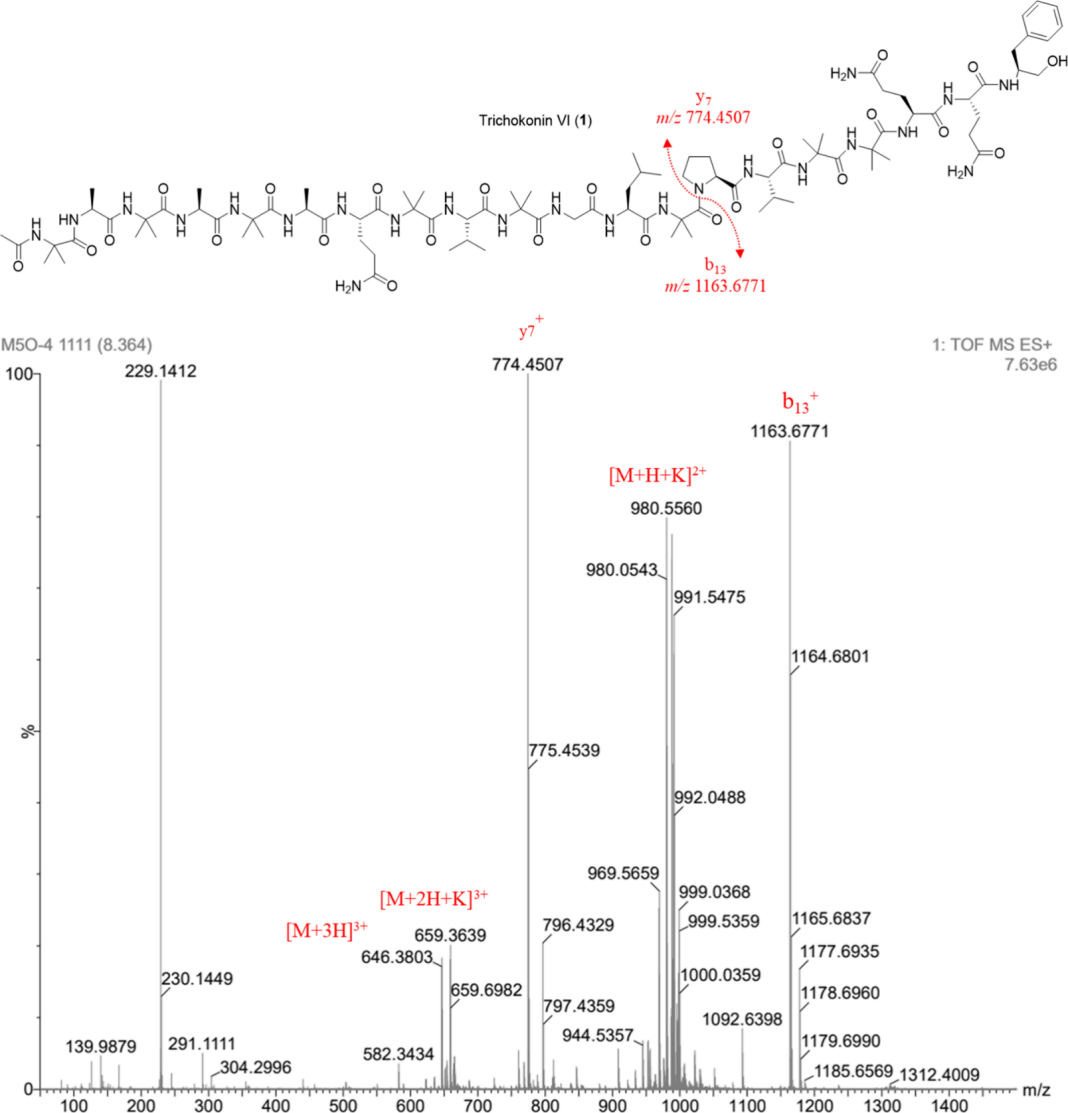
(+)-HRESIMS spectrum of trichokonin VI (**1**) indicating key fragments b_13_ and y_7_.

**Figure 2:**
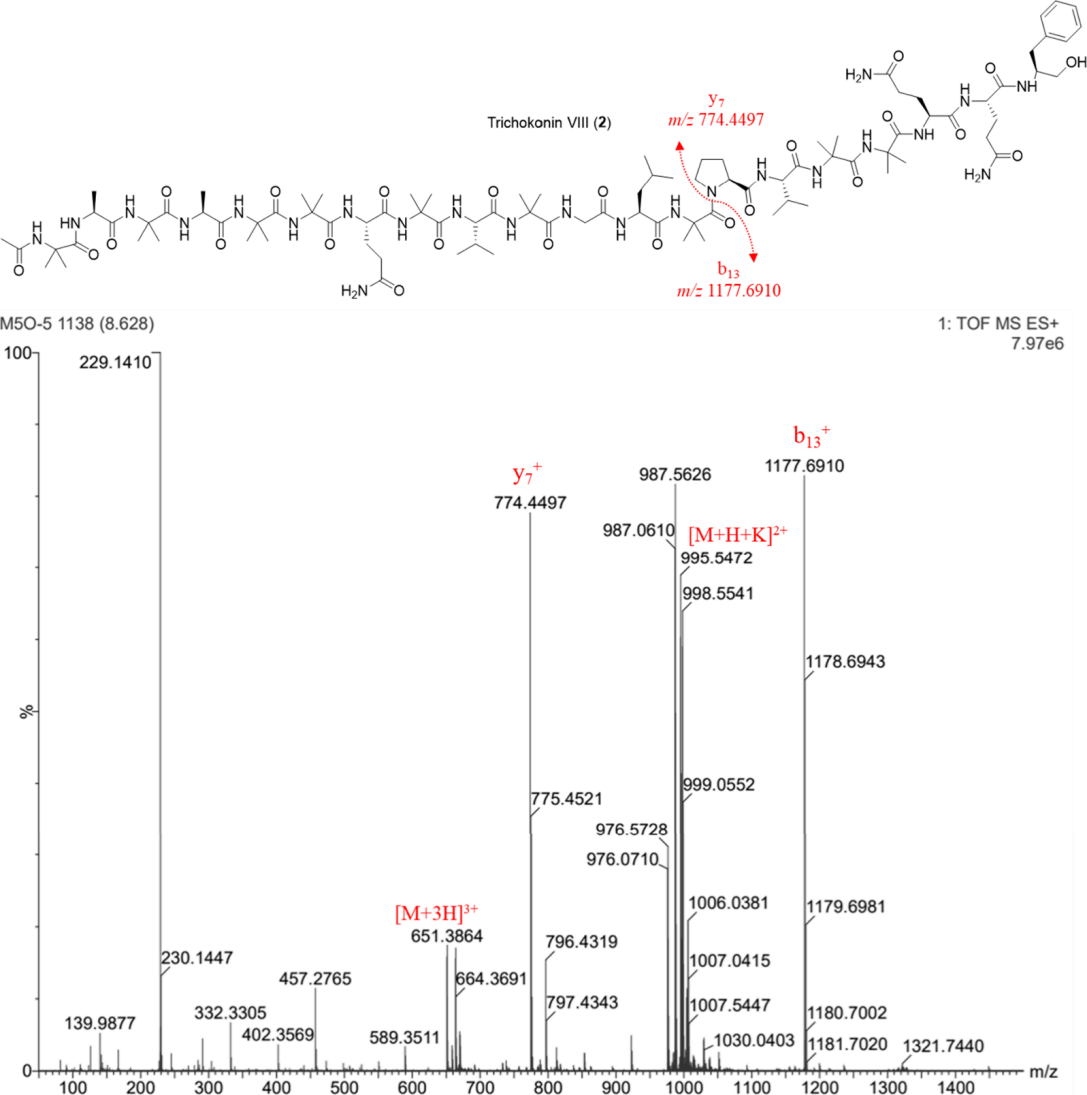
(+)-HRESIMS spectrum of trichokonin VIII (**2**) indicating key fragments b_13_ and y_7_.

The specific rotation measured for trichokonin VI (**1**) was [α]_D_^21^ = −9.0 (*c* 0.02, MeOH), while the literature-reported value was [α]_D_^20^ = −10.0 (*c* 0.2, MeOH).^40^ As for trichokonin VIII (**2**), the measured specific rotation was [α]_D_^21^ = −23.0 (*c* 0.02, MeOH), with no data found in the literature. Marfey’s analysis of both peptaibols **1** and **2** indicated the absolute stereochemistry of all proteinogenic amino acids as L (*S*) (see the Supporting Information).

Analysis of the NMR data of trichokonins VI and VIII enabled us to completely assign the ^1^H and ^13^C signals of both peptides for the first time (Tables 1 and 2).

**Table 1:** ^1^H (600 MHz) and ^13^C (150 MHz) NMR Data for Trichokonin VI (1) (DMSO-*d*_6_)

	position	δ_C_	δ_H_ (*J* in Hz)		position	δ_C_	δ_H_ (*J* in Hz)		position	δ_C_	δ_H_ (*J* in Hz)		position	δ_C_	δ_H_ (*J* in Hz)		position	δ_C_	δ_H_ (*J* in Hz)
Ac	CH_3_	23.1	1.92 s	Gln^7^	NH		7.95	Leu^12^	NH		7.72 d (7.7)	Aib^16^	NH		7.59	Gln^19^	NH		7.50, d (7.7)
	C=O	170.6			α CH	56.3	3.77		α CH	51.5	4.29		α C	55.4			α CH	53.5	3.96
Aib^1^	NH		8.62		β_1_ CH_2_	25.8	1.98		β_1_ CH_2_	39.8	1.41		β_1_ CH_3_	22.6	1.30–1.48		β_1_ CH_2_	26.9	1.78
	α C	55.4			β_2_ CH_2_	25.8	2.13		β_2_ CH_2_	39.8	1.81		β_2_ CH_3_	26.5	1.49		β_2_ CH_2_	26.9	1.88
	β_1_ CH_3_	26.5	1.43		γ_1_ CH_2_	31.0	2.15		γ CH	24.0	1.77		C=O	175.7			δ_1_ CH_2_	31.7	1.98
	β_2_ CH_3_	25.8	1.43		γ_2_ CH_2_	31.0	2.29		δ_1_ CH_3_	20.9	0.82 d (6.5)	Aib^17^	NH		7.86		δ_2_ CH_2_	31.7	2.28
	C=O	176.1			C(O)NH_2_	173.2	6.73 s, 7.15 s		δ_2_ CH_3_	22.9	0.86 d (6.5)		α C	55.8			C(O)NH_2_	173.7	6.73, s; 7.15, s
Ala^2^	NH		8.60		C=O	173.8			C=O	172.9			β_1_ CH_3_	26.3	1.39		C=O	170.9	
	α CH	51.8	3.95	Aib^8^	NH		7.87	Aib^13^	NH		8.17, s		β_2_ CH_3_	22.9	1.47	Pheol^20^	NH		6.95, d (8.8)
	β_1_ CH_3_	16.3	1.32 d (7.2)		α C	55.9			α C	56.1			C=O	175.5			α CH	52.3	3.89
	C=O	174.9			β_1_ CH_3_	22.9	1.36		β_1_ CH_3_	22.6	1.30–1.48	Gln^18^	NH		7.61		β_1_ CH_2_	36.7	2.54, dd (9.0, 13.6)
Aib^3^	NH		7.96		β_2_ CH_3_	26.5	1.42		β_2_ CH_3_	23.7	1.35		α CH	54.7	3.85		β_2_ CH_2_	36.7	2.91, dd (4.7, 13.6)
	α C	55.4			C=O	175.4			C=O	173.5			β_1_ CH_2_	26.5	1.97		C-1	139.1	
	β_1_ CH_3_	25.8	1.30–1.48	Val^9^	NH		7.30 br d (5.4)	Pro^14^	α_1_ CH_2_	48.5	3.55		β_2_ CH_2_	26.5	1.97		C-2, C-6	129.2	7.22
	β_2_ CH_3_	22.9	1.30–1.48		α CH	62.7	3.62 dd (6.5, 8.7)		α_2_ CH_2_	48.5	3.73		δ_1_ CH_2_	31.6	2.10		C-3, C-5	127.9	7.19
	C=O	175.7			β CH	28.7	2.20		β CH_2_	25.5	1.87		δ_2_ CH_2_	31.6	2.30		C-4	125.8	7.13 dt (1.4, 7.2)
Ala^4^	NH		7.88		γ_1_ CH_3_	19.1	0.88 d (6.5)		γ_1_ CH_2_	28.7	1.68		C(O)NH_2_	173.3	6.62, s; 7.07, s		γ_1_ CH_2_	63.0	3.32
	α CH	51.8	3.90		γ_2_ CH_3_	19.6	1.02 d (6.5)		γ_2_ CH_2_	28.7	2.22		C=O	172.0			γ_2_ CH_2_	63.0	3.34
	β_1_ CH_3_	16.5	1.41		C=O	173.0			δ CH	62.6	4.28						OH		4.62^a^
	C=O	175.1		Aib^10^	NH		8.03 s		C=O	173.4									
Aib^5^	NH		7.59 s		α C	55.8		Val^15^	NH		7.59								
	α C	56.0			β_1_ CH_3_	25.8	1.43		α CH	61.1	3.77								
	β_1_ CH_3_	25.7	1.41		β_2_ CH_3_	22.6	1.30–1.48		β CH	28.6	2.25								
	β_2_ CH_3_	23.0	1.48		C=O	175.9			γ_1_ CH_3_	19.0	0.89								
	C=O	172.6		Gly^11^	NH		8.13 t (5.7)		γ_2_ CH_3_	19.1	0.95								
Ala^6^	NH		7.94		α_1_ CH_2_	43.8	3.51		C=O	175.7									
	α CH	52.2	3.89		α_2_ CH_2_	43.8	3.75												
	β_1_ CH_3_	16.2	1.35		C=O	169.6													
	C=O	176.1																	

a Possible OH chemical shift.

**Table 2:** ^1^H (600 MHz) and ^13^C (150 MHz) NMR Data for Trichokonin VIII (2) (DMSO-*d*_6_)

	position	δ_C_	δ_H_ (*J* in Hz)		position	δ_C_	δ_H_ (*J* in Hz)		position	δ_C_	δ_H_ (*J* in Hz)		position	δ_C_	δ_H_ (*J* in Hz)		position	δ_C_	δ_H_ (*J* in Hz)
Ac	CH_3_	23.2	1.86 s	Gln^7^	NH		7.68 d (4.1)	Leu^12^	NH		7.62 d (7.9)	Aib^16^	NH		7.52 s	Gln^19^	NH		7.43 d (7.6)
	C=O	170.6			α CH	56.4	3.68		α CH	51.5	4.23		α C	55.9			α CH	53.5	3.89
Aib^1^	NH		8.55 s		β_1_ CH_2_	25.9	1.89		β_1_ CH_2_	39.8	1.41		β_1_ CH_3_	26.5	1.40		β_1_ CH_2_	26.9	1.69
	α C	55.4			β_2_ CH_2_	25.9	1.98		β_2_ CH_2_	39.8	1.74		β_2_ CH_3_	22.1	1.30		β_2_ CH_2_	26.9	1.79
	β_1_ CH_3_	26.5	1.29		γ_1_ CH_2_	31.2	2.11		γ CH	24.1	1.71		C=O	175.0			δ_1_ CH_2_	31.6	1.91
	β_2_ CH_3_	26.0	1.32		γ_2_ CH_2_	31.2	2.26		δ_1_ CH_3_	20.9	0.76 d (6.1)	Aib^17^	NH		7.52 s		δ_2_ CH_2_	31.6	2.03
	C=O	176.1			C(O)NH_2_	173.3	6.68 s, 7.00 s		δ_2_ CH_3_	22.9	0.81 d (6.1)		α C	56.0			C(O)NH_2_	173.3	6.56 s, 7.08 s
Ala^2^	NH		8.51 d (5.3)		C=O	173.8			C=O	172.9			β_1_ CH_3_	26.5	1.42		C=O	171.0	
	α CH	51.8	3.88	Aib^8^	NH		7.86 s	Aib^13^	NH		8.09 s		β_2_ CH_3_	25.7	1.35	Pheol^20^	NH		6.89 d (9.0)
	β_1_ CH_3_	16.4	1.27 d (7.0)		α C	55.6			α C	56.1			C=O	175.7			α CH	52.3	3.84
	C=O	174.8			β_1_ CH_3_	26.6	1.29–1.42		β_1_ CH_3_	23.0	1.29–1.40	Gln^18^	NH		7.55 d (5.0)		β_1_ CH_2_	36.7	2.48 dd (8.4, 13.5)
Aib^3^	NH		7.85 s		β_2_ CH_3_	22.9	1.29–1.42		β_2_ CH_3_	23.7	1.29–1.40		α CH	54.7	3.79		β_2_ CH_2_	36.7	2.84 dd (4.5, 13.5)
	α C	55.6			C=O	175.6			C=O	173.4			β_1_ CH_2_	26.5	1.87		C-1	139.1	
	β_1_ CH_3_	25.9	1.37	Val^9^	NH		7.19 d (6.9)	Pro^14^	α_1_ CH_2_	48.5	3.47		β_2_ CH_2_	26.5	1.92		C-2, C-6	129.2	7.22
	β_2_ CH_3_	22.9	1.29–1.40		α CH	62.2	3.62 t (7.5)		α_2_ CH_2_	48.5	3.66		δ_1_ CH_2_	31.7	2.15		C-3, C-5	127.9	7.19
	C=O	175.7			β CH	28.6	2.18		β CH_2_	25.5	1.80		δ_2_ CH_2_	31.7	2.26		C-4	125.8	7.13 dt (1.4, 7.2)
Ala^4^	NH		7.78 d (4.5)		γ_1_ CH_3_	19.1	0.90 d (6.5)		γ_1_ CH_2_	28.6	1.61		C(O)NH_2_	173.7	6.71 s, 7.11 s		γ_1_ CH_2_	63.0	3.25
	α CH	51.9	3.84		γ_2_ CH_3_	19.4	0.92 d (6.5)		γ_2_ CH_2_	28.6	2.16		C=O	172.0			γ_2_ CH_2_	63.0	3.29
	β_1_ CH_3_	16.3	1.27 d (7.0)		C=O	173.0			δ CH	62.5	4.29						OH		4.55 br s
	C=O	174.9		Aib^10^	NH		7.84 s		C=O	173.4									
Aib^5^	NH		7.81 s		α C	55.6		Val^15^	NH		7.52								
	α C	55.6			β_1_ CH_3_	25.9	1.29–1.42		α CH	61.1	3.71								
	β_1_ CH_3_	26.0	1.41		β_2_ CH_3_	22.7	1.29–1.42		β CH	28.6	1.71								
	β_2_ CH_3_	22.6	1.48		C=O	175.9			γ_1_ CH_3_	19.0	0.81 d (6.5)								
	C=O	172.6		Gly^11^	NH		8.15 t (5.9)		γ_2_ CH_3_	19.0	0.83 d (6.5)								
Aib^6^	NH		7.83 s		α_1_ CH_2_	43.8	3.44		C=O	175.4									
	α C	55.6			α_2_ CH_2_	43.8	3.69												
	β_1_ CH_3_	26.9	1.40		C=O	169.6													
	β_2_ CH_3_	26.0	1.31																
	C=O	176.6																	

The ^1^H NMR spectra of trichokonins VI (**1**) and VIII (**2**) displayed 25 amide proton signals (from δ_H_ 6.56 to 8.62), overlapping α-proton signals (from δ_H_ 3.43 to 4.29), acetyl protons (δ_H_ 1.93 for **1** and δ_H_ 1.86 for **2**), overlapping γ-proton signals (δ_H_ 1.41 to 1.90), overlapping α-aminoisobutyric acid and alanine methyl groups (from δ_H_ 1.26 to 1.49) as well as leucine and valine methyl groups from δ_H_ 0.76 to 1.01 (Tables 1 and 2). Analysis of ^13^C NMR data revealed 23 amide carbonyl signals (from δ_C_ 169.6 to 176.6), four sp^2^ carbons (from δ_C_ 125.8 to 139.1) and one γ-carbon (δ_C_ 63.0) belonging to the pheol^20^ residue, 20 tertiary or quaternary α-carbons (from δ_C_ 48.5 to 62.7), and other methylene and methyl carbons from δ_C_ 16.2 to 39.8 (Tables 1 and 2). Based on the HSQC, HMBC, HSQC-TOCSY, COSY, and ROESY experiments, the amino acid residues Ala^2^, Ala^4^, Ala^6^ (only for **2**), Gln^7^, Val^9^, Gly^11^, Leu^12^, Pro^14^, Val^15^, Gln^18^, Gln^19^, and Pheol^20^ could be confidently assigned (Figure 3). The Ac-Aib^1^ and the other Aib amino acid residues in the trichokonins VI and VIII were defined by analyses of HSQC and HBMC correlations, the latter helping to position the Aib residues by observing cross peaks from adjacent amino acids (Figure 3), along with ROESY correlations.

**Figure 3:**
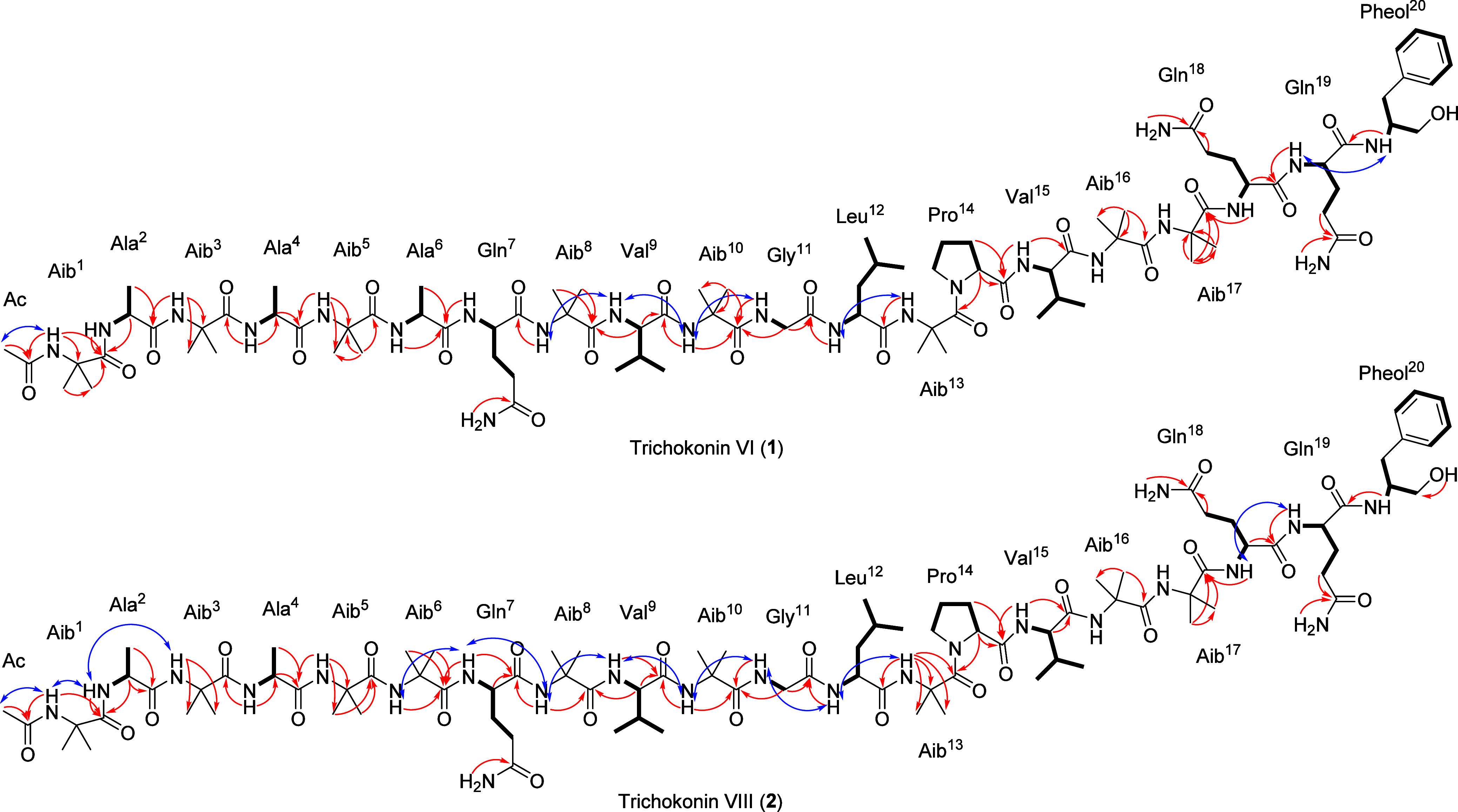
Key HMBC (red arrows), HSQC-TOCSY and COSY correlations (bold bonds), and NOESY (blue arrows) interactions for **1** and **2**.

Peptaibols exhibit circular dichroism spectra characteristic of helical peptide structures, with negative Cotton effects observed between 205 and 209 and 221–226 nm, and positive Cotton effect between 192 and 198 nm.^41^ Since peptaibol skeletons are very well structured by the formation of hydrogen bonds and by the presence of the Aib residues, peptaibol CD spectra generally show minimal dependence on solvent variations. For peptaibols **1**, **2**, and **6** (Figure 4), we verified that the CD spectra exhibit a minimum at 209–211 nm, an extended minimum at 220–222 nm referring to the n-p* type transition, and a positive maximum absorption at approximately 194 nm typically observed for a α-helical peptidic conformation.^41^ The CD spectra of peptaibols **1** and **2** are comparable in shape, position, and magnitude of the Cotton effect. CD spectra of compounds **3**–**5** (Figure 4) exhibit two minimum Cotton effect bands around 205–209 nm referring to p-p*-type transitions, accompanied by a discrete minimum between 220 nm referring to the n-p* type transition. A positive maximum near 194 nm, characteristic of a α-helix conformation,^41^ has also been observed, confirming that peptaibols **1**–**6** all have *a*-helix structures in solution.

**Figure 4:**
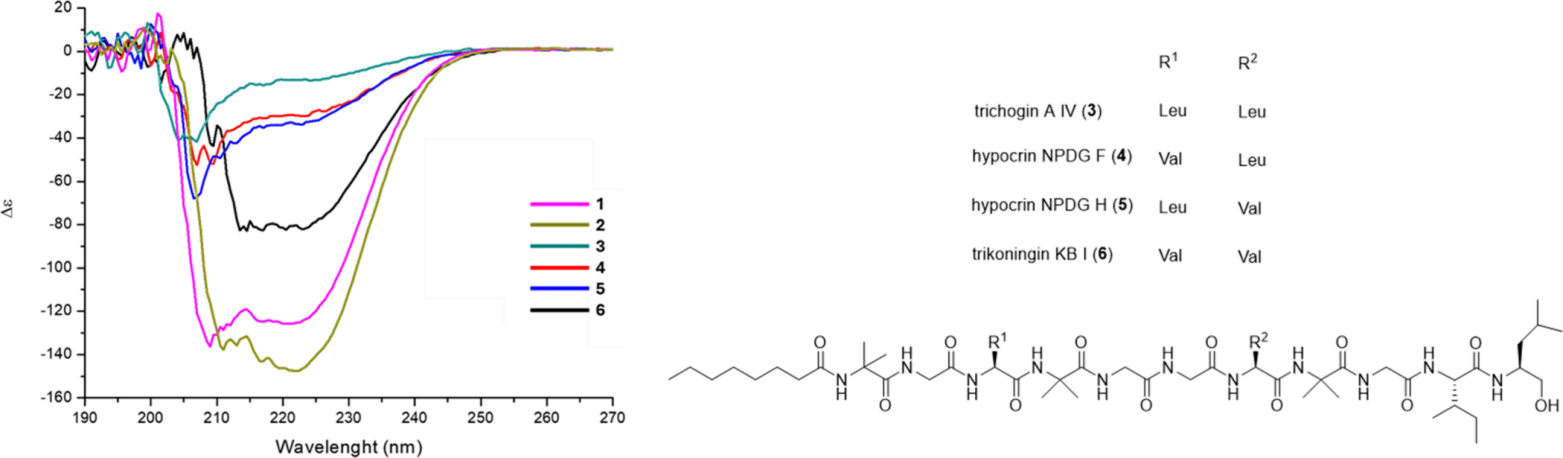
(Left) Circular dichroism spectra for peptaibols **1** *–* **6** in MeOH (*c* = 0.5 mg mL^–1^). Chemical structures of lipopeptaibols **3**–**6**.

### Genome Mining and Biosynthesis Gene Cluster Assignment of Trichokonins VI and VIII

The whole genome sequencing and processing of *Trichoderma* sp. L2-2 was performed by using Illumina and Nanopore technologies. The final assembly has 37,716,117 base pairs (bp) in 45 contigs, with a CG content of 47.6%. Analysis using fungiSMASH^42^ identified 36 biosynthetic gene clusters (BGCs) (Figure 5), including 8 related to NRPS biosynthesis, 4 for NRPS-like, 10 for T1PKS, 6 for terpenes, 1 for fungal RiPP-like, 1 for isocyanide-nrp, and 6 hybrid BGCs. The hybrid BGCs included 3 NRPSs/T1PKSs, 1 NRPS-like/T1PKS, 1 NRPS-like/fungal RiPP-like, and 1 fungal RiPP-like/terpene. Interestingly, from the 3 NRPSs/T1PKSs BGCs, only 1 BGC located on scaffold 1 and contig 2 (1.2) contained a hybrid polyketide nonribosomal peptide synthase (PKS-NRPS) gene with the domain organization of 21-module PKS-NRPS (Figures S41–S43), potentially corresponding to trichokonins VI (**1**) and VIII (**2**) biosynthesis. This BGC was named *trat* (GenBank accession no. PX023957).

**Figure 5:**
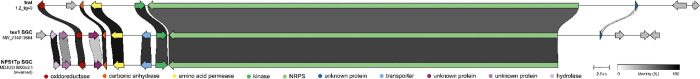
Clinker comparison of the peptaibol BGC identified in *Trichoderma* sp. L2-2 and other peptaibol BGCs.^45,46,48^

The *trat* BGC was refined using Augustus,^43^ and NCBI’s BLASTp tool^44^ was used to evaluate coding sequences for homology to characterized proteins, which resulted in putative function annotations (Table 3). The *trat* cluster revealed nine open reading frames that encode the PKS-NRPS TratS, two oxidoreductases (TratA and TratF), three transporter proteins (TratT, TratB and TratC), a phosphatase (TratD), a prenyltransferase (TratG), and one unknown protein. A closer inspection of TratS (69.6 kb PKS-NRPS) showed an acyl transferase (AT) domain in the loading module for the incorporation of the acetyl group in the *N*-terminus and a reductase domain in the last module for converting *C*-terminal l-Phe to l-phenol, essential for 20-res peptaibol trichokonins VI and VIII synthetase. Additionally, the homology search of TratS showed 76% of identity with Tex1 peptaibol NRPS from *Trichoderma virens* Gv29-8 related to the production of 18mer peptaibols.^45,46^

**Table 3:** Annotation and Comparison of the *trat* Biosynthetic Gene Cluster

gene	protein name	putative function	homologue	identity (%)	query cover (%)	*E*-value	score
*tratA*	TratA	aldo/keto reductase	T069G_08187	86.14	100	0	624
*tratT*	TratT	MFS general substrate transporter	K444DRAFT_652276	47.95	97	1e-162	485
*tratB*	TratB	carbonic anhydrase	T069G_08189	86.98	99	3e-106	311
*tratC*	TratC	amino acid permease	T069G_08190	93.77	100	0	999
*tratD*	TratD	Ppx/GppA phosphatase	T069G_08191	86.62	100	0	1016
*tratS*	TratS	NRPS	Tex1	76.96	100	0	26,904
*tratE*	TratE	unknown					
*tratF*	TratF	cytochrome P450	TGAM01_v204900	88.58	100	0	1077
*tratG*	TratG	*Ubia Prenyltransferase*	HER10_EVM0011648	47.43	98	1e-98	305

Detailed comparative gene cluster analysis of the putative peptaibol BGC (*trat*) was performed against known peptaibols BGCs using Clinker^47^ confirming high homology between the clusters (Figure 5). The NRPS gene Tex1 from *Trichoderma virens* ^45,46^ and NPS1_tp_ from *Trichoderma pleuroti* ^48^ related to the production of peptaibols showed synteny with NRPS TratF with high identity.

The biosynthesis of trichokonins VI and VIII therefore is herein hypothesized to start from the *trat* BGC (Scheme 1), showing the domain organization of the 21-module PKS-NRPS TratS. The coisolation of trichokonins VI and VIII indicates that module 6 of the NRPS is capable to get two different amino acids, the Aib for trichokonin VI and the Ala for trichokonin VIII.

**Scheme 1:**
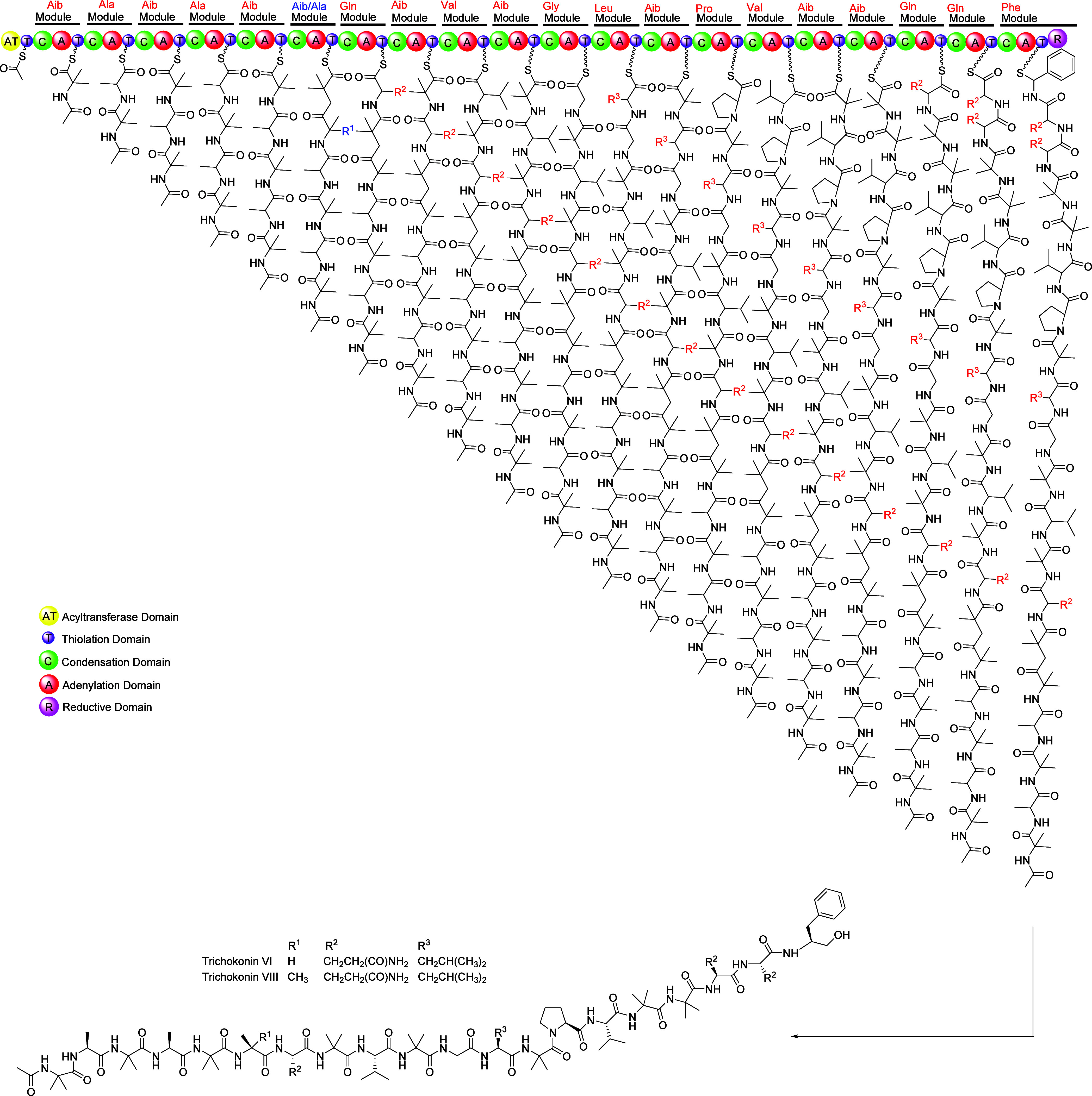
Proposed Biosynthesis for Trichokonins VI and VIII

Phylogenetic analyses of the A and C domains of TratS, Tex1, and NPS1_Tp_ (Figures 6 and 7) revealed greater similarity in module origin than in substrate specificity. There are some notable clades of more structurally distinct amino acid residues such as proline and glutamine, but the remaining residues demonstrate significant overlap between clades. The A and C domains from module 6 (TratS A6 and TratS C6), which have relaxed substrate specificity, formed clades with A and C domains related to the Ala, Alib, and Vxx amino acid residues. In a comparison of the phylogenetic distribution between the A and C domains, a difference can be noted in the designation of domains TratS A2 and TratS A3.

**Figure 6:**
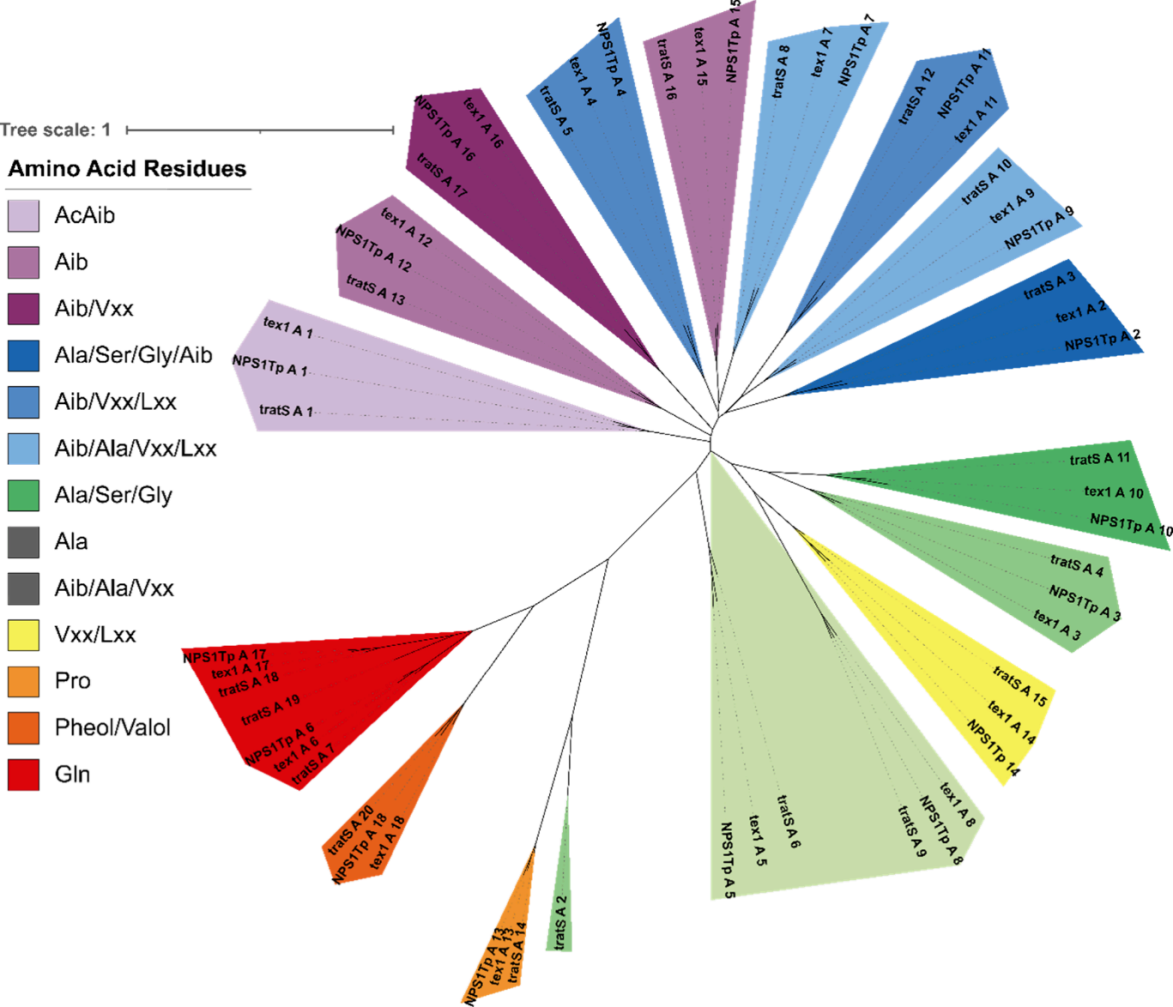
Phylogenetic analysis of the A domains from TratS, Tex1, and NPS1_Tp_, color coded by substrate specificity. Vxx refers to valine or isovaline, and Lxx refers to leucine or isoleucine.

**Figure 7:**
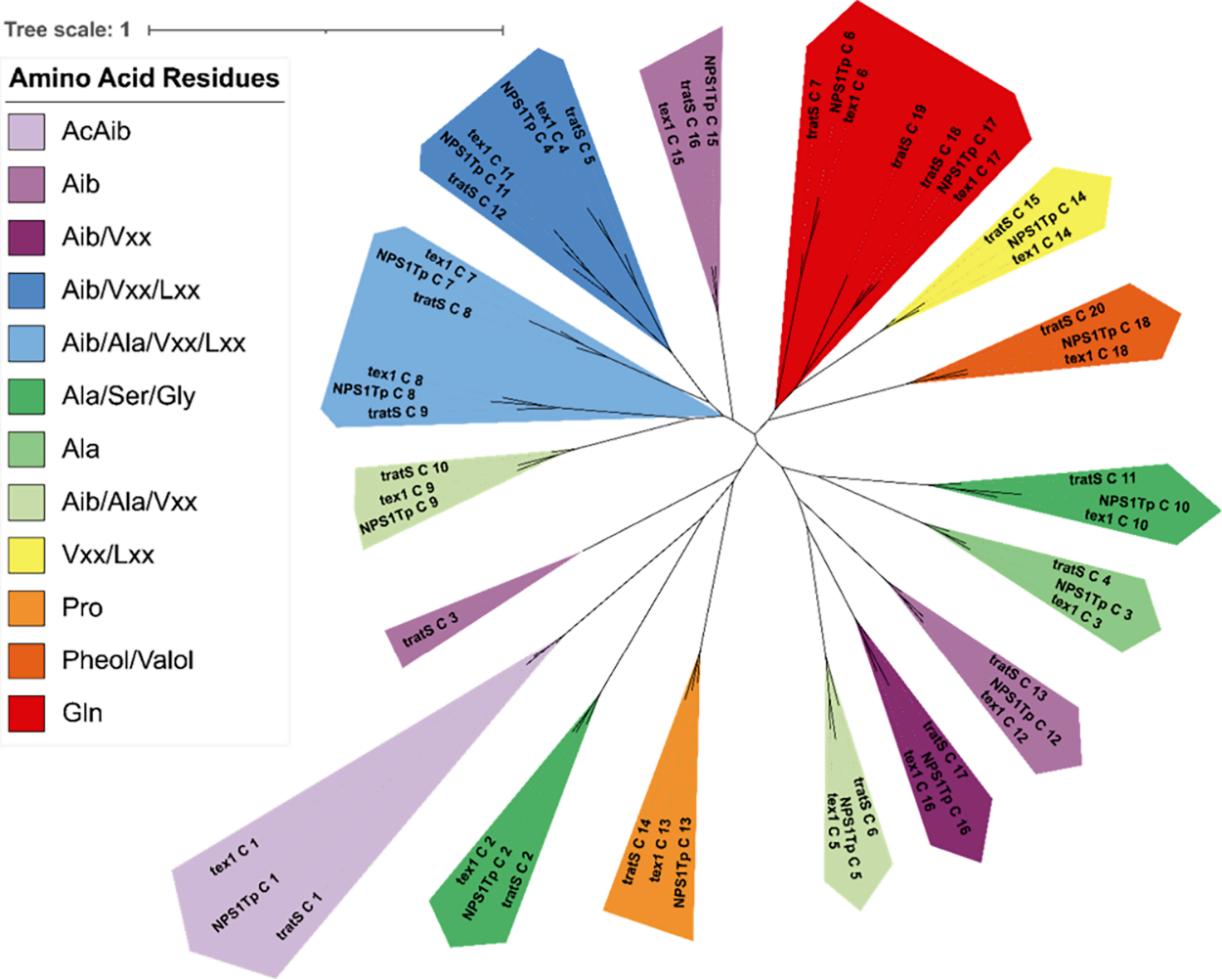
Phylogenetic analysis of the C domains from TratS, Tex1, and NPS1_Tp_, color coded by substrate specificity. Vxx refers to valine or isovaline, and Lxx refers to leucine or isoleucine.

### Antiplasmodial and Cytotoxic Activity of Trichokonins VI and VIII

Trichokonins VI (**1**) and VIII (**2**) were tested *in vitro* against *Plasmodium falciparum* (*P. falciparum*) (3D7 strain, chloroquine-sensitive) and human hepatocellular carcinoma cells (HepG2) to assess their antiplasmodial and cytotoxic activities (Table 4).

**Table 4:** Assessment of *In Vitro* Antiplasmodial Activity (IC_50_), Cytotoxicity (CC_50_), and Selectivity Index Values of Trichokonins VI (1) and VIII (2), Trichogin A IV (3), and Hypocrin NPDG F (4)

compound	IC_50_^3D7^ (μM) Mean ± SD	CC_50_^HepG2^ (μM) Mean ± SD	**SI** ^a^
trichokonins VI (**1**)	1.0 ± 0.2	28 ± 3	28
trichokonins VIII (**2**)	0.9 ± 0.2	15 ± 3	16
trichogin A IV (**3**)	5.7 ± 0.6	>25	>4
hypocrin NPDG F (**4**)	4.4 ± 0.9	>12	>3
artesunate^b^	15 ± 5 nM	nd	nd
pyrimethamine^b^	51 ± 9 nM	nd	nd

a SI = CC_50_/IC_50._

b Positive control for inhibition; nd = not determined.

Trichokonins analogues demonstrated inhibitory activity against *P. falciparum* at low micromolar concentrations (IC_50_s = 0.9–5.7 μM). Notably, trichokonins VI (**1**) and VIII (**2**) were the most potent derivatives, showing IC_50_s values of 1.0 and 0.9 μM, respectively (Table 4). These results are consistent with previously reported antiplasmodial activity of peptaibols.^34,49^ Furthermore, trichokonins exhibited moderate cytotoxic activity against HepG2 cells (CC_50_^HepG2^ > 12 μM) (Table 4). Particularly, trichokonins VI (**1**) and VIII (**2**) demonstrated CC_50_s values of 28 and 15 μM, respectively, resulting in selectivity indexes (SI) of 28 and 16, respectively. Compounds with SI values higher than 10 are considered promising for further investigations of new candidates for antimalarial drugs.^50^

Large-size peptaibols, with 18–20 amino acid residues, are numerous, with tens of such peptides been reported in the literature.^51–53^ However, as far as we know, our results are the first to report antiplasmodial activity for large-size peptaibols with 20 amino acid residues. Since this activity is similar to previously reported peptaibols,^34,49^ and that the structures of these compounds are similar, we suppose that the mechanism of action of our compounds is similar as well,^49^ but this hypothesis remain to be confirmed. Although many of such large peptides have been isolated and identified, to the best of our knowledge, there are no reports on the structures of 20 amino acid residue peptaibols completely assigned by NMR, HRMS, MS/MS, ECD, and biosynthesis gene cluster analysis. Even for MS and NMR analyses together, only few examples are found in the literature.^54^ Considering that more than 1000 peptaibols have been isolated to date, only very few of these peptides have had biosynthesis gene clusters investigated.^55–61^ A common feature observed for peptaibols by both peptide hydrolysis and amino acid derivatization analysis as well as by genomic analyses is the rare occurrence of d-amino acid residues in these peptides. Our results align with the genomes of these peptaibols, contributing to understand the evolution of these fungal NRPS peptides, which appear to exert relevant ecological functions for fungi adaptation.^62^

## Conclusions

In summary, the large peptaibols trichokonins VI (**1**) and VIII (**2**) have been fully characterized by analysis of NMR, HRMS/MS, and ECD data for the first time. Analysis of *Trichoderma* sp. L2-2 enabled the assignment of the biosynthesis gene clusters of both peptaibols, which also displayed antiplasmodial activity against *P. falciparum* at the low micromolar range and attractive selectivity indexes.

## Experimental Section

### General Experimental Procedures

UV spectra were recorded on a Shimadzu UV-3600 instrument. Specific rotations were performed in a Polartronic H Schimidt+Haensch polarimeter with a 10 dm cuvette. CD spectra were obtained by using a Jasco J-815 spectropolarimeter. ATR-IR spectra were acquired with Shimadzu equipment, model IRAffinity. NMR spectra were obtained at 25 °C, in diluted aliquots in DMSO-*d*_6_ or in MeOH-*d*_4_, using TMS or the nondeuterated residual solvent signal as an internal standard, using a Bruker AVANCE III 14.1 T instrument operating at either 600 MHz (^1^H) or 150 MHz (^13^C), equipped with a cryo-probe TCI (^1^H/^13^C/^15^N) of 5 mm with ATMA and field gradient in z. HRMS analyses were performed by Waters UPLC Xevo G2-XS q-TOF equipment using a C_18_ reverse phase column (Waters ACQUITY UPLC BEH, 2.1 mm × 100 mm, 1.7 μm). Elution was performed with a mobile phase composed of (A) H_2_O + 0.1% formic acid and (B) MeCN + 0.1% formic acid. The gradient used was: 0–9.0 min linear gradient from 10 to 100% B, from 9.0–10.0 min 10% B was maintained for column reconditioning. The mass spectrometer was adjusted according to the following parameters: MSE continuous mode, an *m*/*z* detection range of 200–5000 Da, electrospray ionization mode ESI, an acquisition time of 0.2 s, and a collision energy ramp of 20–30 V. HPLC-PDA-ELSD-MS analyses were performed with a Waters Alliance 2695 instrument connected online with a Waters 2996 PDA detector, followed by a Micromass ZQ 2000 detector with an ESI interface. The mass spectrometer operation was adjusted using the following conditions: capillary voltage, 3.00 kV; source block temperature, 100 °C; desolvation temperature, 350 °C; voltage cone, 25 V; electrospray ionization (ESI), operating in positive and negative modes, detection in a 400–1400 Da range with total ion chromatogram (TIC). Desolvation and cone gas flows were adjusted to 50 and 350 L h^–1^, respectively, with a N_2_ source. Data recording and processing were performed using Empower 2.0 software. HPLC-PDA-ELSD-MS analyzes were acquired using a C_18_ reversed-phase column (Waters X-terra, 250 × 4.6 mm, 5 μm) with an eluent flow rate of 1 mL min^–1^. Elution was performed with a mobile phase composed of (A) H_2_O + 0.1% formic acid and (B) 1:1 (v/v) MeOH/MeCN + 0.1% formic acid. The gradient used was: 0–1.0 min with 10% B, linear gradient up to 100% B from 1.0–30.0 min, from 30.0–35.0 min 100% B was maintained and from 35.0 to 40.0 min, 10% B was maintained for column reconditioning, totaling 40 min of analysis. The sample volume injected was 20 μL of a solution with a concentration of 1.0–2.5 mg L^–1^.

### *Trichoderma* sp. L2-2 Identification

The *Trichoderma* sp. L2-2 sample was isolated from a lichen substrate collected at King George Island (Admiralty Bay, Punta Plaza, Antarctica). The fungus was identified by analysis of the ITS genome sequence, which was deposited in GenBank with accession number MG813420.1. The fungal strain is stored in the Laboratory of Environmental and Industrial Mycology (LAMAI) collection associated with the Central of Microbial Resources of São Paulo State University CRM-UNESP (UNESP, Rio Claro, SP, Brazil). The fungus accession numbers are LAMAI 605 and CRM 55.

### Extraction and Isolation

The *Trichoderma* sp. L2-2 strain was grown in 4 L of malt 2% medium at 15 °C with shaking (150 rpm). After 7 days of growth, the fungal cultures were filtered and blended with EtOAc. The organic solvent was separated from cultures by liquid–liquid partitioning. The EtOAc fraction was evaporated and solubilized in MeOH. The MeOH fraction was defatted with *n*-hexane. The resulting MeOH soluble fraction was named AT1M (0.77 g). The AT1M fraction was subjected to a solid-phase extraction (SPE) using prepacked silica gel columns derivatized with cyanopropyl groups. The following elution gradient was used: 100% Hex (AT1MA1), 100% CH_2_Cl_2_ (AT1MA2), CH_2_Cl_2_:EtOAc (1:1, v/v) (AT1MA3), 100% EtOAc (AT1MA4), EtOAc:MeOH (1:1, v/v) (AT1MA5), and 100% MeOH (AT1MA6). The fractions obtained (AT1MA1 to AT1MA6) were analyzed by HPLC-PDA-MS. Analysis of fraction AT1M5 by HPLC-PDA-MS indicated compounds with molecular weights higher than 500 Da.

Separation of the AT1M5 fraction (337.1 mg) was performed on a column packed with Sephadex LH-20 eluted with MeOH, collecting fractions of 4.5 mL. The fractions obtained were pooled by chromatographic similarity observed by TLC analysis [EtOAc/Hex (3:7, v/v), EtOAc/Hex (6:4, v/v), 100% EtOAc, MeOH/CH_2_Cl_2_ (1:9, v/v), MeOH/CH_2_Cl_2_ (3:7, v/v)]. A total of 14 fractions were obtained and named from AT1M5A to AT1M5P. Fraction AT1M5O (176.4 mg) was purified by semipreparative HPLC using a C_8_ HPLC column (GL Sciences InertSustain, 250 × 4.6 mm, 5 μm) and an isocratic eluent of MeOH/H_2_O (82:12, v/v) with 0.1% formic acid. The chromatographic separation was performed over 30 min (flow rate of 2.0 mL min^–1^ and detection by ELSD) yielding pure compounds TK-VI (**1**, 69.5 mg) and TK-VIII (**2**, 52.3 mg) (see the Supporting Information for details). The peptaibols TK-VI (**1**) and TK-VIII (**2**) were identified by NMR, HRMS, and IR analyses.

AT1M3 fraction (50.6 mg) was resubmitted to a solid-phase extraction using a prepacked column with a stationary phase of silica gel derivatized with diol groups. The sample was eluted with 10 mL of a gradient of EtOAc and Hex (from 20 to 100% of EtOAc), and 10 mL of a gradient of MeOH and EtOAc (from 20 to 100% MeOH) collecting fractions of 2.5 mL. The fractions obtained were grouped according to the polarity of the compounds observed in TLC analysis, resulting in 11 fractions named AT1M3A to AT1M3K. Fraction AT1M3I (13.7 mg) was purified on an analytical column with a C_8_ stationary phase column (GL Sciences InertSustain, 250 × 4.6 mm, 5 μm), isocratic eluent MeOH/H_2_O (76:24, v/v) with 0.1% formic acid (flow rate of 1.0 mL min^–1^ and detection by ELSD). Four pure compounds were obtained: trichogin A IV (**3**, 5.1 mg), hypocrin NPDG F (**4**, 1.6 mg), hypocrin NPDG H (**5**, 1.8 mg), and trikoningin KB I (**6**, 0.4 mg). The peptaibols **3**–**5** were identified by NMR, HRMS, and IR analyses, and compound **6** was identified by HRMS/MS.

### Marfey’s Analysis

In a 2 mL glass vials, to a 1.0 mg of each amino acid standard were added 200 μL of H_2_O, 80 μL of 1 M NaHCO_3_ aqueous solution, and 400 μL of a solution of 1% Marfey’s reagent (*N*_α_-(2,4-dinitro-5-fluorophenyl)-l-alaninamide) in acetone. The reaction mixtures were stirred at 40 °C for 1 h. After this period, the reactions were quenched by the addition of 40 μL of a 2 N HCl aqueous solution. The resulting reaction mixture was dried, and the solid formed was dissolved in 2.0 mL of MeOH. After, 500 μL of these solutions were transferred to new vials and had their volumes completed to 1.0 mL with MeOH. These solutions were analyzed individually by HPLC-PDA-MS (as described in general procedures experiments). In addition, aliquots of all derivatized standards were combined to provide a mixed standard, which was: mixture 1: (L)-Leu and (L)-Ile; mixture 2: (L)-Val, (L)-Leu and (L)-Ile; mixture 3: (L)-Pro, (L)-Gln, (L)-Ala, (L)-Val and (L)-Leu. HPLC-PDA-MS analyses was performed using as mobile phase the gradient: 0–1.0 min with 20% B, linear gradient up to 100% B from 1.0–30.0 min, from 30.0–35.0 min maintained in 100% B, and finally from 35.0–40.0 min maintained in 10% B for column reconditioning.

To generate hydrolyzed and derivatized peptaibols, approximately 0.3–0.5 mg of compounds **1**–**5** were weighed separately into 2 mL vials. To these flasks was added 0.5 mL of an aqueous solution of HCl 6N. The reaction remained under stirring for 24 h, at 90 °C. Afterward, the solvent was evaporated, and to each compound, 100 μL of H2O, 40 μL of 1 M NaHCO_3_ aqueous solution, and 200 μL of a solution of 1% Marfey’s reagent in acetone were added. The reaction mixtures were stirred at 40 °C for 1 h. After this period, the reactions were quenched by the addition of 20 μL of 2 N HCl. The reaction mixtures were dried and dissolved in 1.0 mL of MeOH. Each hydrolyzed and derivatized compound was analyzed individually by HPLC-PDA-MS using the same mobile phase used for the analysis of the mixtures (chromatograms available at the Supporting Information).

### Genome Sequencing and Mining

*Trichoderma* sp. L2-2 genomic DNA was purified by phenol-chloroform extraction. The UV–vis absorption spectrum of the DNA solution was obtained on a microvolume spectrophotometer (DeNovix DS-11), with 1 μL of solution added to the sample surface, using the preconfigured method for double-stranded DNA analysis. The DNA integrity was checked by electrophoresis analysis on 1% agarose gel. Genomic DNA was prepared for sequencing by employing an Illumina DNA Prep kit following the instructions of the manufacturer. Sequencing the library was performed on an Illumina NovaSeq6000 instrument, employing paired end, 2 × 150 base sequencing. Sequence data were processed for *de novo* assembly using the CLC genomics workbench (v22) using default parameters. DNA sequencing and assembly were performed by the University of Illinois at Chicago Core for Research Informatics (UICCRI).

Secondary metabolite biosynthesis gene clusters were preliminary identified using antiSMASH,^34^ and the BGC related to the production of trichokonin VI and VIII was reannotated using the Augustus^43^ platform with *Fusarium graminearum* as a reference. This BGC was named *trat* (GenBank accession no. PX023957). Coding sequences were investigated using NCBI’s BLASTp.^44^ Sequence data were visualized using Geneious Prime.

The synteny analysis of the *Trichoderma* sp. L2-2 and known peptaibols’ BGCs was performed by Clinker using default parameters and visualized by the interactive clustermap.js.^47^

### Maintenance of *P. falciparum* *In Vitro* Cultures

Continuous *in vitro* cultures of *P. falciparum* (3D7 strain) were maintained following a modified published method.^63^ The procedure for this assay followed the protocol recently reported by us.^64^

### SYBR Green I Growth Inhibition Assay against *P. falciparum* Asexual Forms

Compounds were diluted to a stock concentration of 20 mM in 100% DMSO prior to experiments and stored at −20 °C. Parasites were synchronized using sterile 5% (m/v) d-sorbitol treatment for 10 min at 37 °C to enrich ring-stage parasites.^65^ All tests were conducted in duplicates using a SYBR Green I method with results compared to control cultures.^66^ Concentration–response curves were generated, and half-maximal inhibitory concentration (IC_50_^3D7^) values for each compound were determined by nonlinear regression analysis (Figures S44–S46). Each IC_50_^3D7^ value reflects the mean and standard deviations from at least two independent experiments. The detailed protocol used was recently described by us.^64^

### Cultivation of Human Hepatocellular Carcinoma Cells (HepG2 Cell Line) and Cytotoxicity Evaluation

The cytotoxic effects of trichokonins **1**–**4** were evaluated against the human hepatocellular carcinoma cell line (HepG2). The assay procedure was conducted according to a previously published protocol.^64^ Concentration–response curves were generated, and half-maximal inhibitory concentration (CC_50_) values for each compound were calculated by using nonlinear regression analysis (Figures S44–S46). Each CC_50_ value is expressed as the mean ± standard deviation from at least two independent experiments. The selectivity index (SI) was determined by calculating the ratio of CC_50_ to IC_50_^3D7^.

## Supplementary Material



## Data Availability

Raw NMR data have been provided within the NP-MRD platform. NP-MRD Deposit data for the compounds: trichokonin VI NP-Card ID: NP0351250; trichokonin VIII NP-Card ID: NP0351251; trichogin A IV NP-Card ID: NP0342304; hypocrin NPDG F NP-Card ID: NP0351252; hypocrin NPDG H NP-Card ID: NP0351253.

## Notes

NMR, HRMS, ECD, UV spectra of trichokonins VI and VIII; UPLC-HRMS analysis of Marfey AA derivatives; sequencing and biosynthesis gene cluster data of trichokonins VI and VIII; and concentration–response curves of trichokonins VI and VIII against *P. falciparum* (PDF)
