# Supplementary material for: NMR Analysis and Assignment of a Biosynthesis Gene Cluster for Trichokonins VI and VIII, Antiplasmodial Large Peptaibols Produced by a Trichoderma sp. Fungus
Source: ACS Omega. 2025 Oct 10;10(41):48293–307. doi: 10.1021/acsomega.5c05271 (PMC12547593; doi:10.1021/acsomega.5c05271)
Supplement: Supplementary file 1 [file ao5c05271_si_001.pdf]

Supporting Information

for

NMR Analysis and Assignment of Biosynthesis Gene Cluster for Trichokonins VI and VIII, Antiplasmodial Large Peptaibols Produced by a *Trichoderma* sp. Fungus

Ariane F. Bertonha,[†‡] David E. Williams,[⊥] Karen J. Nicacio,[†,^] Marcelo R. de Amorim,[†,*] Sydney M. Schoellhorn,[‡,*] Anna Caroline C. Aguiar,[O] Talita Alvarenga Valdes,[▽] Giovana Rossi Mendes,[▽] Igor M. R. Moura,[▽] Lamonielli F. Michaliski,[†] Matheus Gotha,[†] Caue A. W. Zuccarino,[†] Lara D. Sette,[§] Antônio G. Ferreira,[∥] Raymond J. Andersen,[⊥] Rafael Victorio Carvalho Guido,[▽]* Roberto G. S. Berlinck[†]*

[†]Instituto de Química de São Carlos, Universidade de São Paulo, 13560-970, São Carlos, SP, Brazil.
[⊥]Departments of Chemistry and EOAS, University of British Columbia, 2036 Main Mall, Vancouver, BC V6T 1Z6, Canada.
[‡]Brazilian Biosciences National Laboratory (LNBio), Brazilian Center for Research in Energy and Materials (CNPEM), 13083-970, Campinas, SP, Brazil.
[^]Universidade Federal de Mato Grosso (UFMT), 78060-900, Cuiabá, MT, Brazil.
[‡]Department of Chemistry and BioDiscovery Institute, University of North Texas, 1155 Union Circle, Denton, TX, 76203, USA.
[O]Department of Microbiology, Immunology and Parasitology, Federal University of São Paulo, 04023-062, São Paulo, SP, Brazil.
[§]Departamento de Biologia Geral e Aplicada, Universidade Estadual Paulista (UNESP), Instituto de Biociências, Rio Claro, SP, Brazil.
[∥]Departamento de Química, Universidade Federal de São Carlos, 13565-905, São Carlos, SP, Brazil.
[▽]São Carlos Institute of Physics, University of São Paulo, 13566-590, São Carlos, SP, Brazil.

Table of Contents

Figure S1. ^{1}H NMR spectrum of trichokonin VI (**1**) (DMSO-d_6, 600 MHz). S5
Figure S2. ^{13}C NMR spectrum of trichokonin VI (**1**) (DMSO-d_6, 151 MHz). S6
Figure S3. COSY spectrum obtained for trichokonin VI (**1**) (DMSO-d_6, 600 MHz). S7
Figure S4. HSQC-TOCSY spectrum obtained for trichokonin VI (**1**) (DMSO-d_6). S8
Figure S5. HSQC spectrum obtained for trichokonin VI (**1**) (DMSO-d_6). S9
Figure S6. HMBC spectrum obtained for trichokonin VI (**1**) (DMSO-d_6, 600 MHz). S10
Figure S7. NOESY spectrum obtained for the trichokonin VI (**1**) (DMSO-d_6). S11
Figure S8. HRMS/MS spectrum obtained for the fragmentation of the ion *m/z* 1163.67 of trichokonin VI (**1**). (**A**) ESI+, 60 V; (**B**) ESI+, 30 V. S12
Figure S9. HRMS/MS spectrum obtained for the fragmentation of the ion *m/z* 774.45 of trichokonin VI (**1**). (**A**) ESI+, 60 V; (**B**) ESI+, 30 V. S13
Figure S10. IR spectrum of the trichokonin VI (**1**) (cm^{-1}). S14
Figure S11. UV absorption spectrum of the trichokonin VI (**1**) (MeOH, c = 0.025 mg mL^{-1}). S15
Figure S12. CD spectrum of trichokonin VI (**1**) (MeOH, c = 0.15 mg mL^{-1}). S16
Figure S13. HPLC chromatogram of trichokonin VI (**1**) monitored at a wavelength of 210 nm, acquired using a C_{18} reversed phase column (Waters® X-terra, 250 × 4.6 mm, 5 µm) with a mobile flow rate of 1 mL min^{-1}. S17
Figure S14. ^{1}H NMR spectrum of trichokonin VIII (**2**) (DMSO-d_6, 600 MHz). S18
Figure S15. ^{13}C NMR spectrum of trichokonin VIII (**2**) (DMSO-d_6, 151 MHz). S19
Figure S16. COSY spectrum obtained for trichokonin VIII (**2**) (DMSO-d_6, 600 MHz). S20
Figure S17. HSQC-TOCSY spectrum obtained for trichokonin VIII (**2**) (DMSO-d_6). S21
Figure S18. HSQC spectrum obtained for trichokonin VIII (**2**) (DMSO-d_6). S22
Figure S19. HMBC spectrum obtained for trichokonin VIII (**2**) (DMSO-d_6). S23
Figure S20. ROESY spectrum obtained for the trichokonin VIII (**2**) (DMSO-d_6). S24
Figure S21. HRMS/MS spectrum obtained for the fragmentation of the ion *m/z* 1177.69 of trichokonin VIII (**2**). (**A**) ESI+, 60 V; (**B**) ESI+, 30 V. S25
Figure S22. HRMS/MS spectrum obtained for the fragmentation of the ion *m/z* 774.45 of trichokonin VIII (**2**). (**A**) ESI+, 60 V; (**B**) ESI+, 30 V. S26
Figure S23. IR spectrum of the trichokonin VIII (**2**) (cm^{-1}). S27
Figure S24. UV absorption spectrum of the trichokonin VIII (**2**) (MeOH, c = 0.025 mg mL^{-1}). ...S28
Figure S25. CD spectrum of trichokonin VIII (**2**) (MeOH, c = 0.15 mg mL^{-1}). S29

Figure S26. HPLC chromatogram of trichokonin VIII (**2**) monitored at a wavelength of 210 nm, acquired using a C_{18} reversed phase column (Waters® X-terra, 250 × 4.6 mm, 5 μm) with a mobile flow rate of 1 mL min^{-1}. S30
Figure S27. Chromatogram of Marfey reaction with Val (280 nm), UV and MS spectrum (ESI^-)....S31
Figure S28. Chromatogram of Marfey reaction with Ile (280 nm), UV and MS spectrum (ESI^-)....S32
Figure S29. Chromatogram of Marfey reaction with Leu (280 nm), UV and MS spectrum (ESI^-)....S33
Figure S30. Chromatogram of Marfey reaction with Pro (280 nm),UV and MS spectrum (ESI^-)....S34
Figure S31. Chromatogram of Marfey reaction with Ala (280 nm), UV and MS spectrum (ESI^-)....S35
Figure S32. Chromatogram of Marfey reaction with Gln (280 nm), UV and MS spectrum (ESI^-)....S36
Figure S33. Chromatogram of mixture 1, containing: Leu and Ile, at 280 nm, UV and MS spectrum (ESI^-). S37
Figure S34. Chromatogram of mixture 2, containing: Val, Leu and Ile, at 280 nm, UV and MS spectrum (ESI^-). S38
Figure S35. Chromatogram of mixture 3, containing: Val, Leu, Pro, Ala and Gln, at 280 nm, UV and MS spectrum (ESI-). S39
Figure S36. Chromatogram of the Marfey reaction for the hydrolyzed trichokonin VI (**1**) (280 nm), UV and MS spectrum (ESI^+)....S40
Figure S37. Chromatogram of the Marfey reaction for the hydrolyzed trichokonin VIII (**2**) (280 nm), UV and MS spectrum (ESI^+)....S41
Figure S38. Chromatogram of the Marfey reaction for the hydrolyzed trichogin A IV (**3**) (280 nm), UV and MS spectrum (ESI+). S42
Figure S39. Chromatogram of the Marfey reaction for the hydrolyzed hypocrin NPDG F (**4**) (280 nm), UV and MS spectrum (ESI+). S43
Figure S40. Chromatogram of the Marfey reaction for the hydrolyzed hypocrin NPDG H (**5**) (280 nm), UV and MS spectrum (ESI+). S44
Figure S41. The BGC overview by fungiSMASH. Scaffold 1 and contig 2 (1.2) has 21 modules for peptaibol biosynthesis. S45

Figure S42. The 21-module PKS-NRPS from the putative peptaibol BGC 1.2 by fungiSMASH. AT: Acyltransferase domain; CP: carrier protein domain; C: Condensation domain; A: Adenylation domain. S45

Figure S43. Synthaser analysis of domains contained within peptaibol PKS-NRPS enzymes with different size of peptaibols. KS: ketosynthase domain AT: acyltransferase domain; ACP: acyl carrier protein domain; C: condensation domain; A: adenylation domain; T: thiolation domain; R: terminal reductase domain S45

Figure S44. Representative concentration-response curves of (**A**) Trichokonins VI (**1**) and (**B**) VIII (**2**) against P. falciparum (3D7 strain, chloroquine-sensitive). Representative concentration-response curves of (**C**) Trichokonins VI (**1**) and (**D**) VIII (**2**) against human hepatocellular carcinoma cells (HepG2 cell line) S46

Figure S45. Representative concentration-response curves of (**A**) hypocrin NPDG F (**4**) and (**B**) trichogin A IV (**3**) against *P. falciparum* (3D7 strain, chloroquine-sensitive). Representative concentration-response curves of (**C**) hypocrin NPDG F (**4**) and (**D**) trichogin A IV (**3**) against human hepatocellular carcinoma cells (HepG2 cell line). S47

Figure S46. Representative concentration-response curves of positive controls (**A**) artesunate and (**B**) pyrimethamine against *P. falciparum* (3D7 strain, chloroquine-sensitive). S48

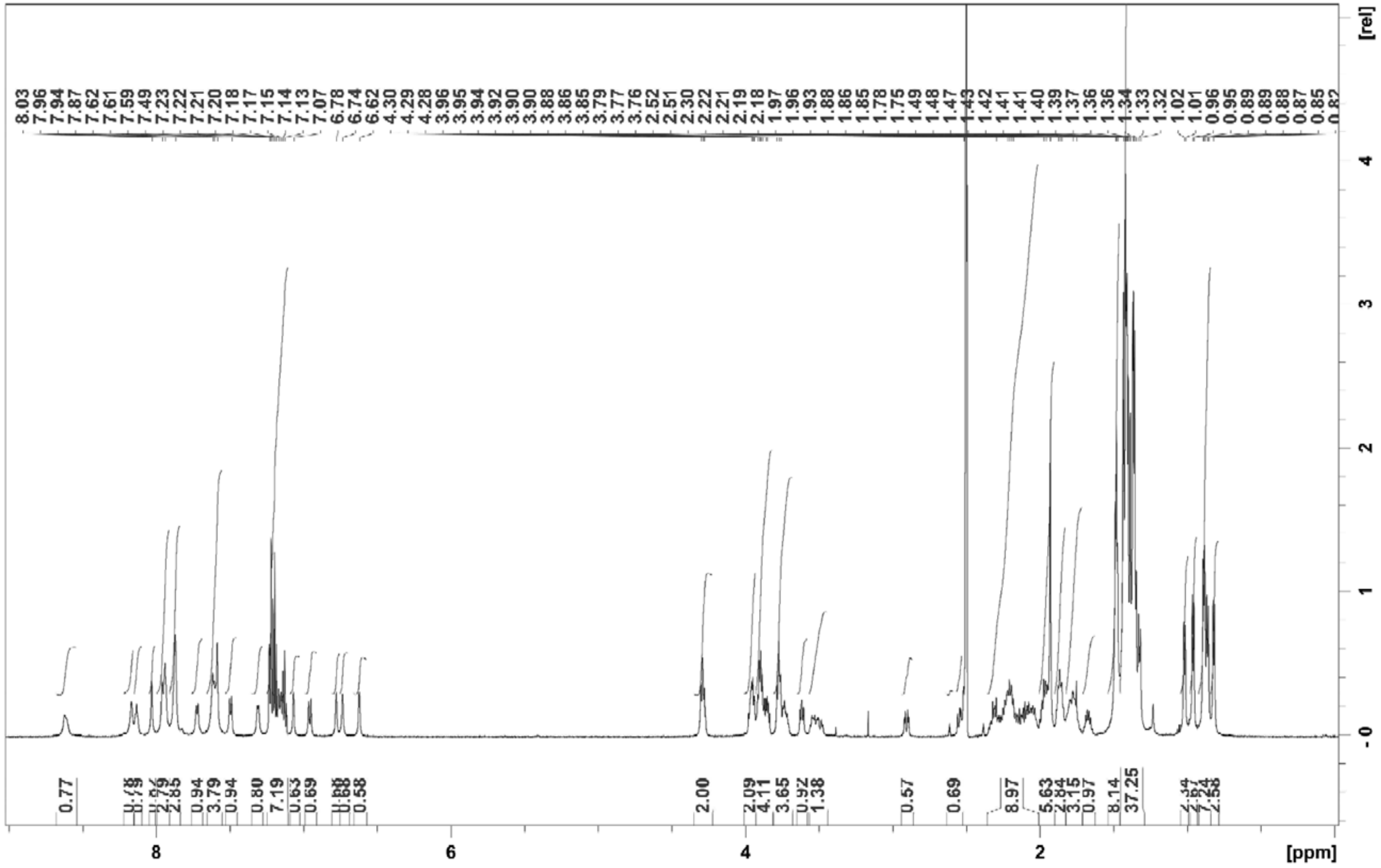

Figure S1. ^{1}H NMR spectrum of trichokonin VI (**1**) (DMSO-d_6, 600 MHz).

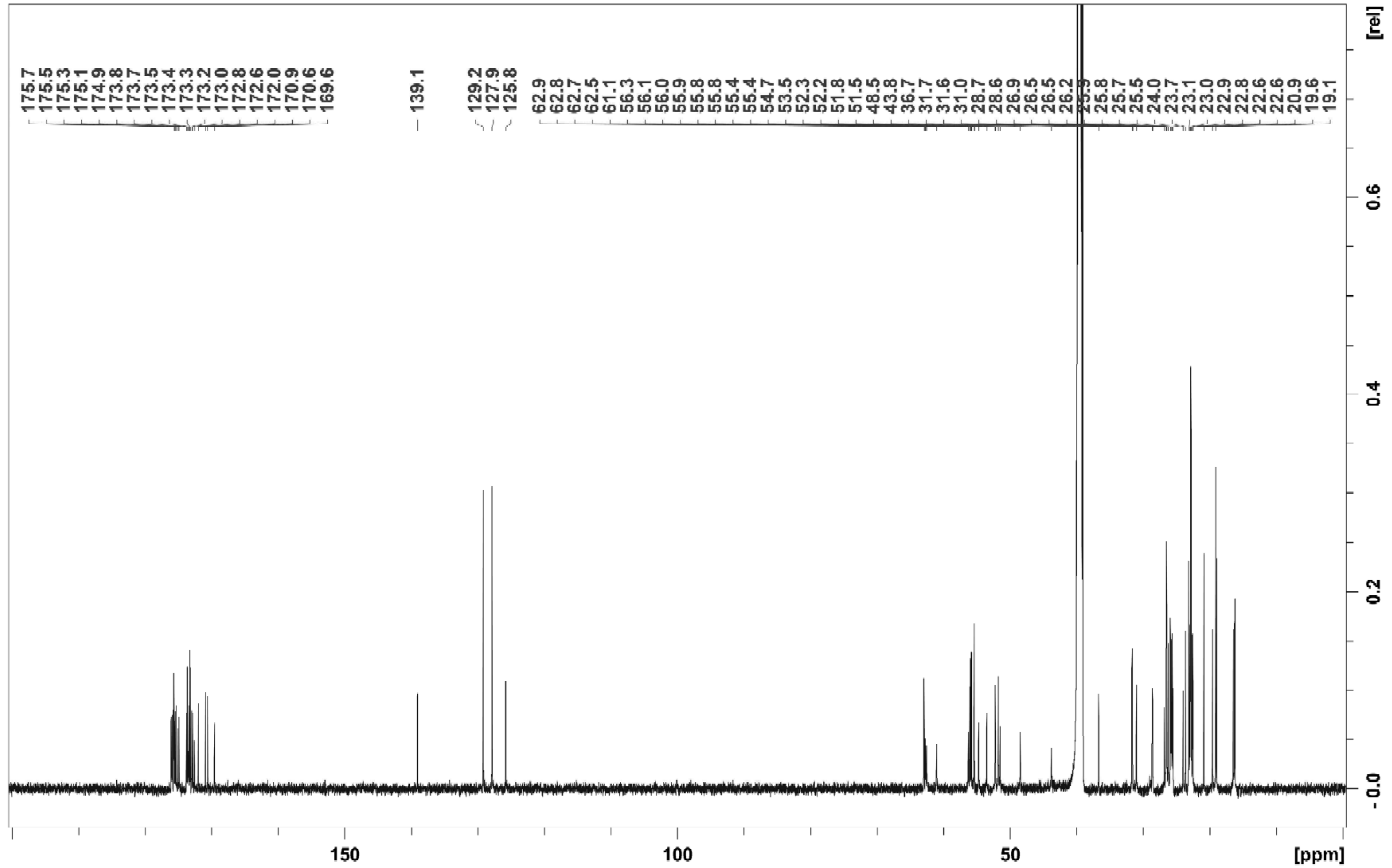

Figure S2. ^{13}C NMR spectrum of trichokonin VI (**1**) (DMSO-d_6, 151 MHz).

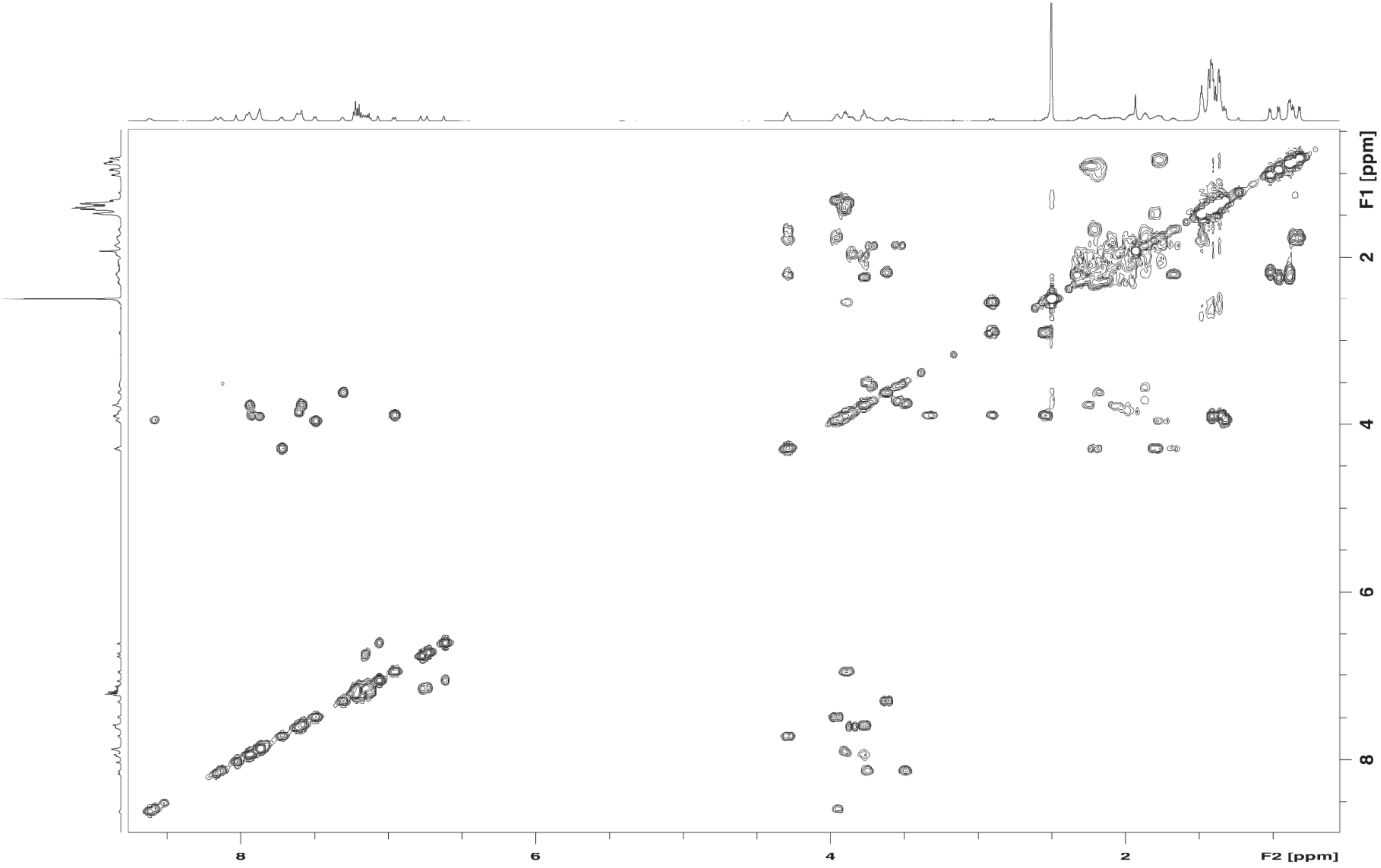

Figure S3. COSY spectrum obtained for trichokonin VI (**1**) (DMSO-d_6, 600 MHz).

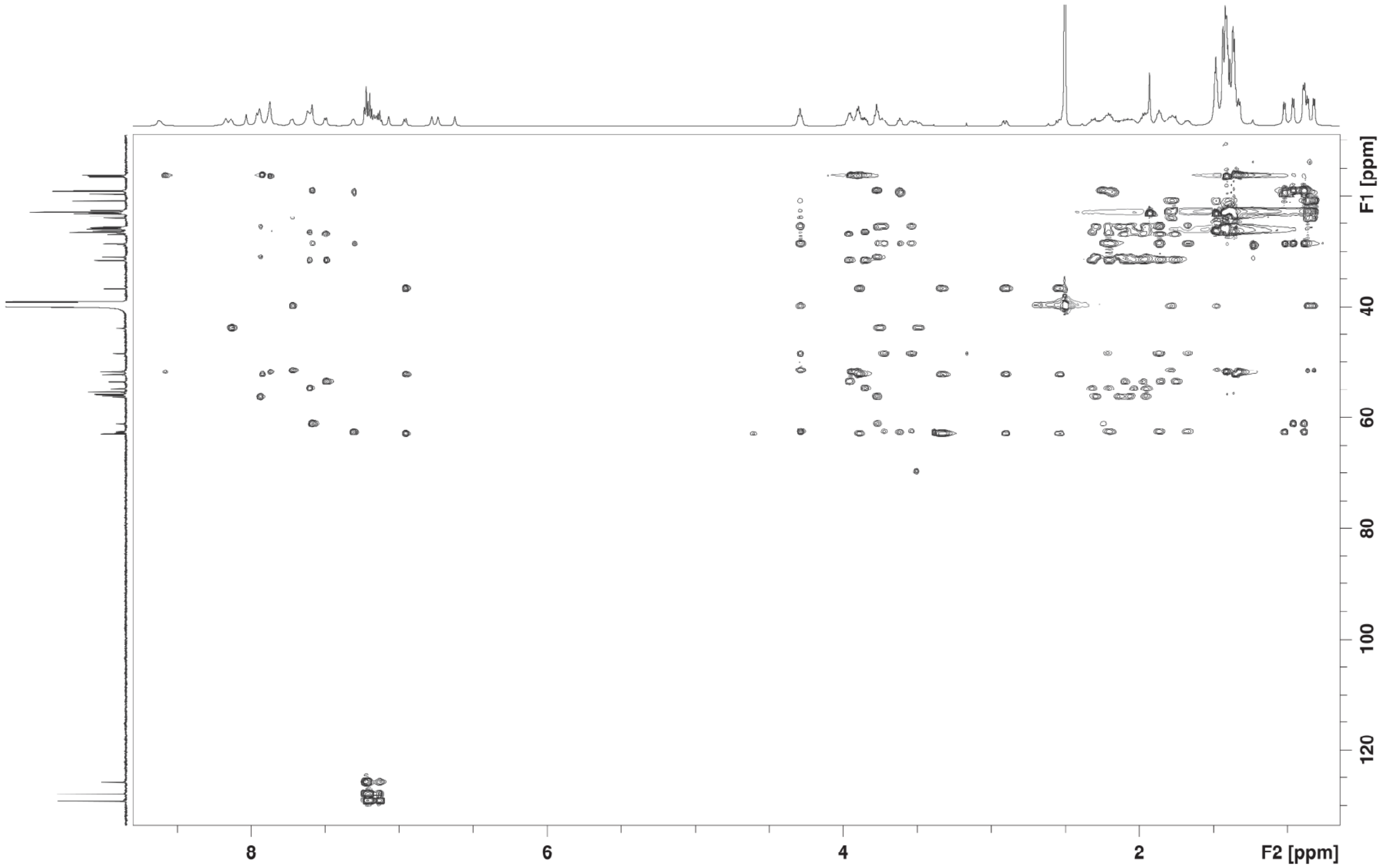

Figure S4. HSQC-TOCSY spectrum obtained for trichokonin VI (**1**) (DMSO-d_6).

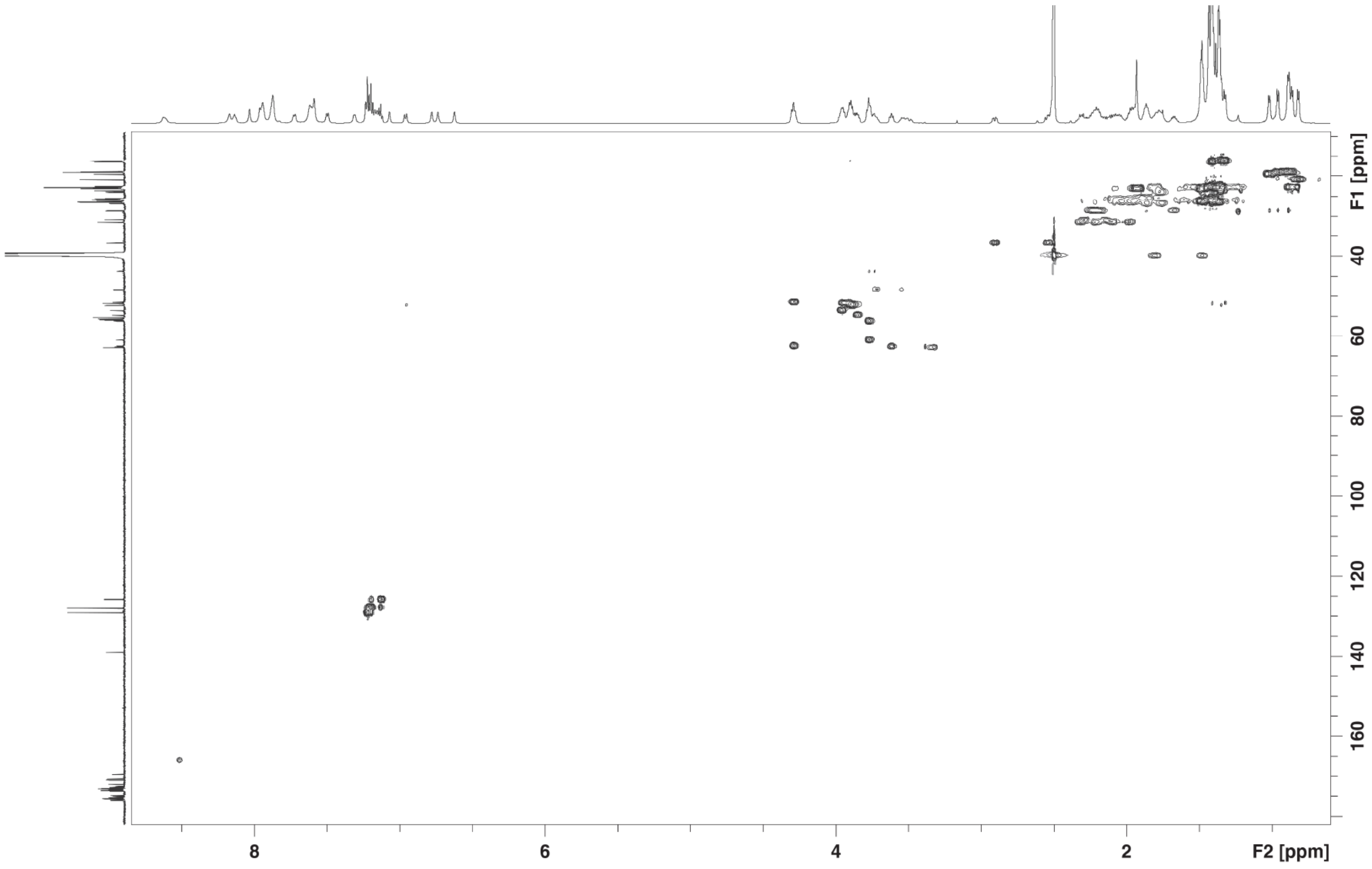

Figure S5. HSQC spectrum obtained for trichokonin VI (**1**) (DMSO-d_6).

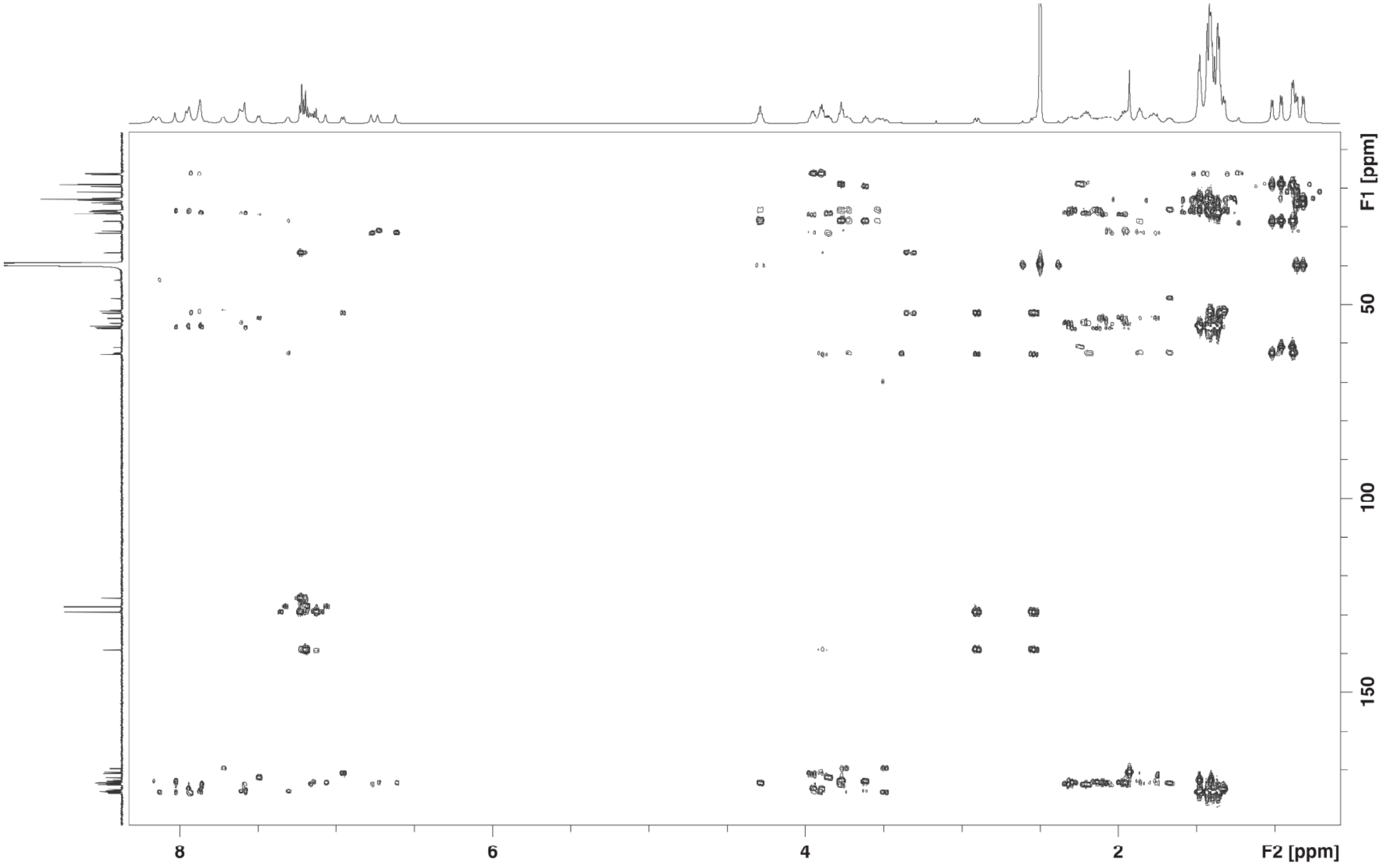

Figure S6. HMBC spectrum obtained for trichokonin VI (**1**) (DMSO-d_6, 600 MHz).

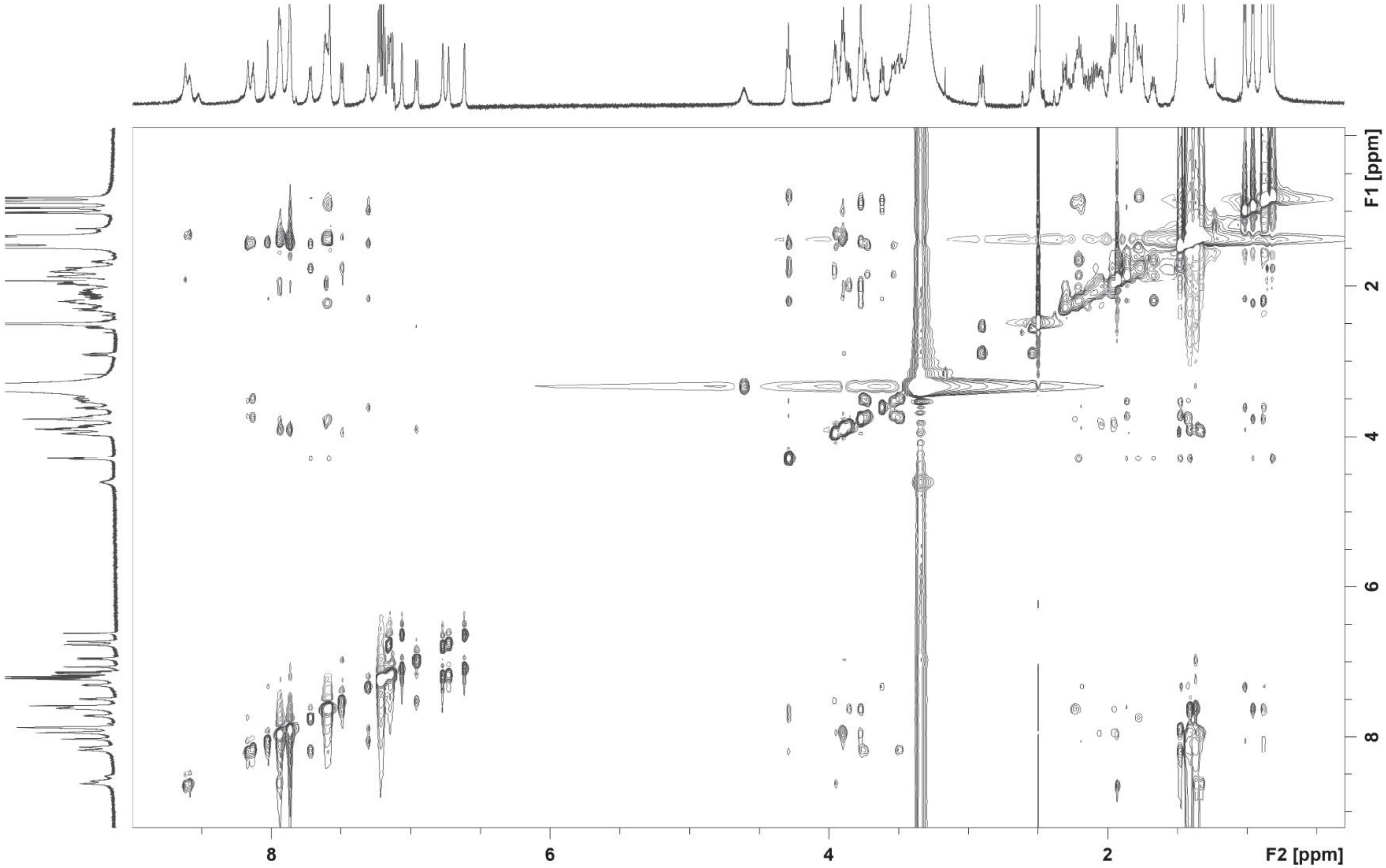

Figure S7. NOESY spectrum obtained for the trichokonin VI (**1**) (DMSO-d_6).

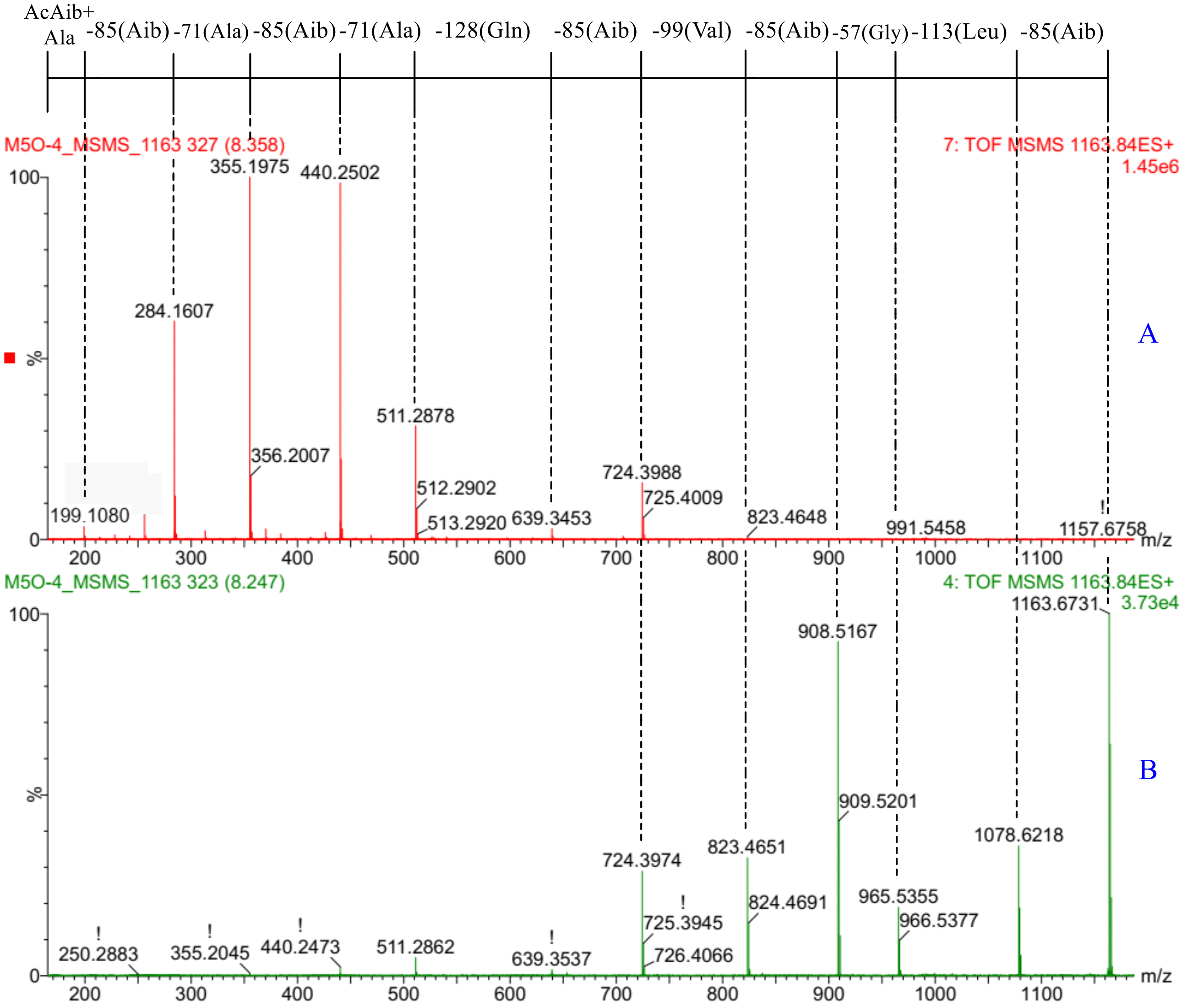

Figure S8. HRMS/MS spectrum obtained for the fragmentation of the ion *m/z* 1163.67 of trichokonin VI (**1**). (**A**) ESI+, 60 V; (**B**) ESI+, 30 V.

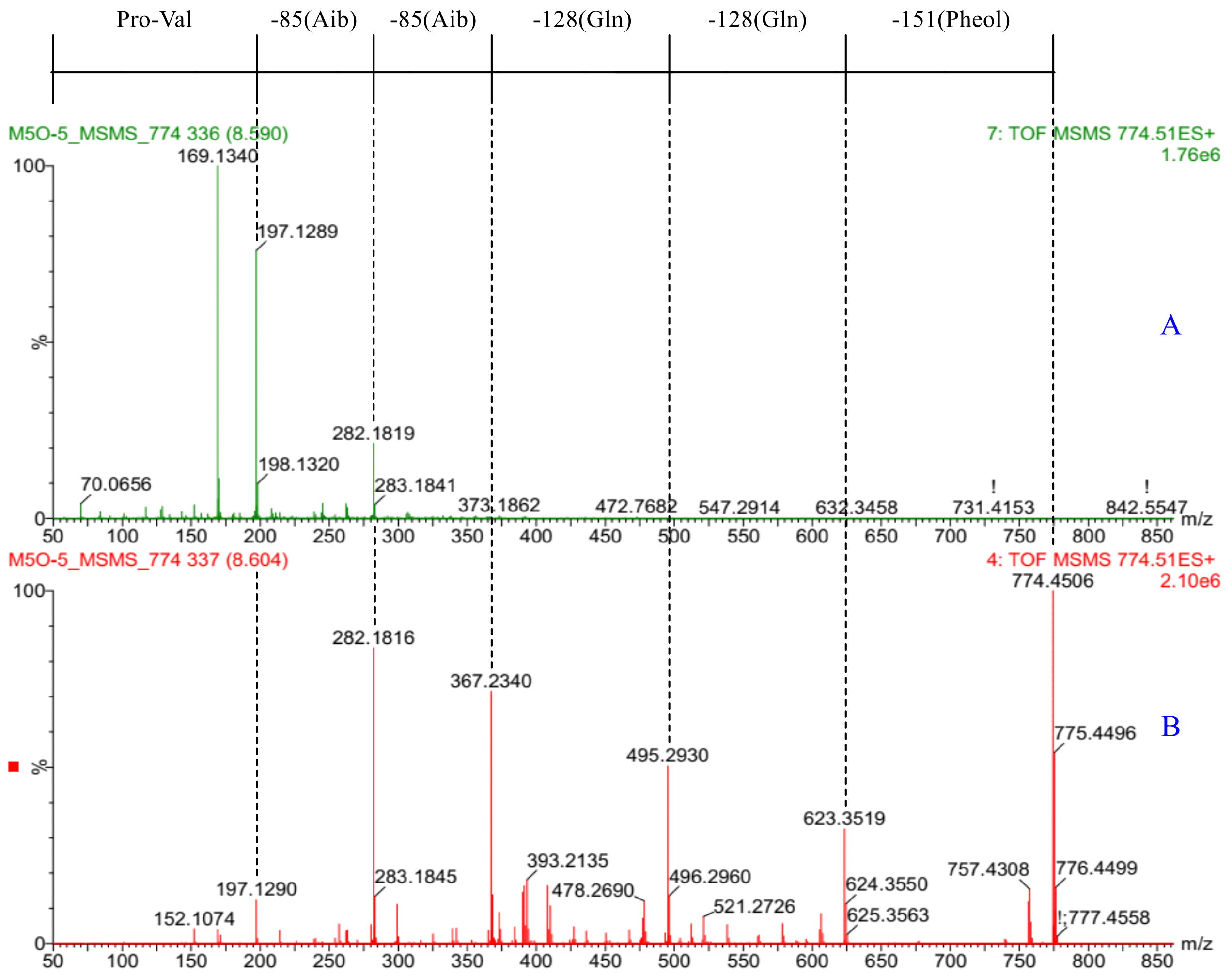

Figure S9. HRMS/MS spectrum obtained for the fragmentation of the ion *m/z* 774.45 of trichokonin VI (**1**). (**A**) ESI+, 60 V; (**B**) ESI+, 30 V.

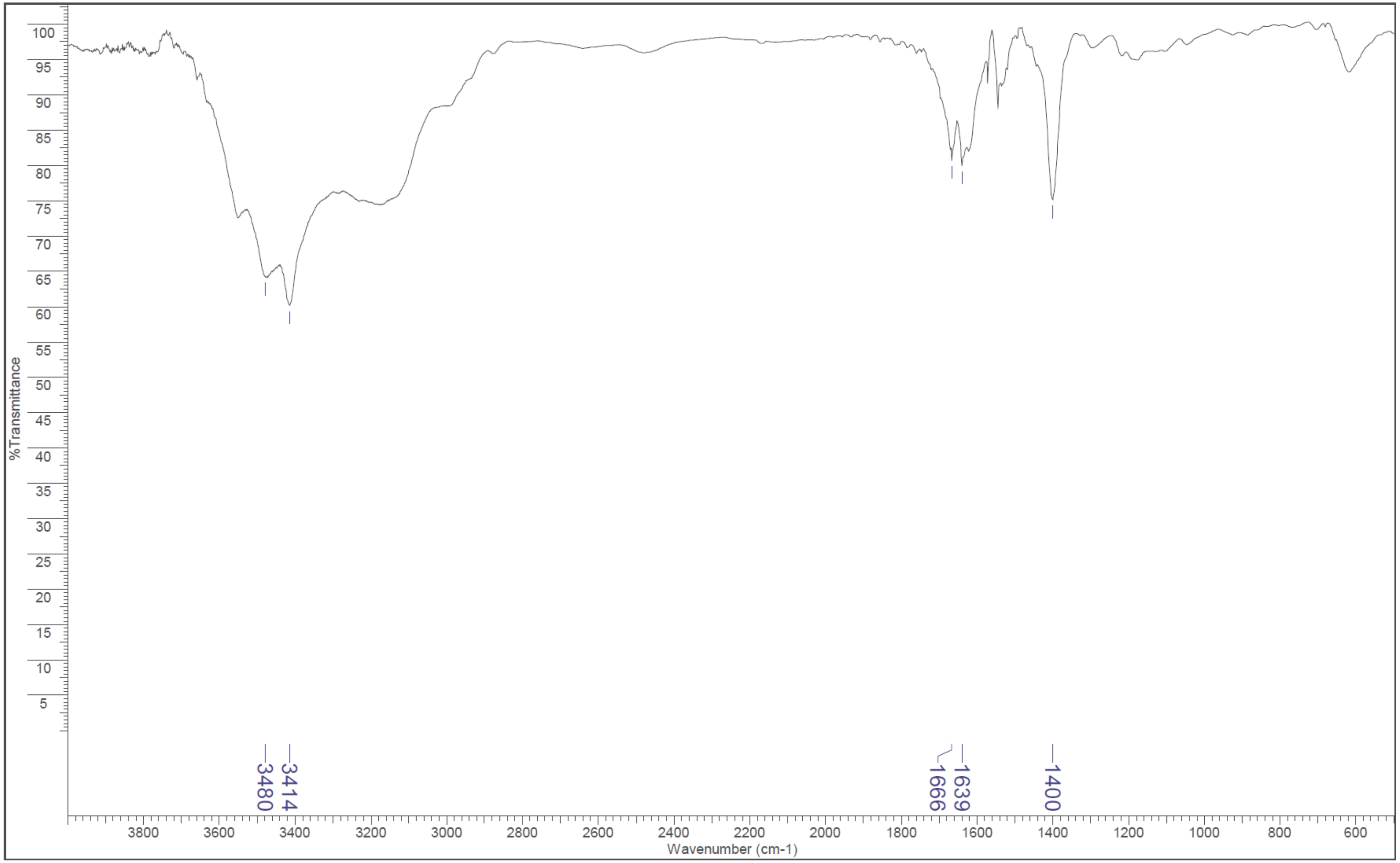

Figure S10. IR spectrum of the trichokonin VI (**1**) (cm^{-1}).

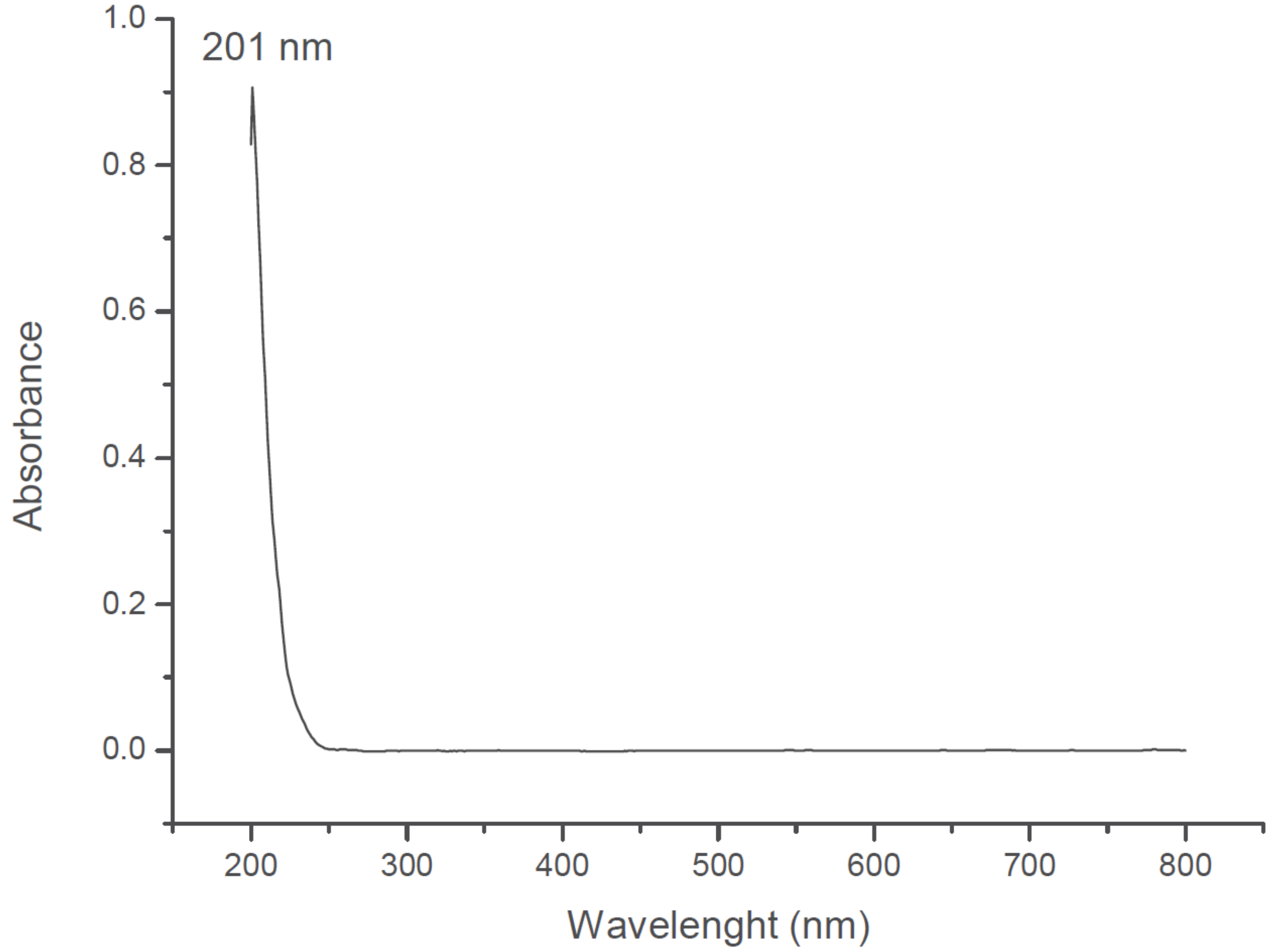

Figure S11. UV absorption spectrum of the trichokonin VI (**1**) (MeOH, c = 0.025 mg mL^{-1}).

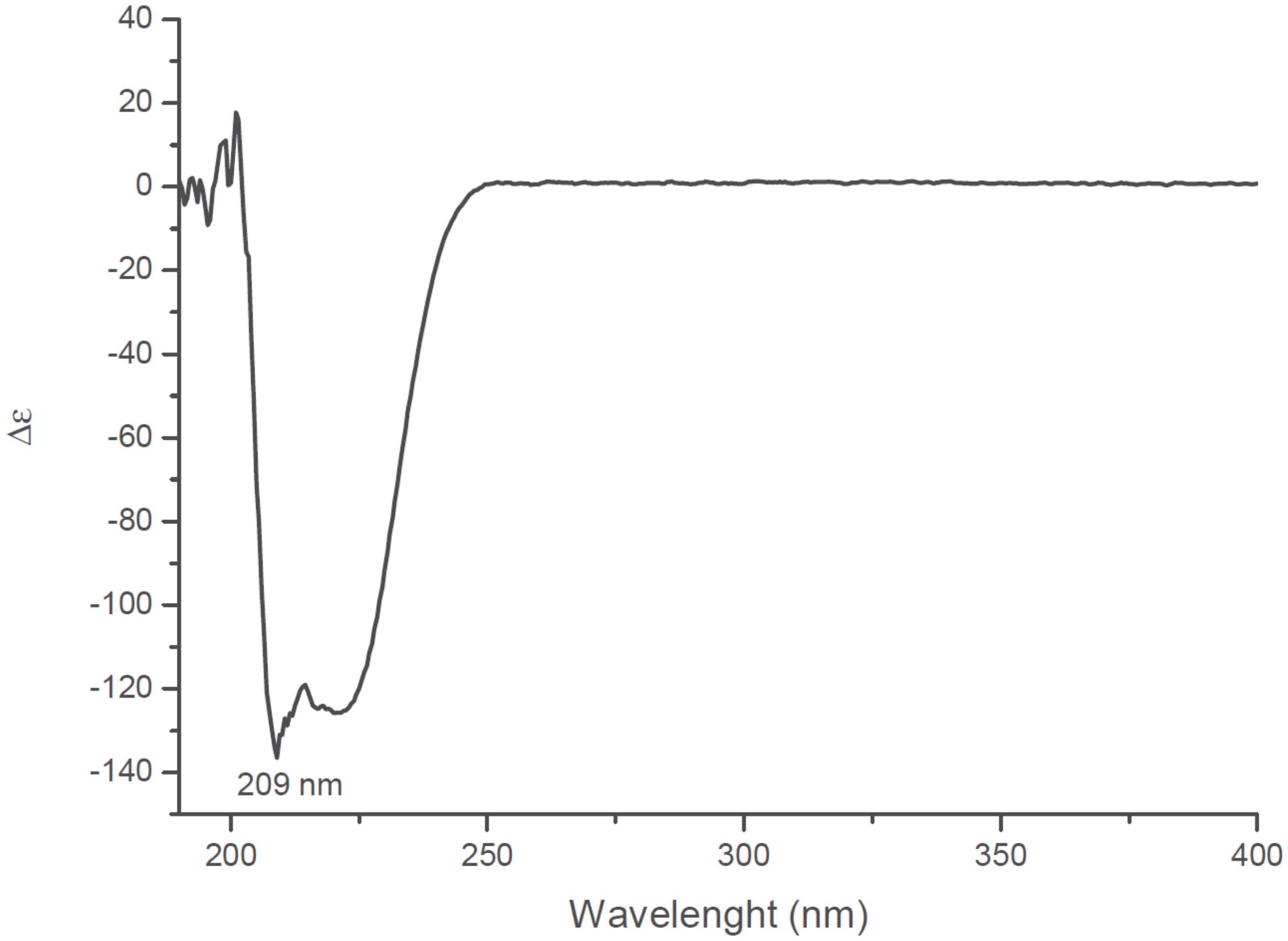

Figure S12. CD spectrum of trichokonin VI (**1**) (MeOH, c = 0.15 mg mL^{-1}).

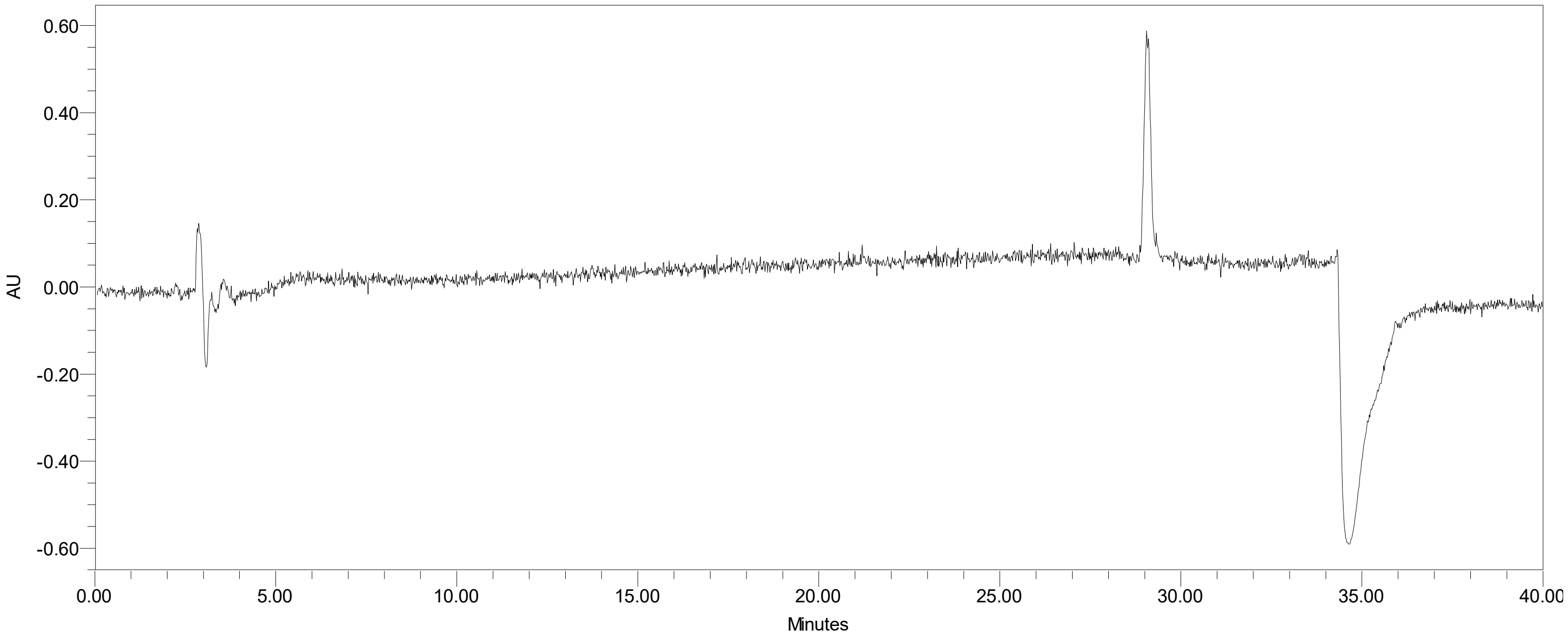

Figure S13. HPLC chromatogram of trichokonin VI (**1**) monitored at a wavelength of 210 nm, acquired using a C_{18} reversed phase column (Waters® X-terra, 250 × 4.6 mm, 5 µm) with a mobile flow rate of 1 mL min^{-1}.

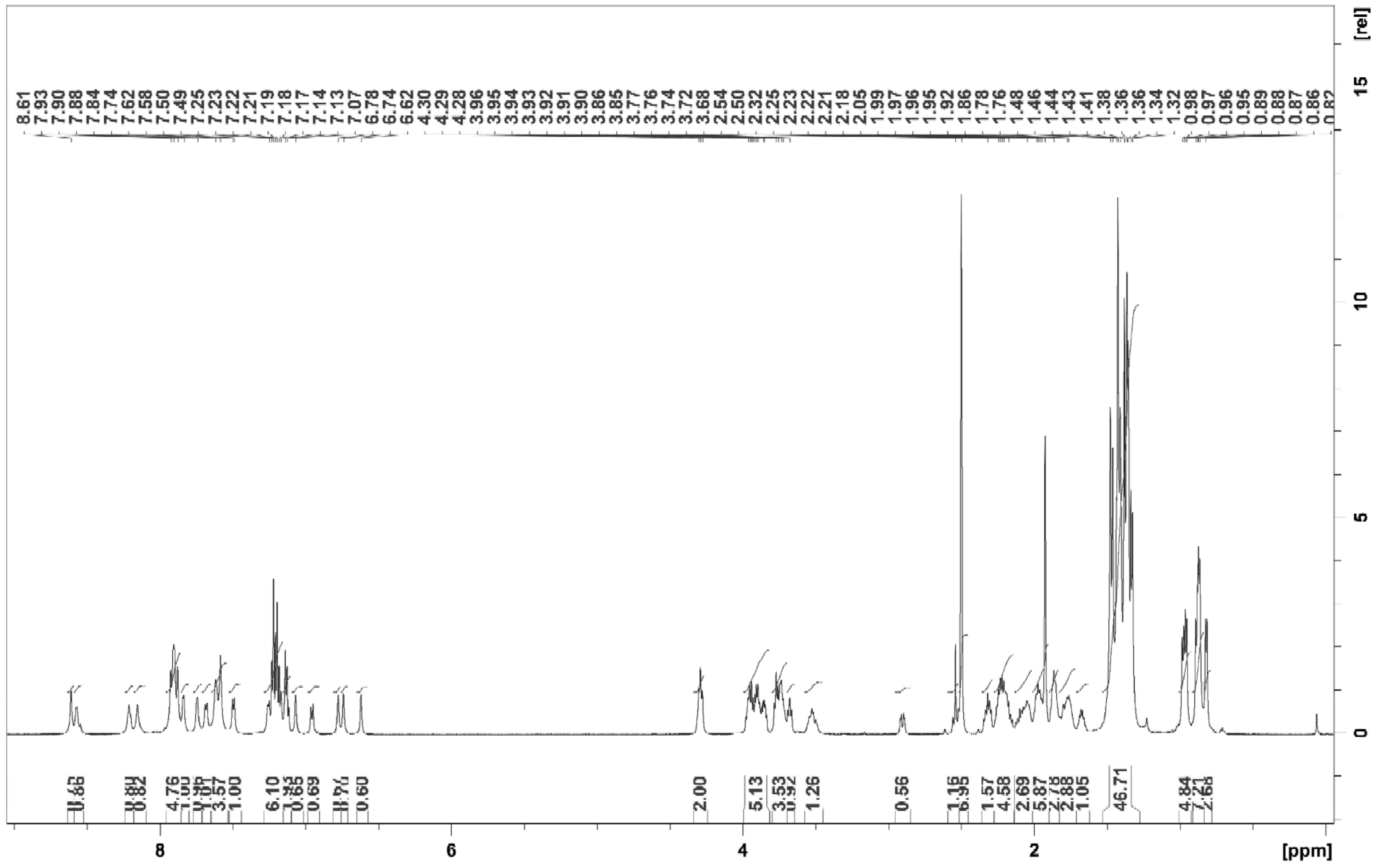

Figure S14. ^{1}H NMR spectrum of trichokonin VIII (**2**) (DMSO-d_6, 600 MHz).

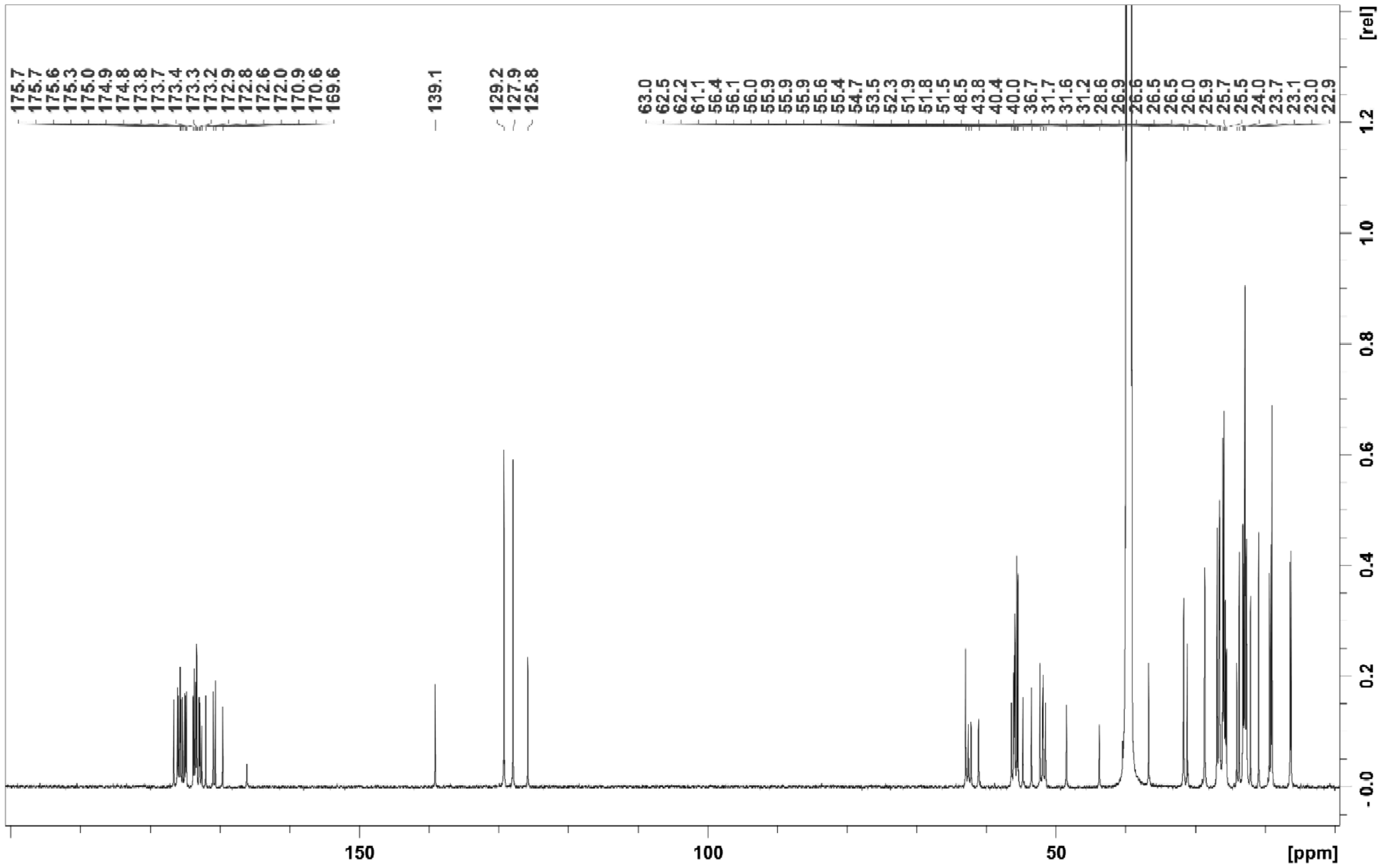

Figure S15. ^{13}C NMR spectrum of trichokonin VIII (**2**) (DMSO-d_6, 151 MHz).

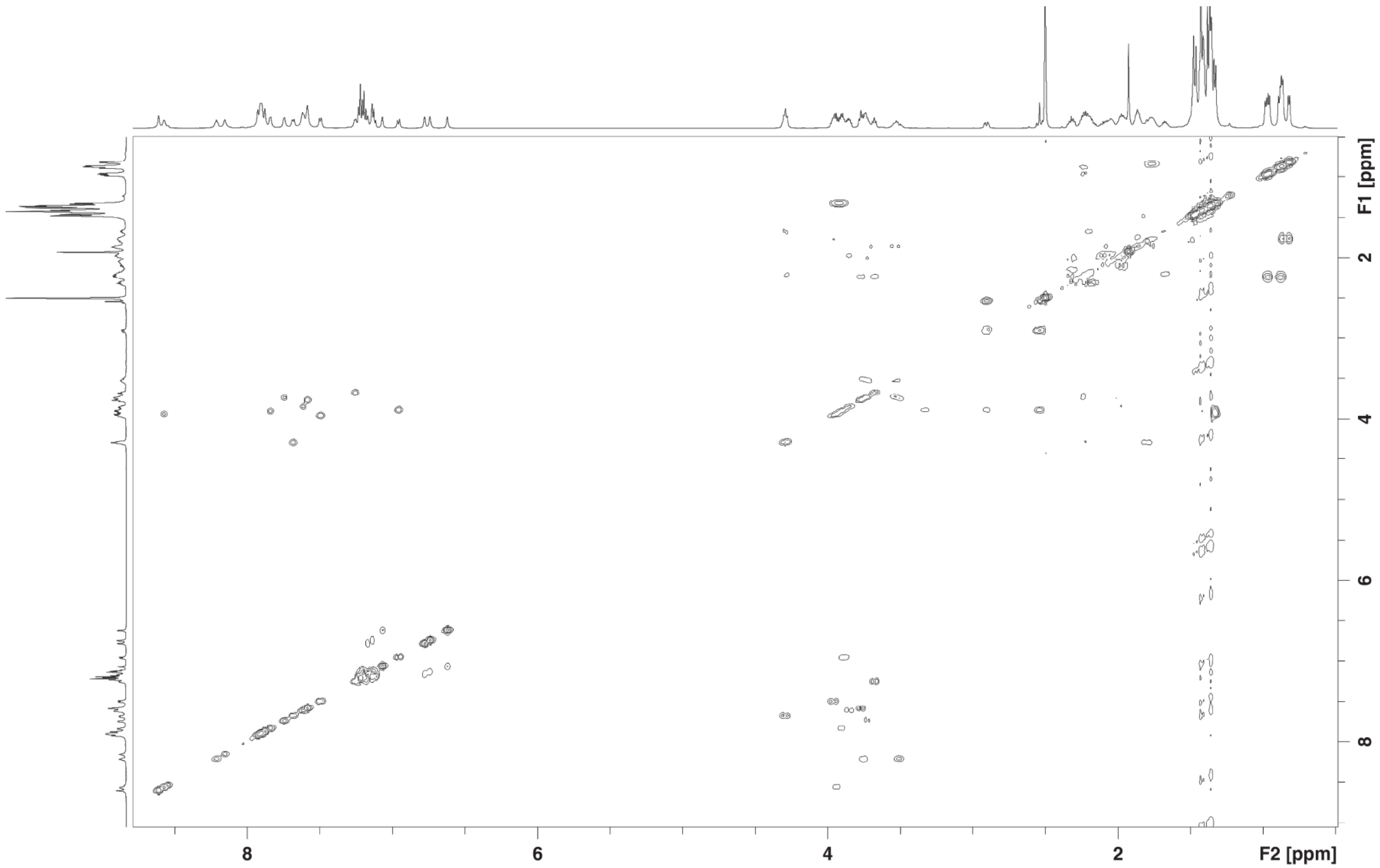

Figure S16. COSY spectrum obtained for trichokonin VIII (**2**) (DMSO-d_6, 600 MHz).

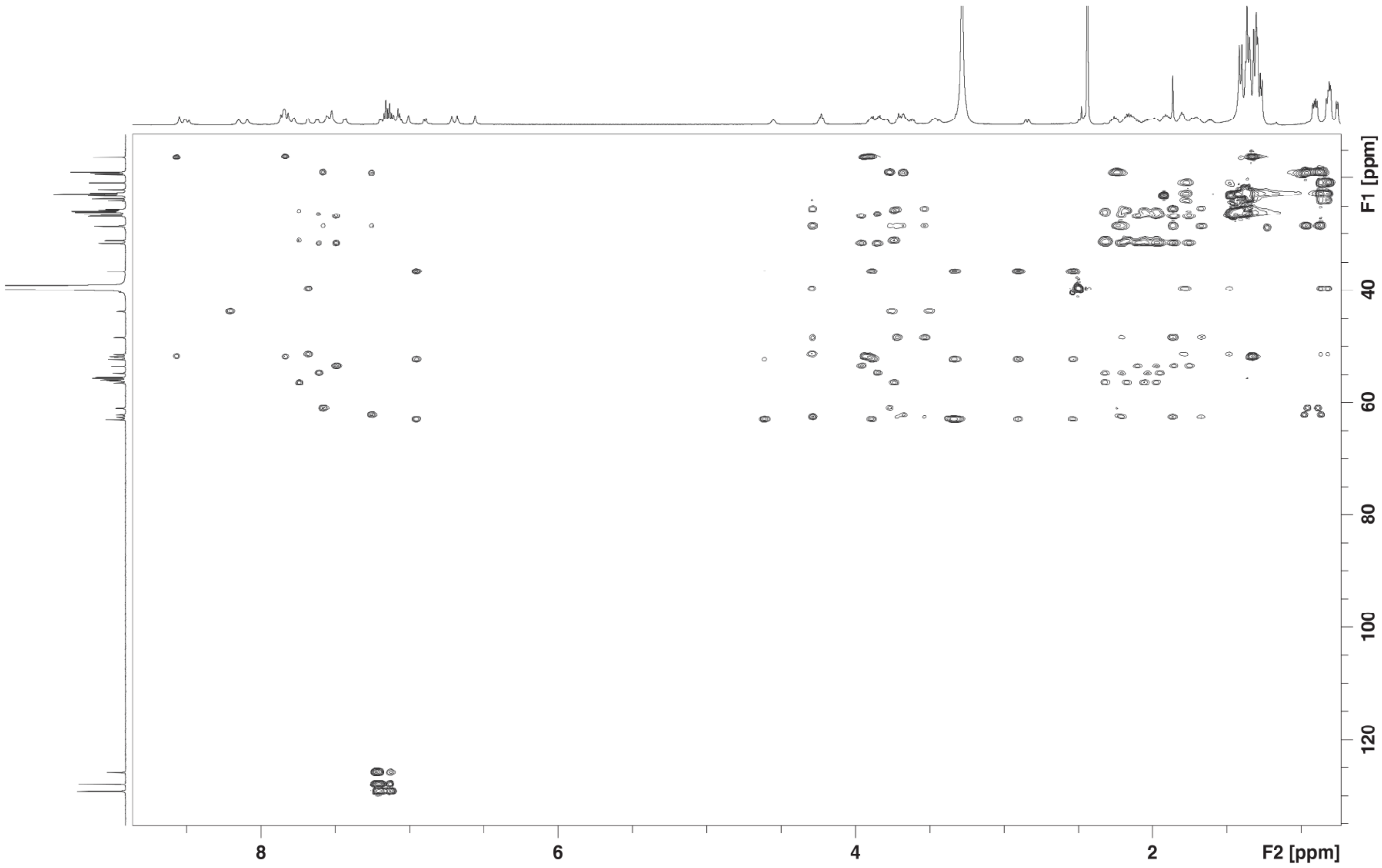

Figure S17. HSQC-TOCSY spectrum obtained for trichokonin VIII (**2**) (DMSO-d_6).

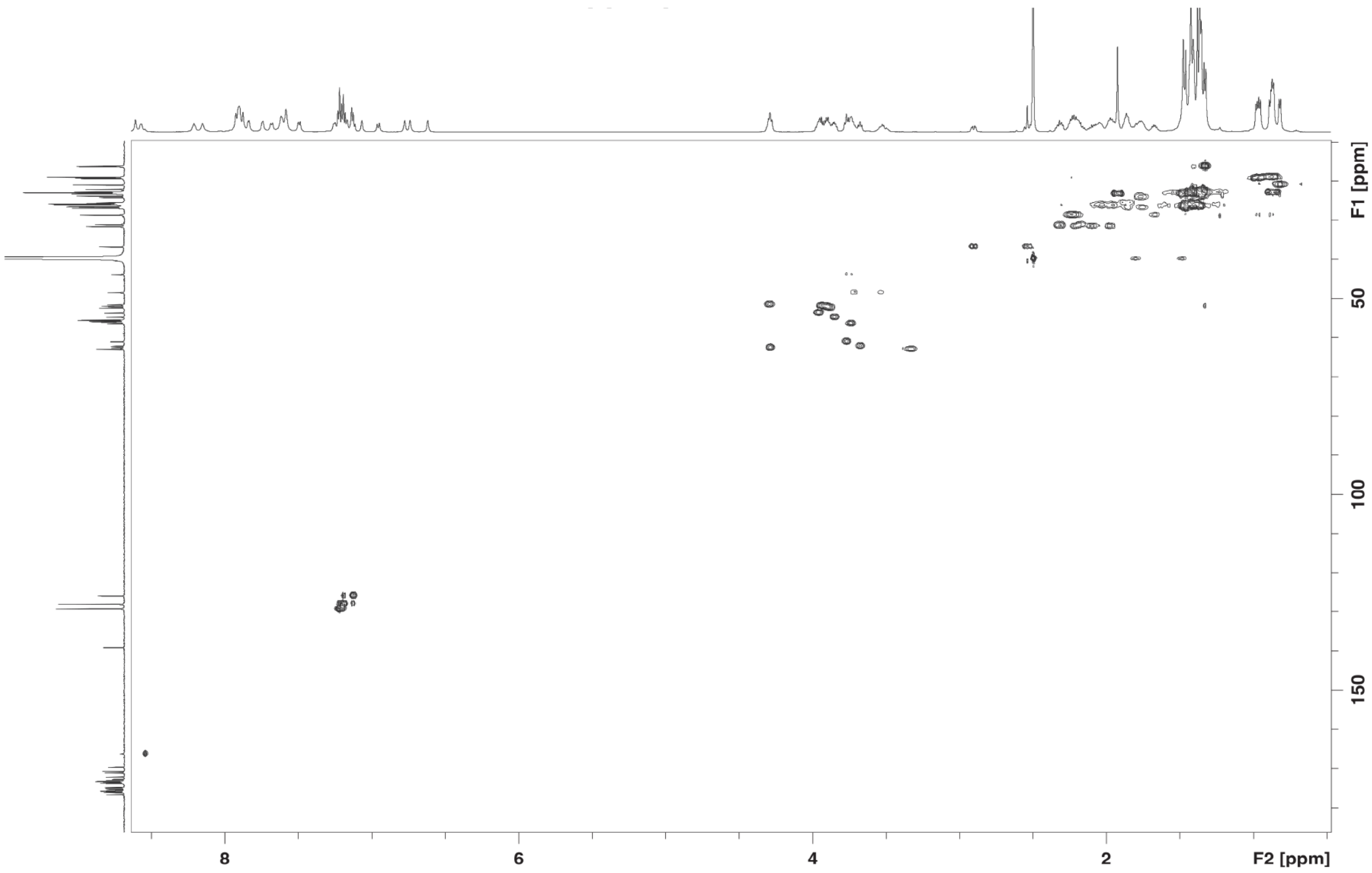

Figure S18. HSQC spectrum obtained for trichokonin VIII (**2**) (DMSO-d_6).

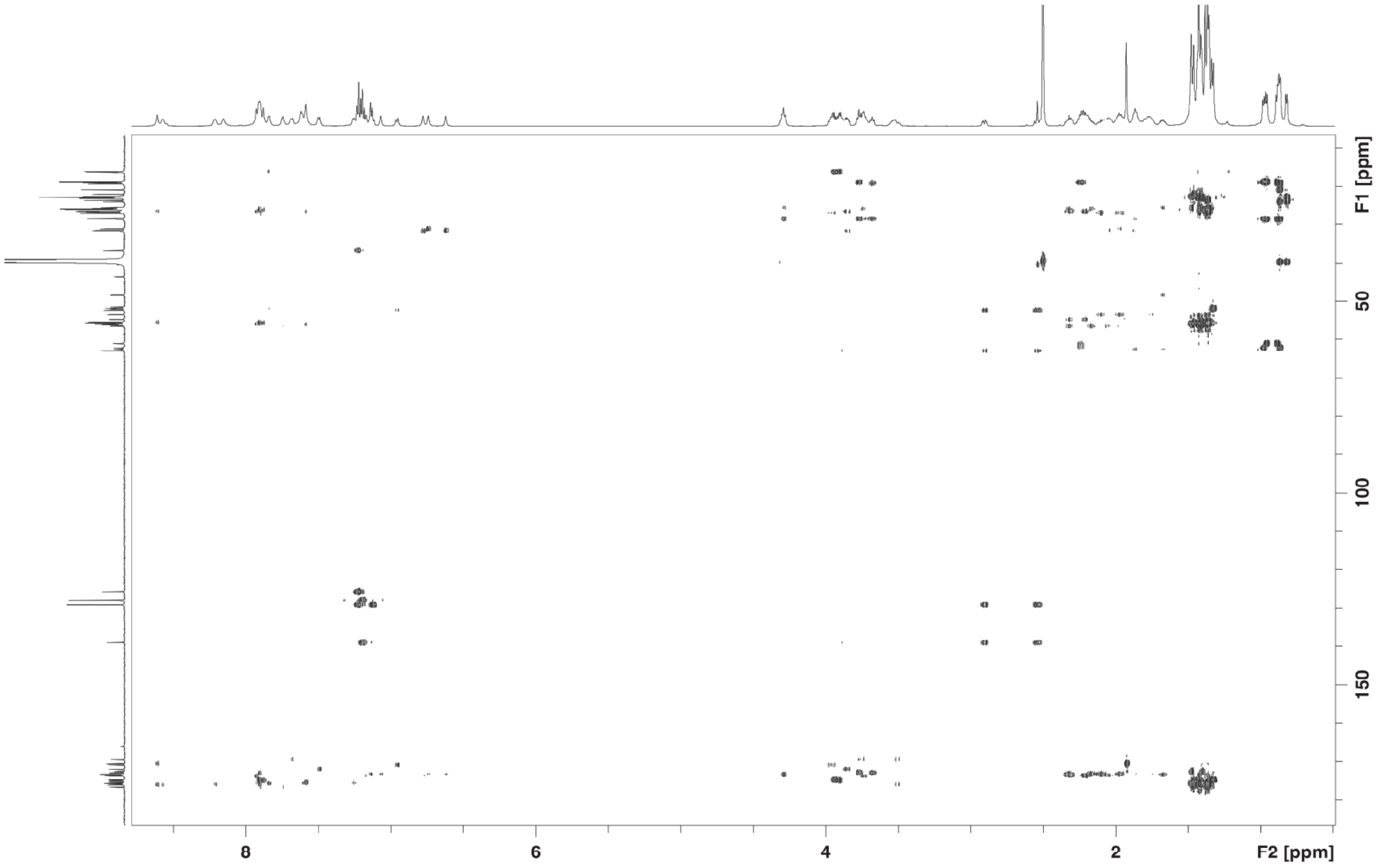

Figure S19. HMBC spectrum obtained for trichokonin VIII (**2**) (DMSO-d_6).

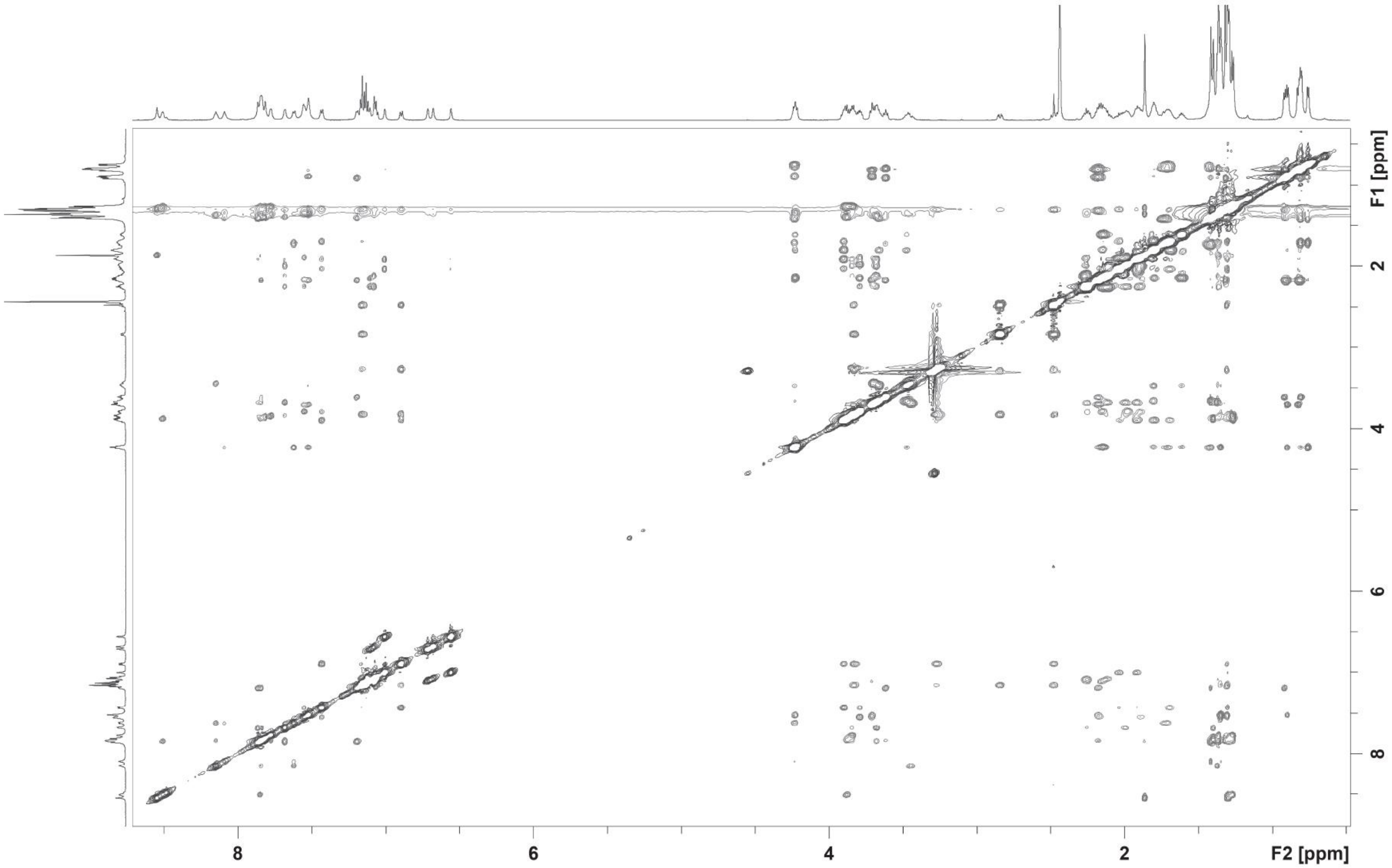

Figure S20. ROESY spectrum obtained for the trichokonin VIII (**2**) (DMSO-d_6).

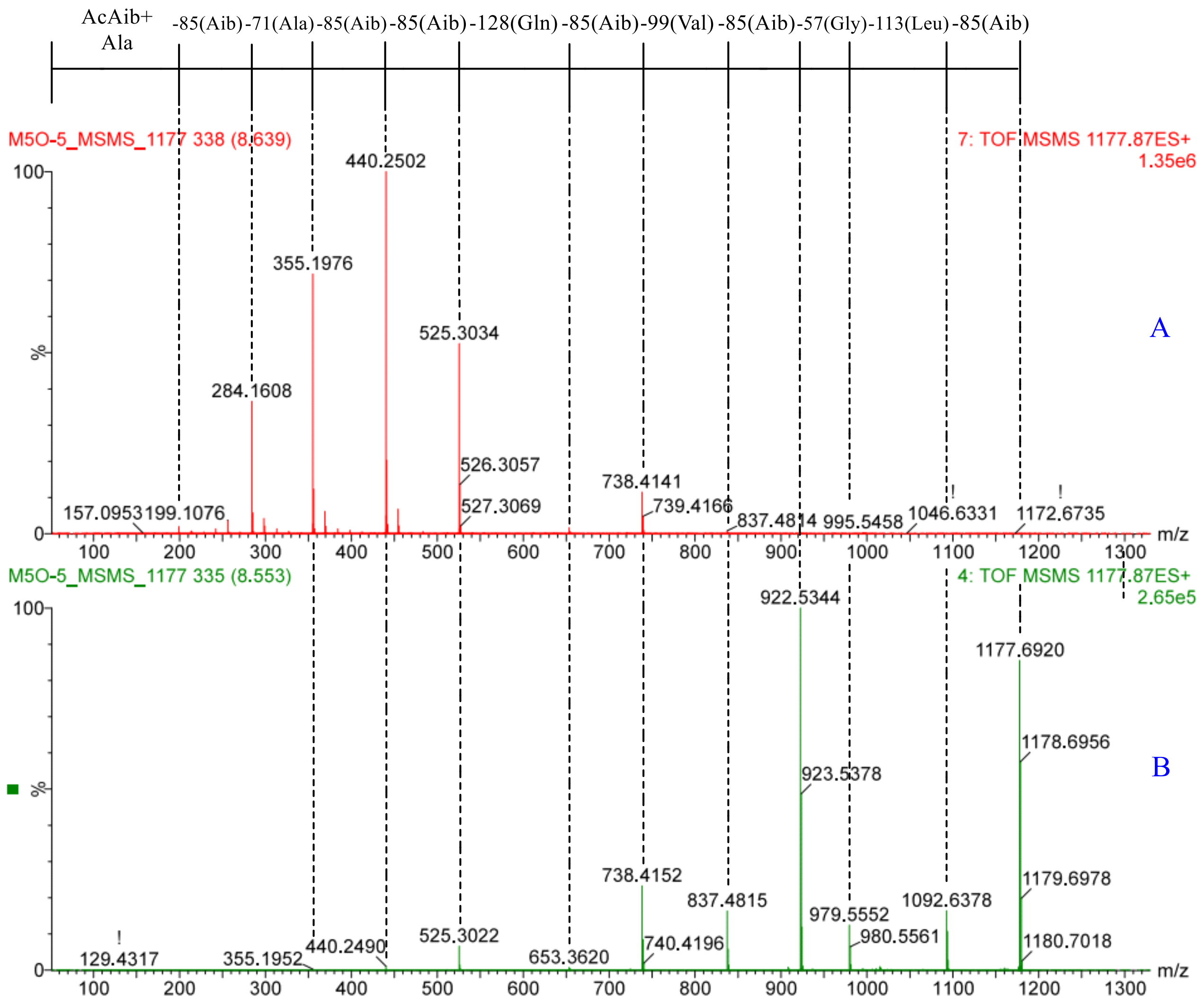

Figure S21. HRMS/MS spectrum obtained for the fragmentation of the ion *m/z* 1177.69 of trichokonin VIII (**2**). (**A**) ESI+, 60 V; (**B**) ESI+, 30 V.

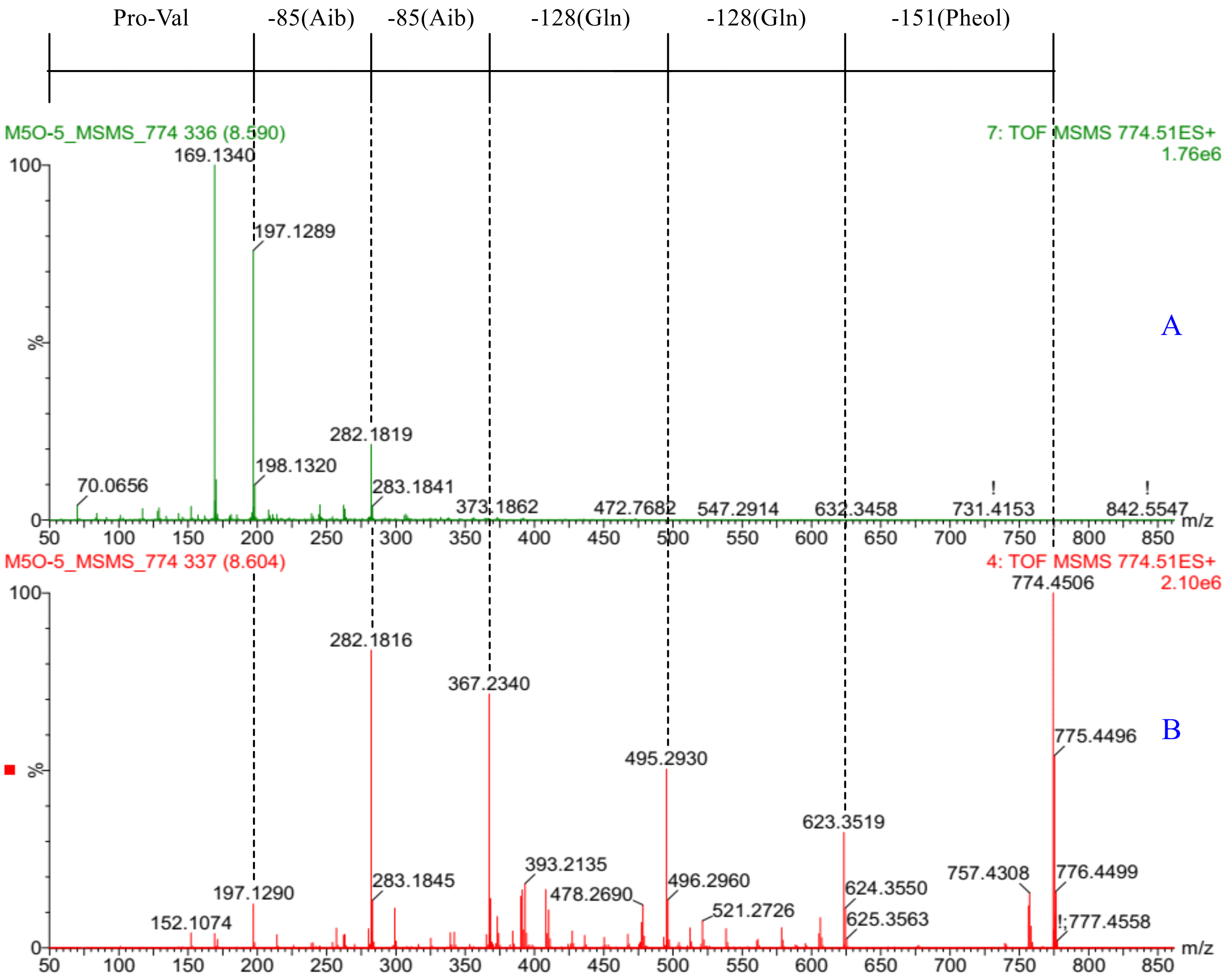

Figure S22. HRMS/MS spectrum obtained for the fragmentation of the ion *m/z* 774.45 of trichokonin VIII (**2**). (**A**) ESI+, 60 V; (**B**) ESI+, 30 V.

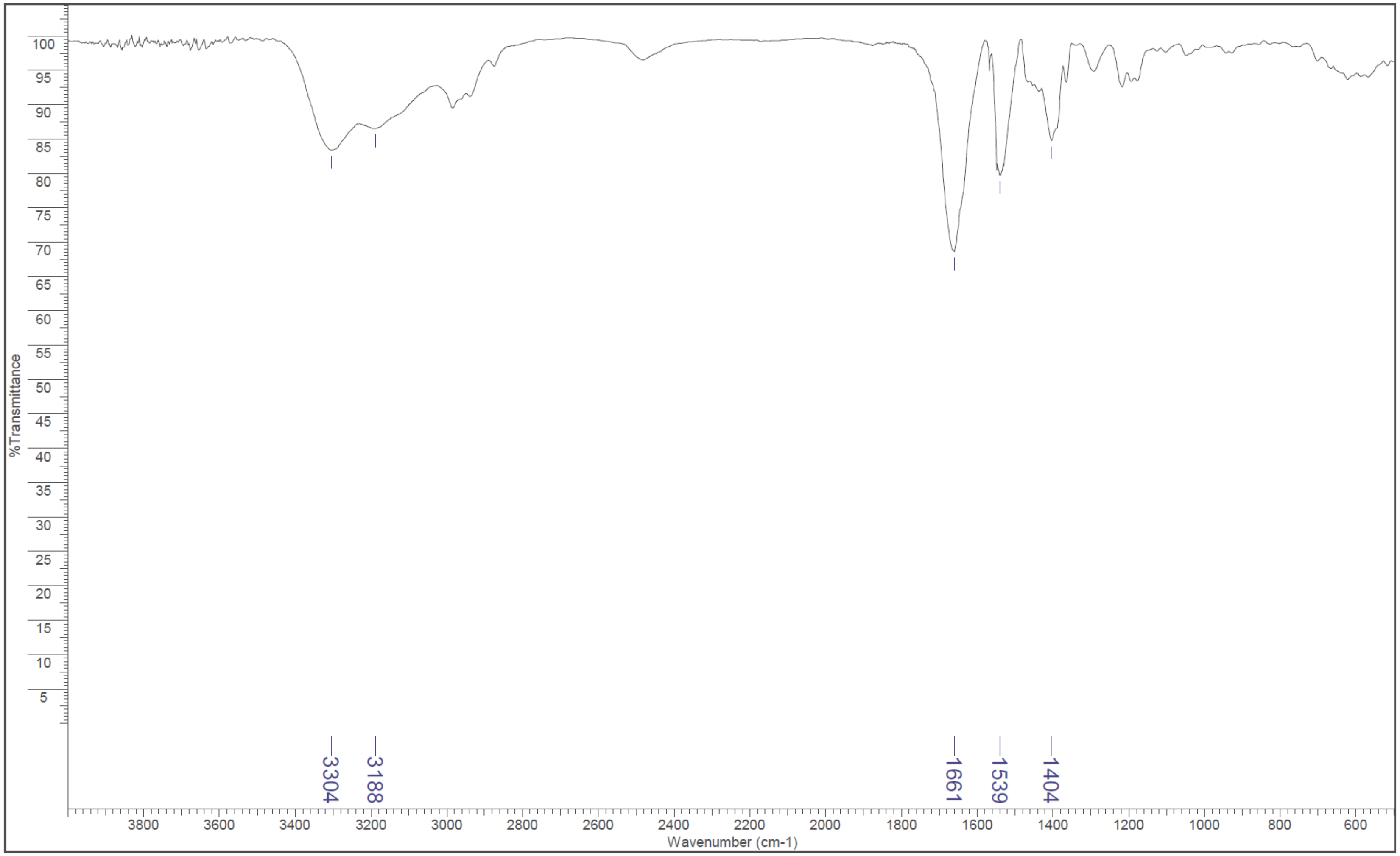

Figure S23. IR spectrum of the trichokonin VIII (**2**) (cm^{-1}).

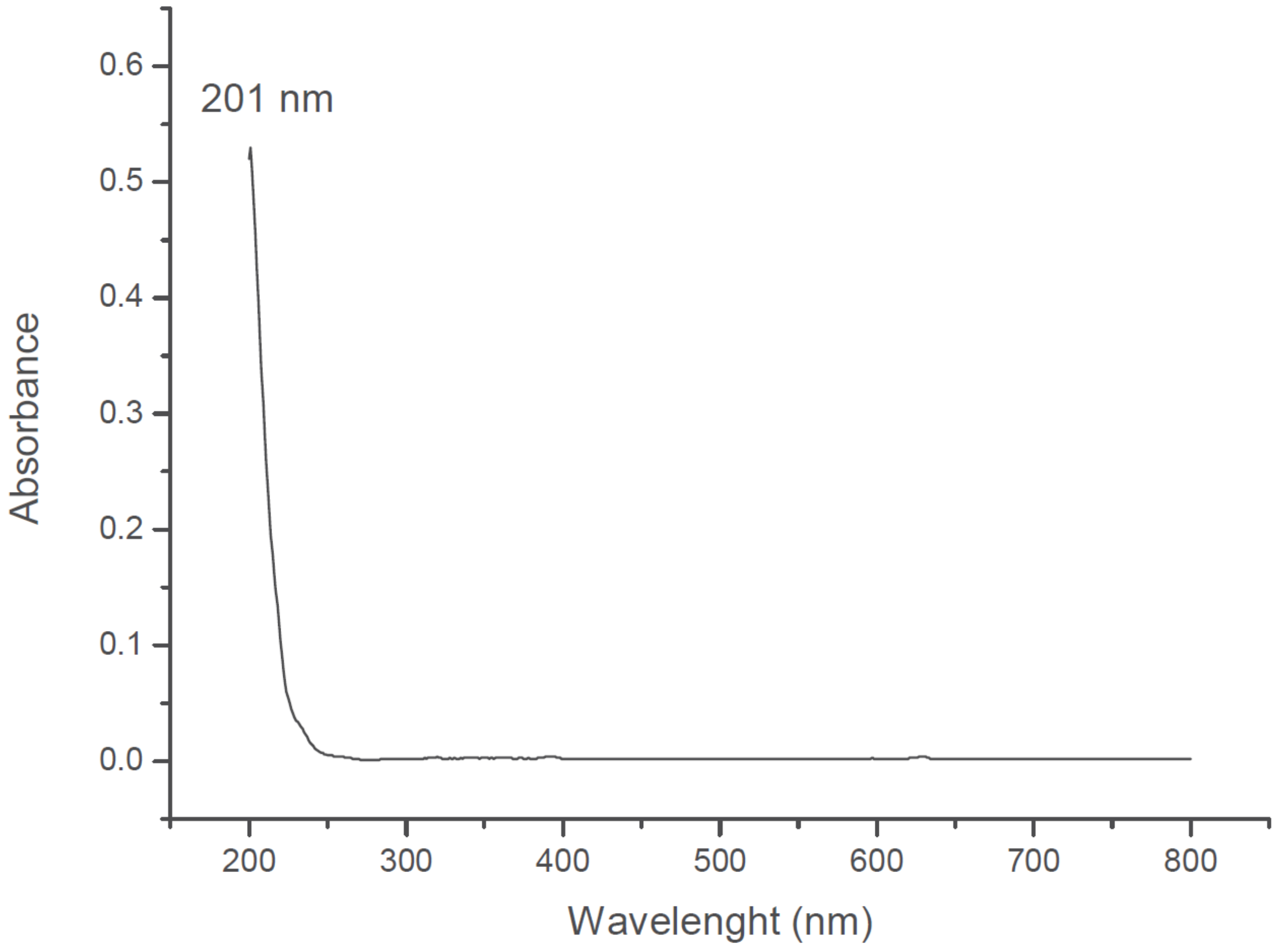

Figure S24. UV absorption spectrum of the trichokonin VIII (**2**) (MeOH, c = 0.025 mg mL^{-1}).

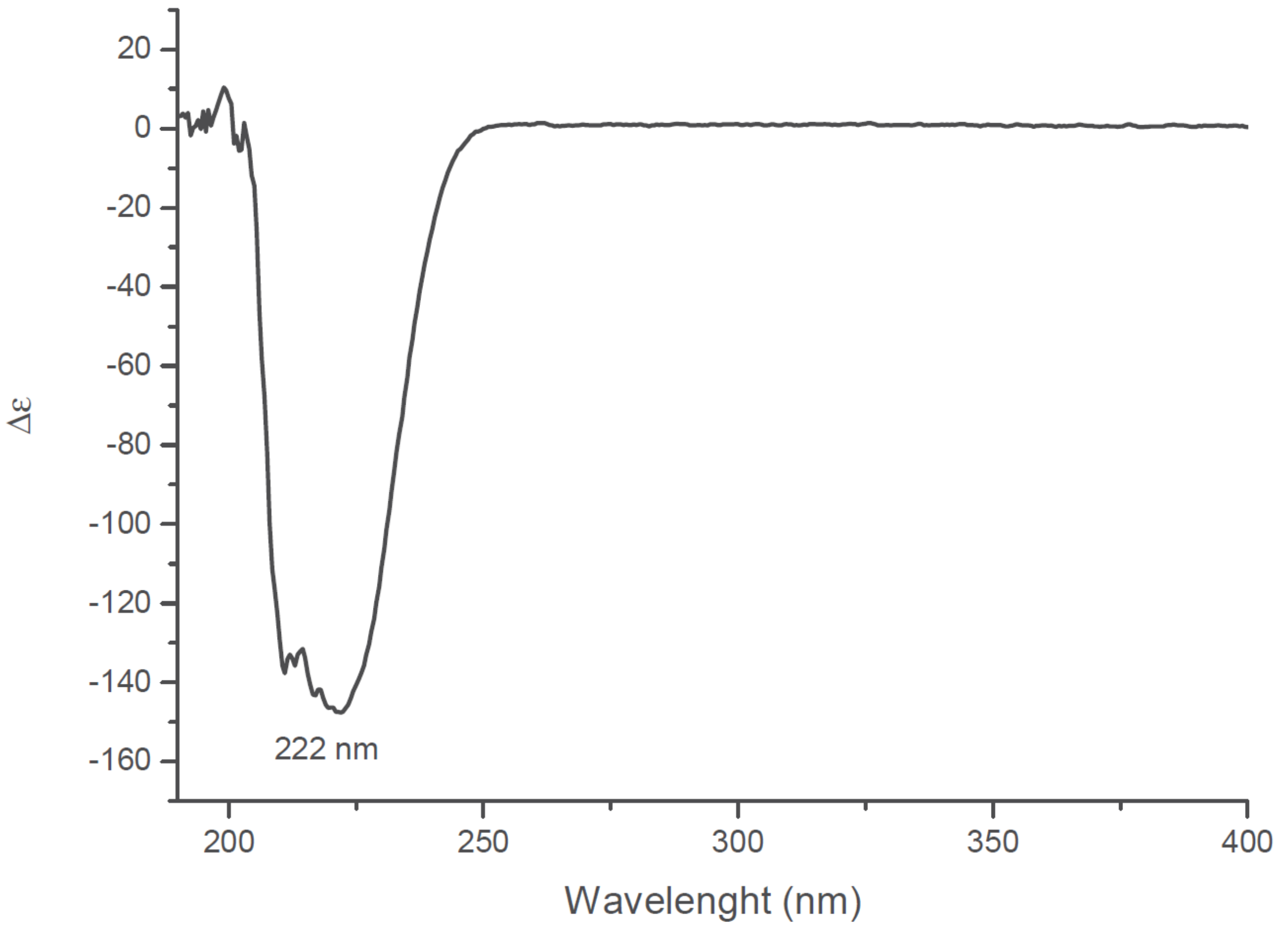

Figure S25. CD spectrum of trichokonin VIII (**2**) (MeOH, c = 0.15 mg mL^{-1}).

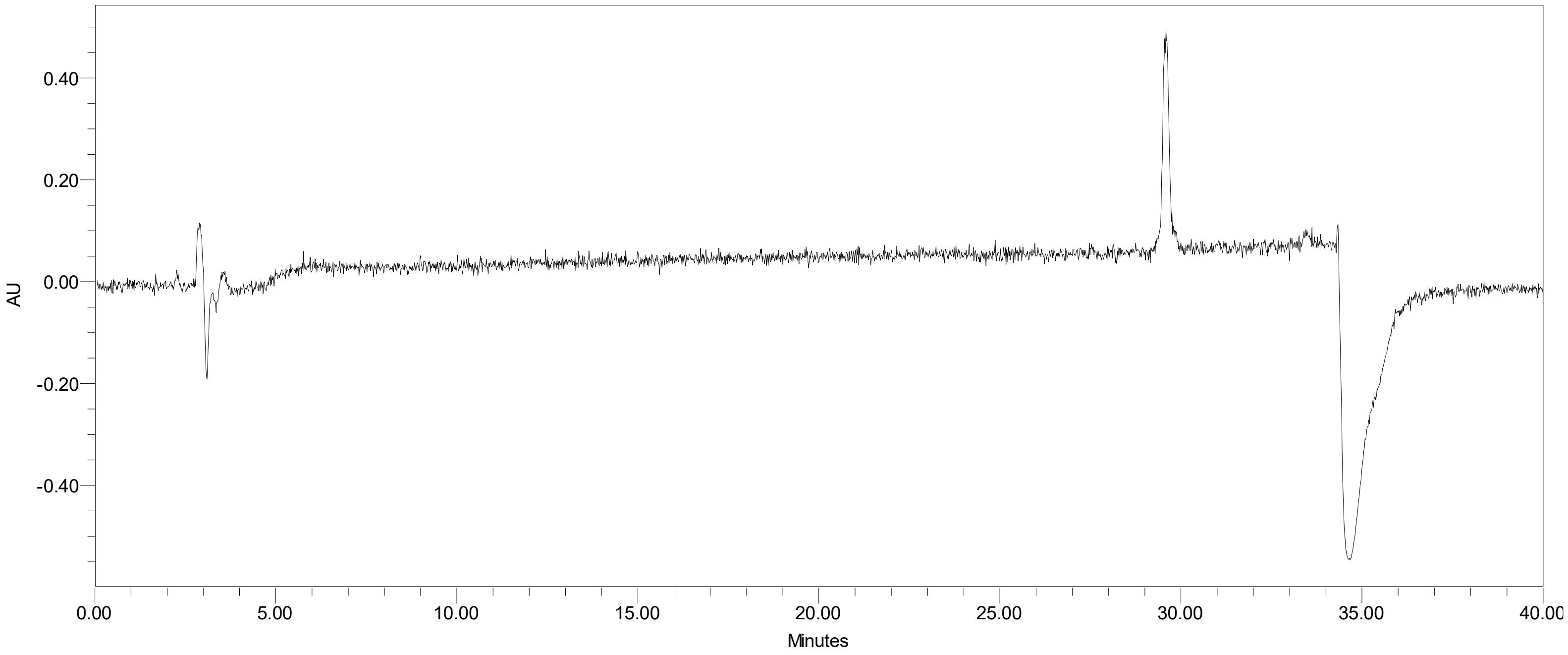

Figure S26. HPLC chromatogram of trichokonin VIII (**2**) monitored at a wavelength of 210 nm, acquired using a C_{18} reversed phase column (Waters® X-terra, 250 × 4.6 mm, 5 µm) with a mobile flow rate of 1 mL min^{-1}.

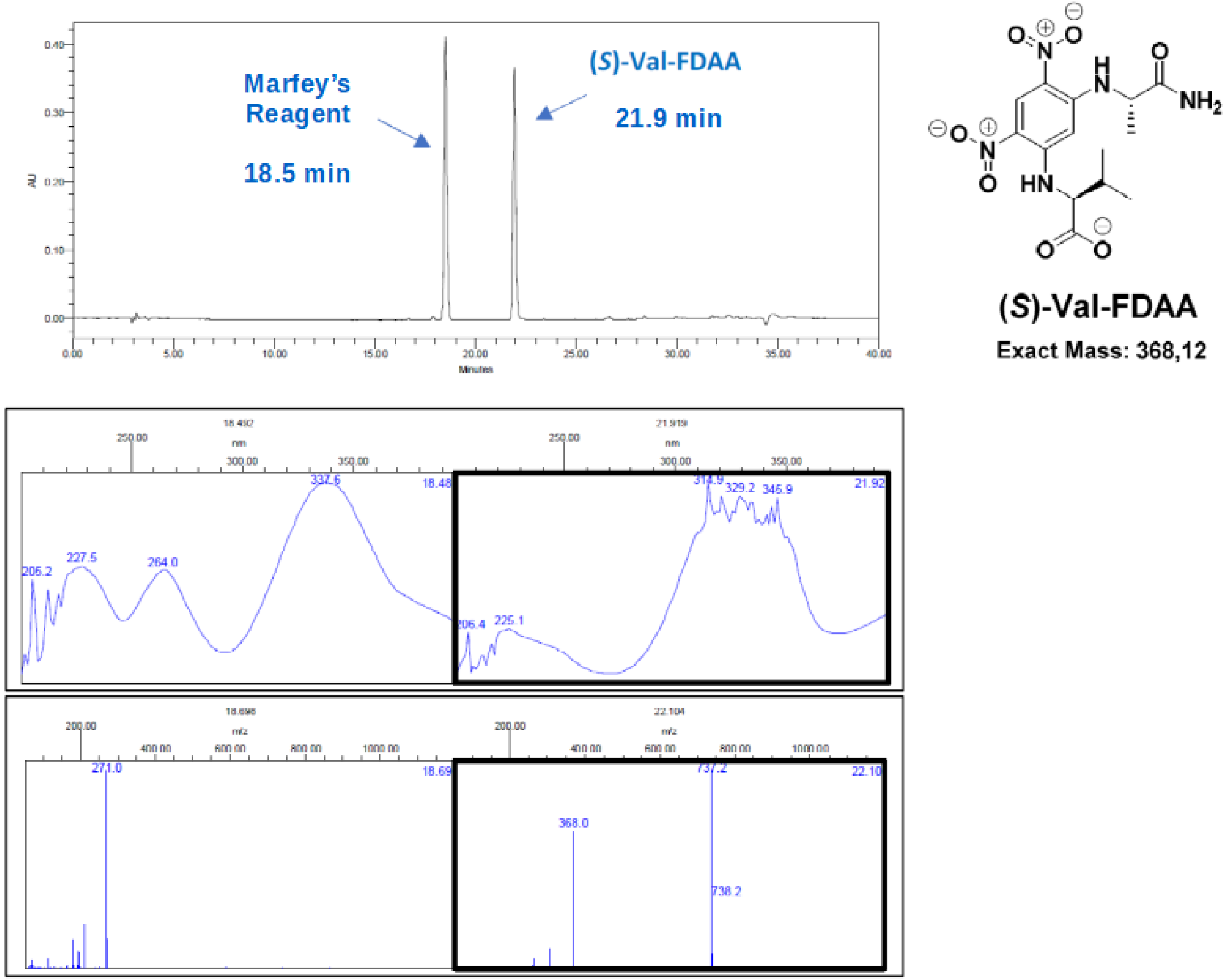

Figure S27. Chromatogram of Marfey reaction with Val (280 nm), UV and MS spectrum (ESI⁻).

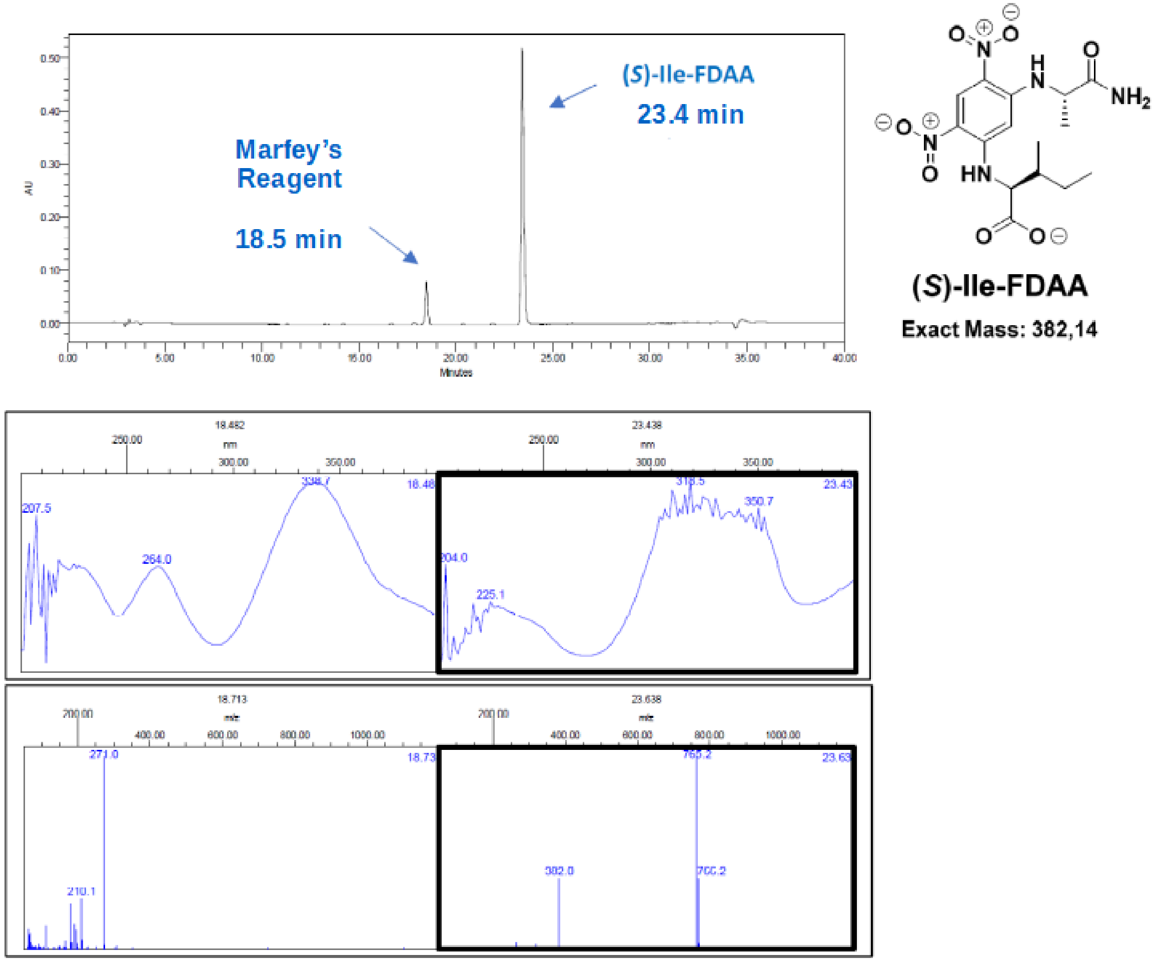

Figure S28. Chromatogram of Marfey reaction with Ile (280 nm), UV and MS spectrum (ESI^{-}).

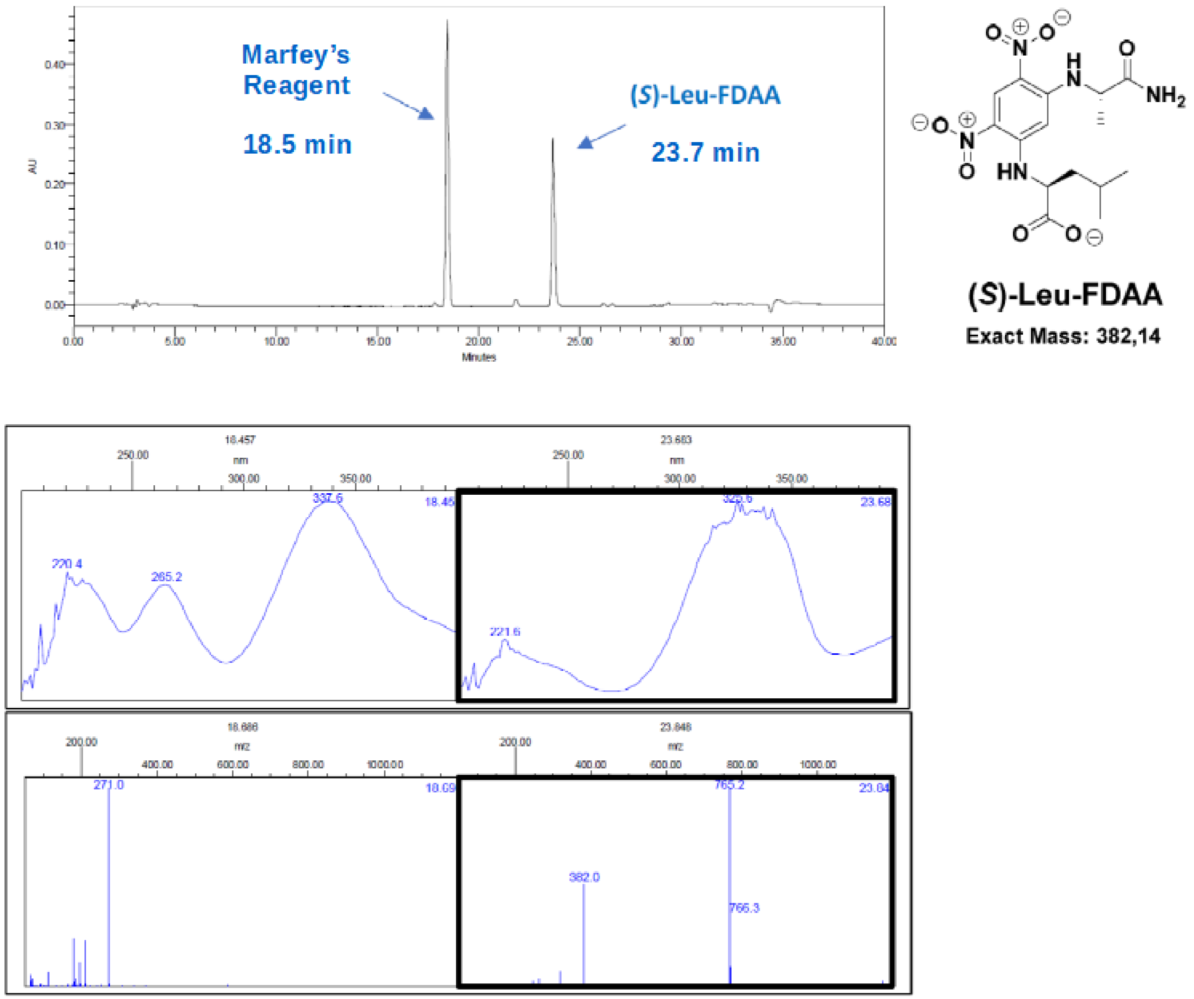

Figure S29. Chromatogram of Marfey reaction with Leu (280 nm), UV and MS spectrum (ESI⁻).

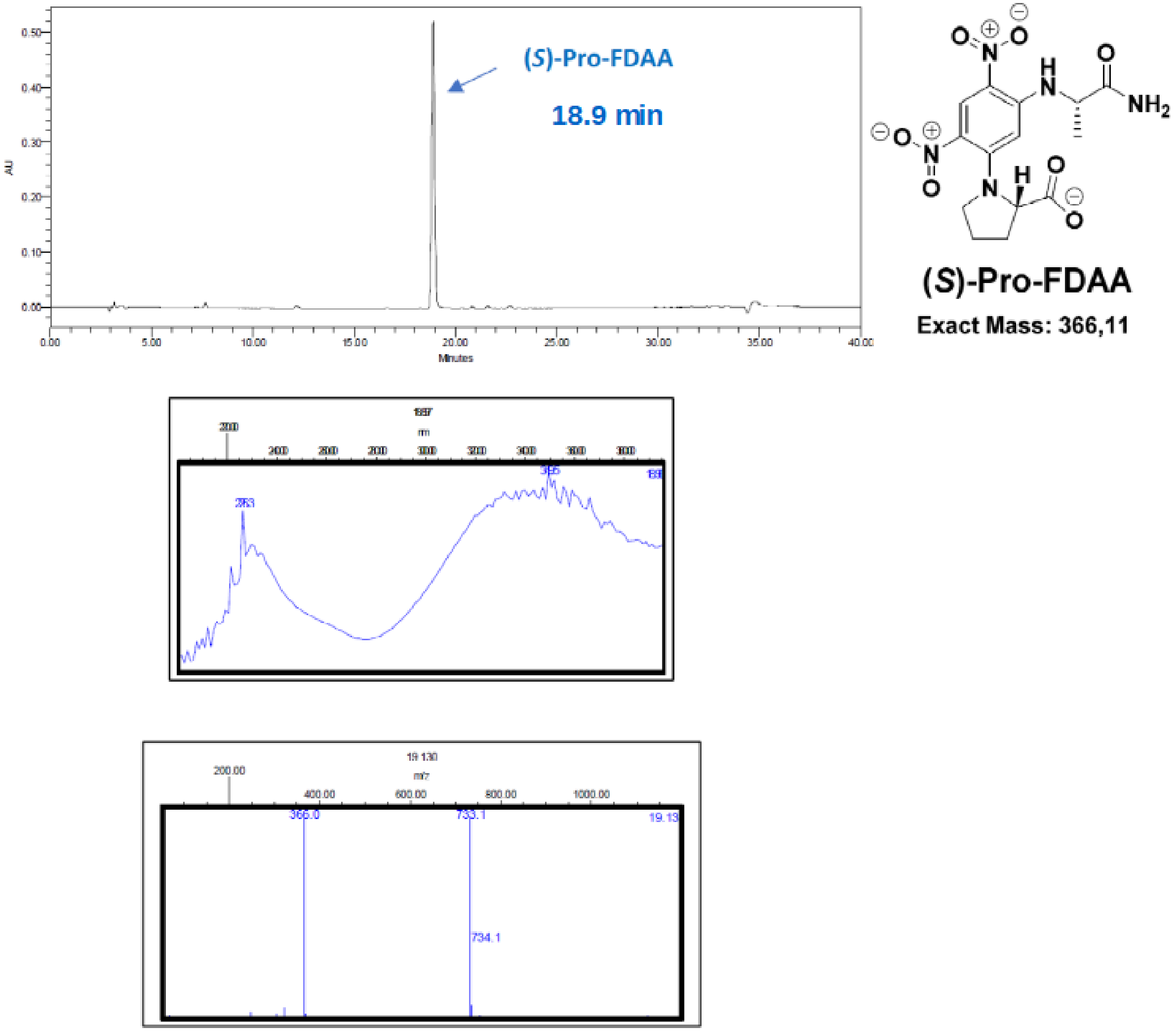

Figure S30. Chromatogram of Marfey reaction with Pro (280 nm),UV and MS spectrum (ESI⁻).

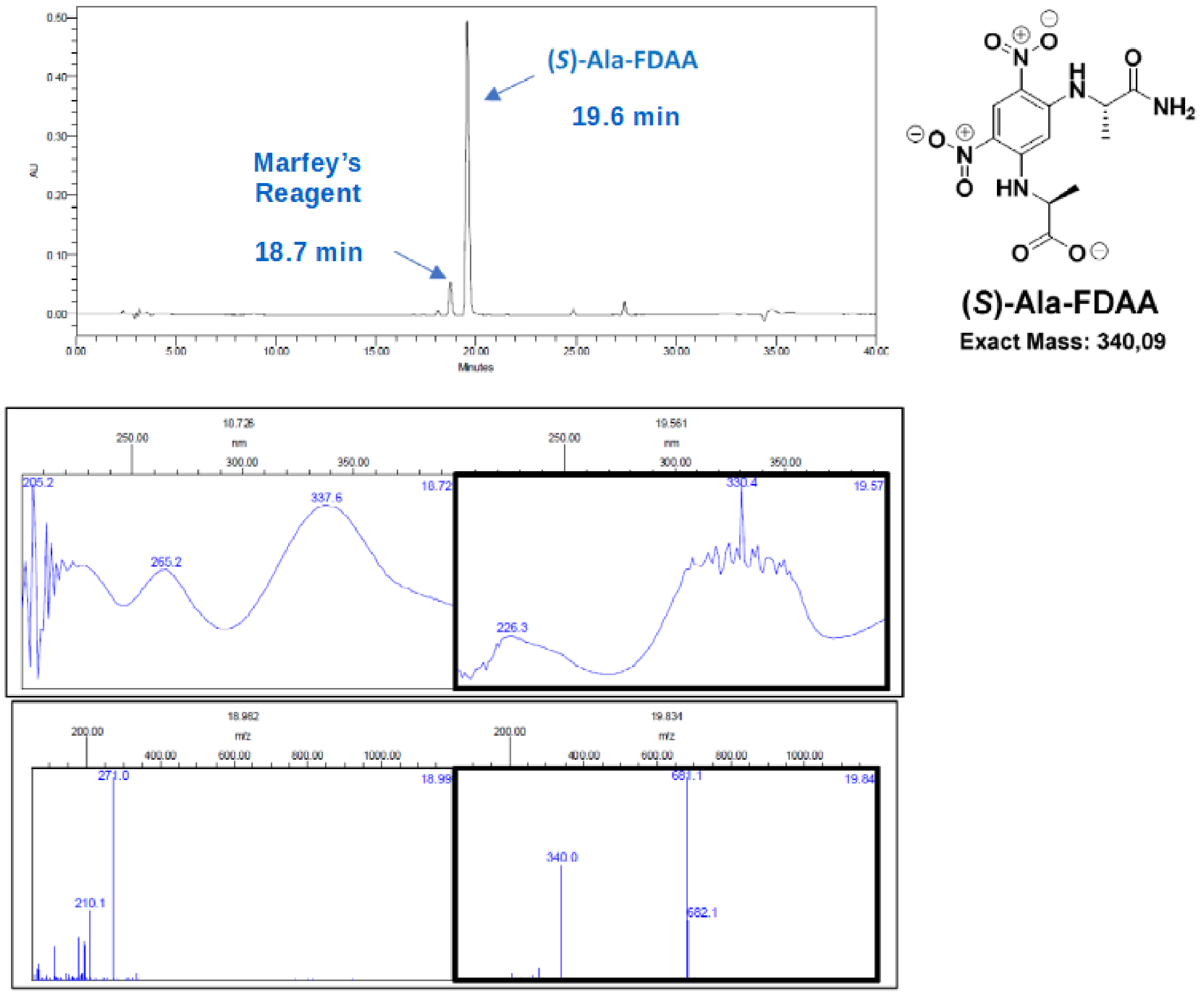

Figure S31. Chromatogram of Marfey reaction with Ala (280 nm), UV and MS spectrum (ESI⁻).

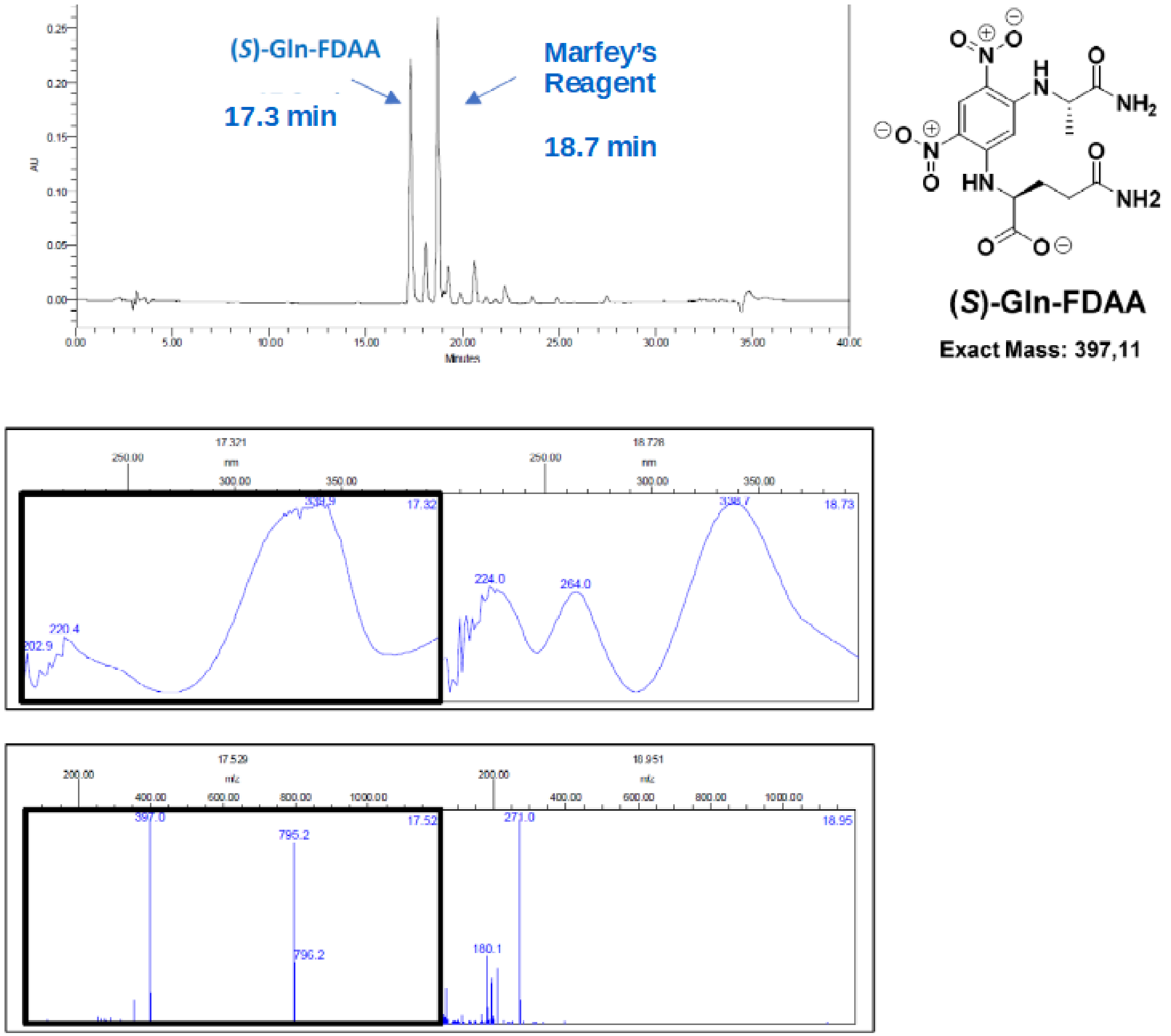

Figure S32. Chromatogram of Marfey reaction with Gln (280 nm), UV and MS spectrum (ESI$^-$).

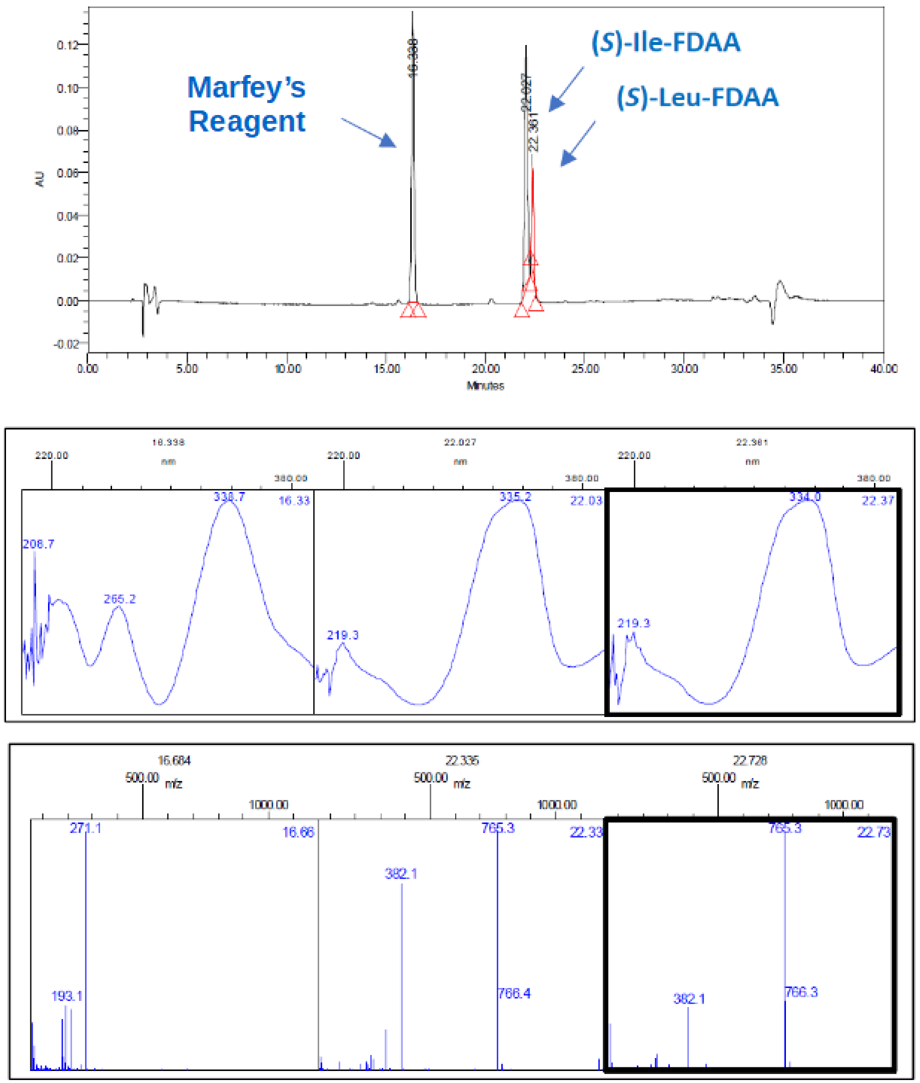

Figure S33. Chromatogram of mixture 1, containing: Leu and Ile, at 280 nm, UV and MS spectrum (ESI$^-$).

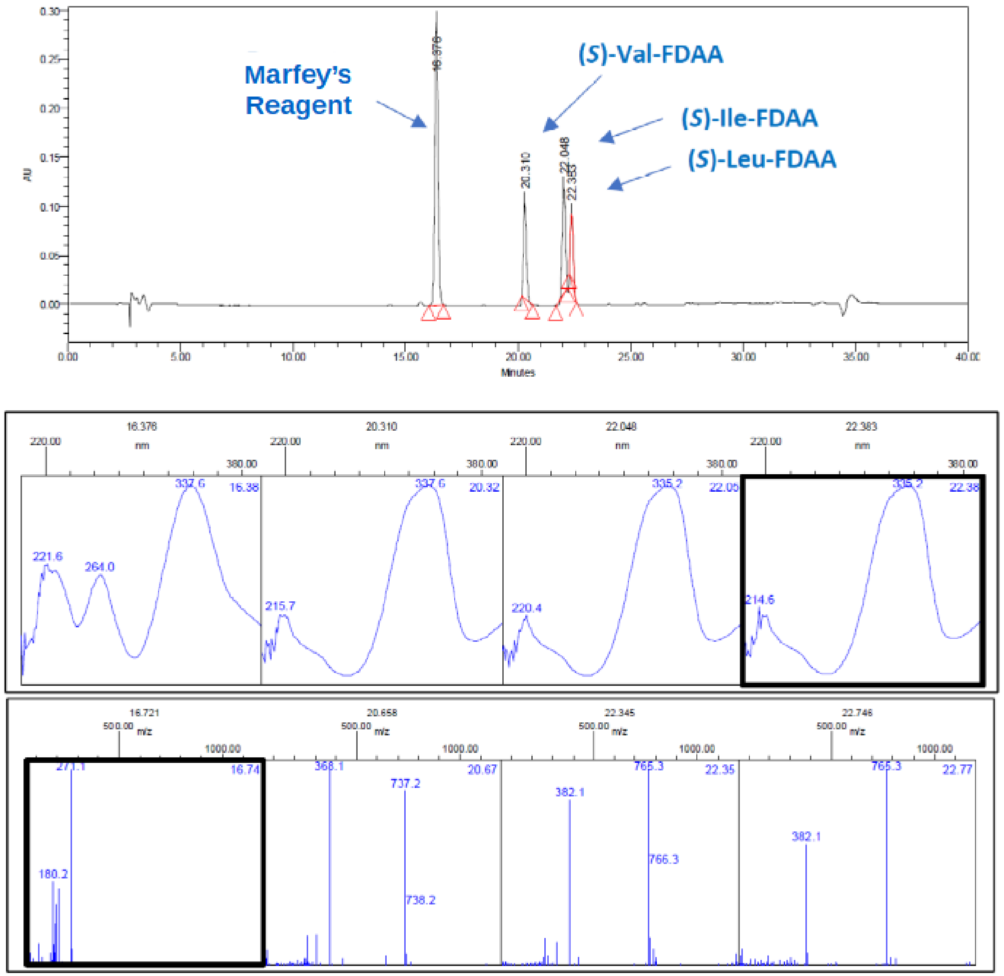

Figure S34. Chromatogram of mixture 2, containing: Val, Leu and Ile, at 280 nm, UV and MS spectrum (ESI$^-$).

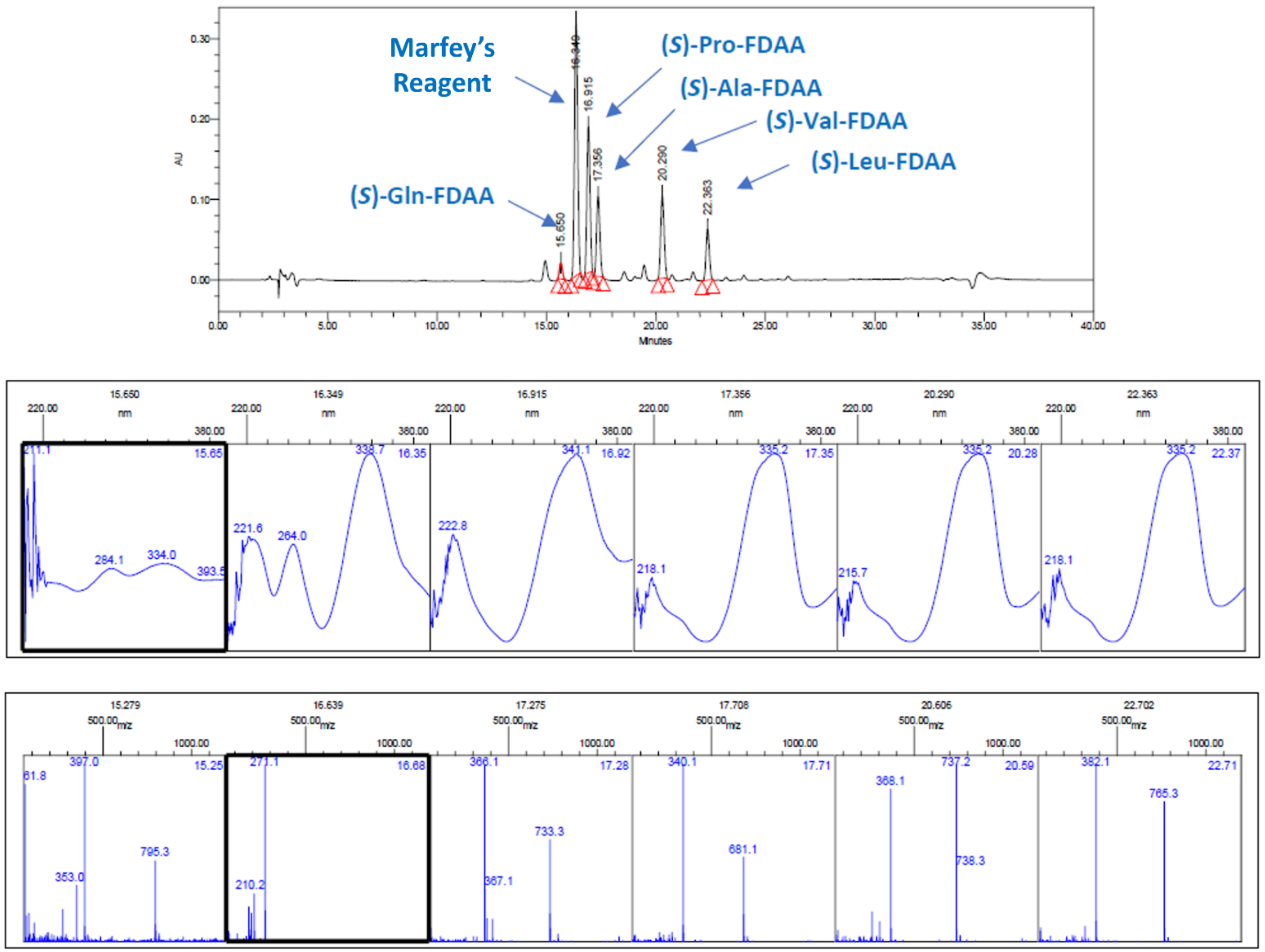

Figure S35. Chromatogram of mixture 3, containing: Val, Leu, Pro, Ala and Gln, at 280 nm, UV and MS spectrum (ESI-).

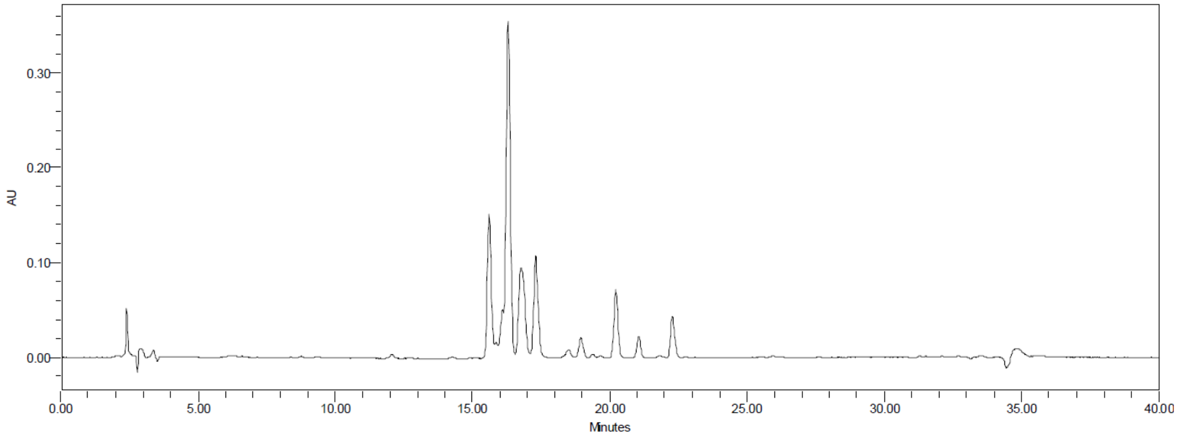

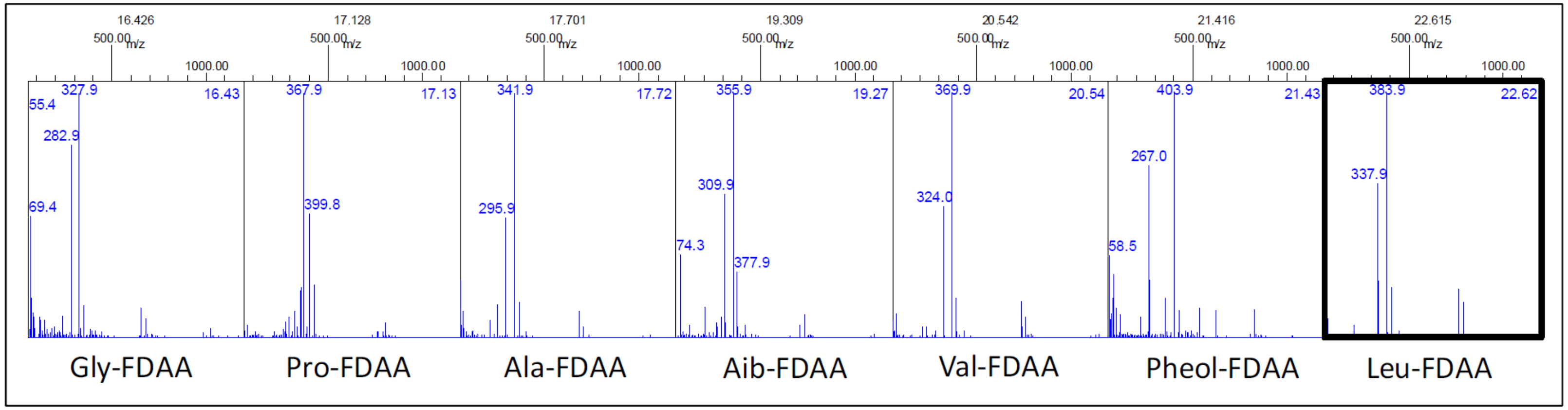

Figure S36. Chromatogram of the Marfey reaction for the hydrolyzed trichokonin VI (**1**) (280 nm), UV and MS spectrum (ESI$^+$).

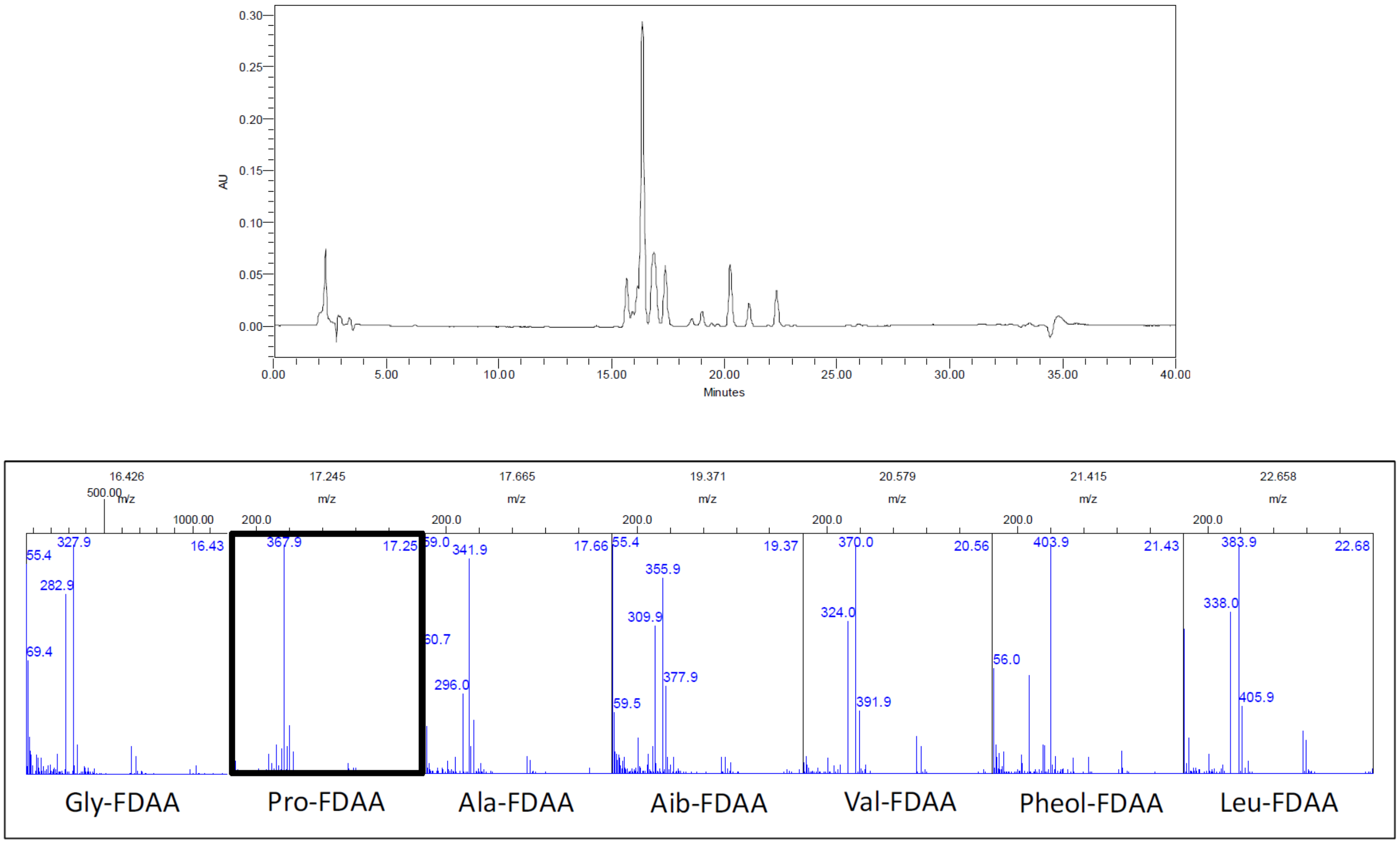

Figure S37. Chromatogram of the Marfey reaction for the hydrolyzed trichokonin VIII (**2**) (280 nm), UV and MS spectrum (ESI$^+$).

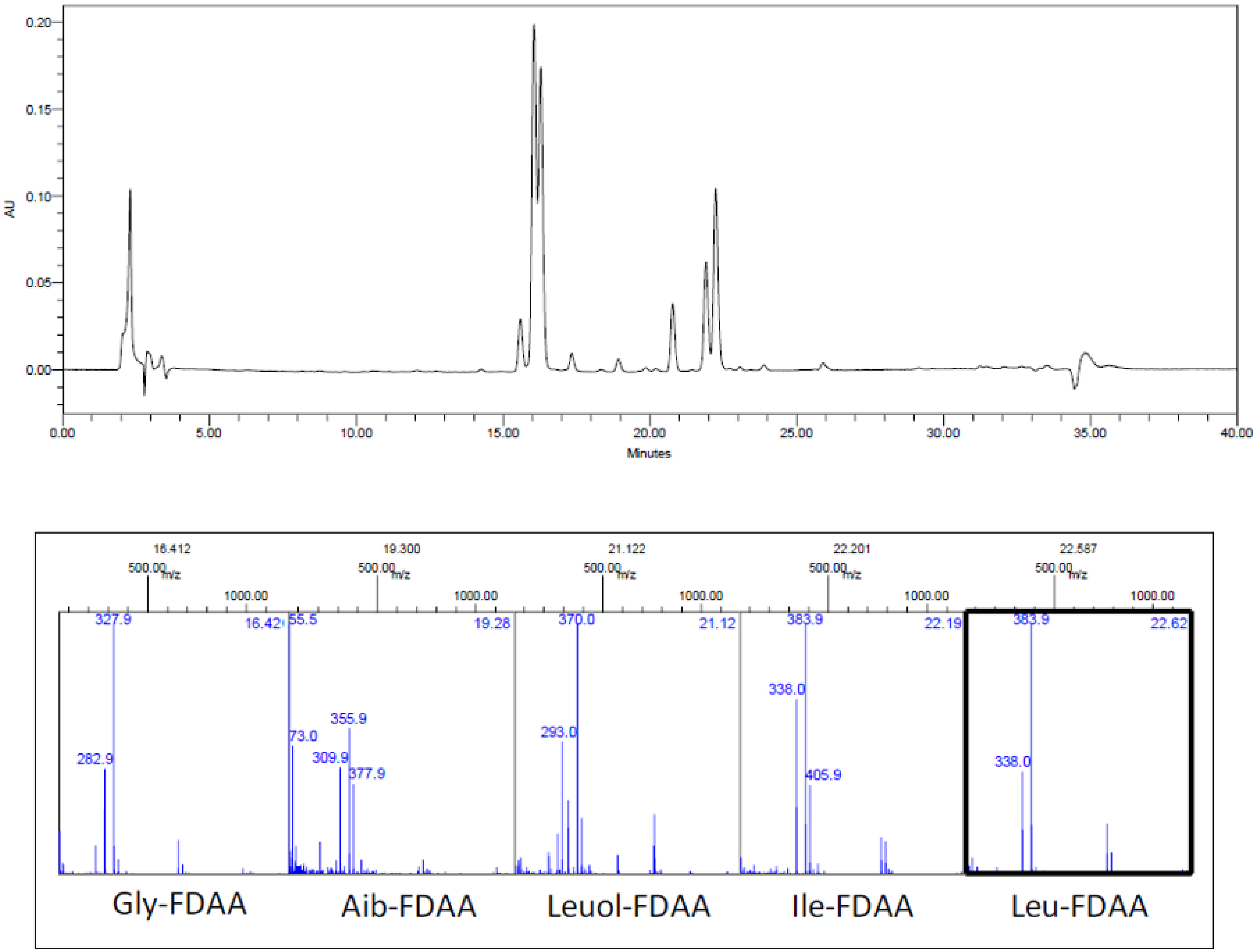

Figure S38. Chromatogram of the Marfey reaction for the hydrolyzed trichogin A IV (**3**) (280 nm), UV and MS spectrum (ESI+).

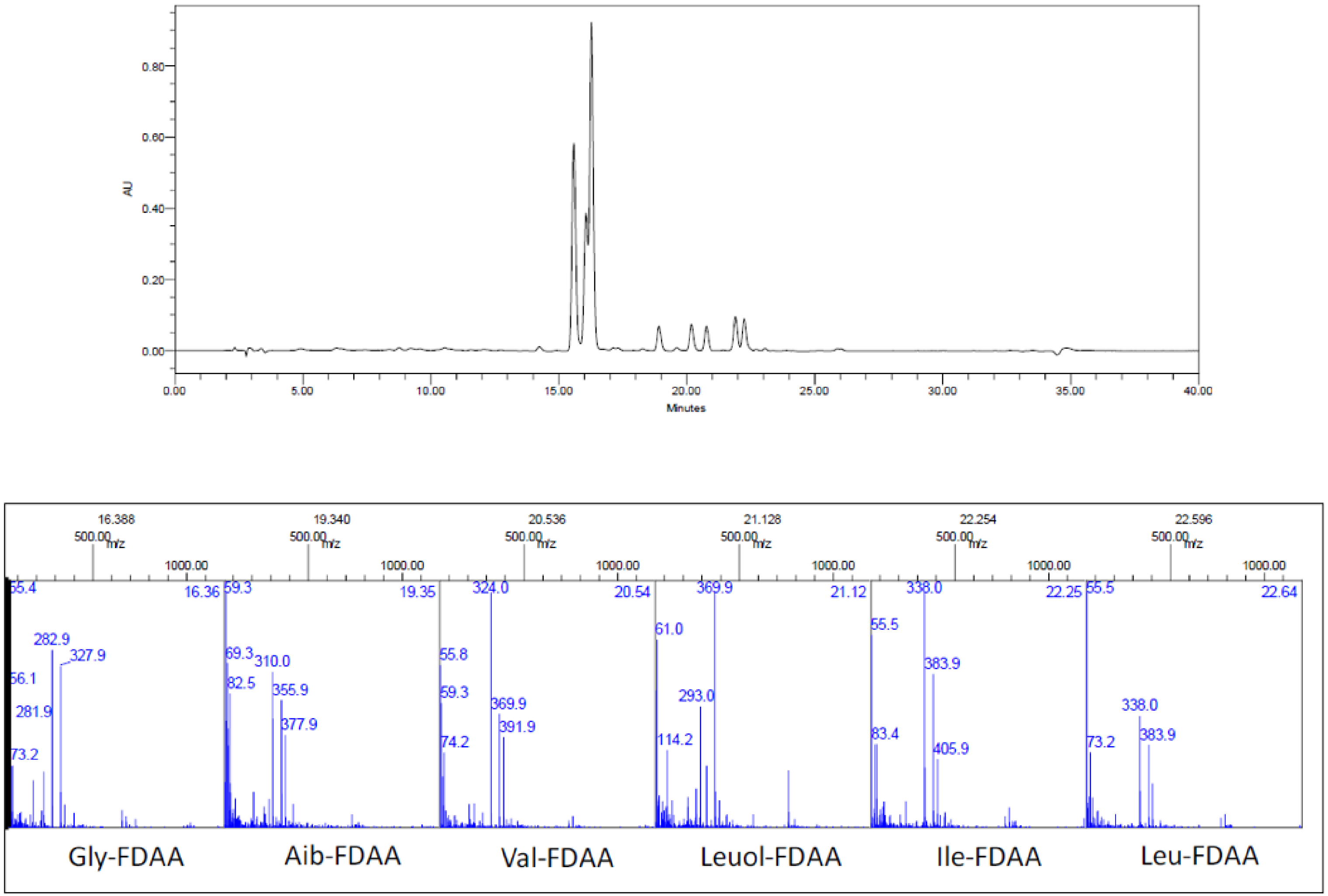

Figure S39. Chromatogram of the Marfey reaction for the hydrolyzed hypocrin NPDG F (**4**) (280 nm), UV and MS spectrum (ESI+).

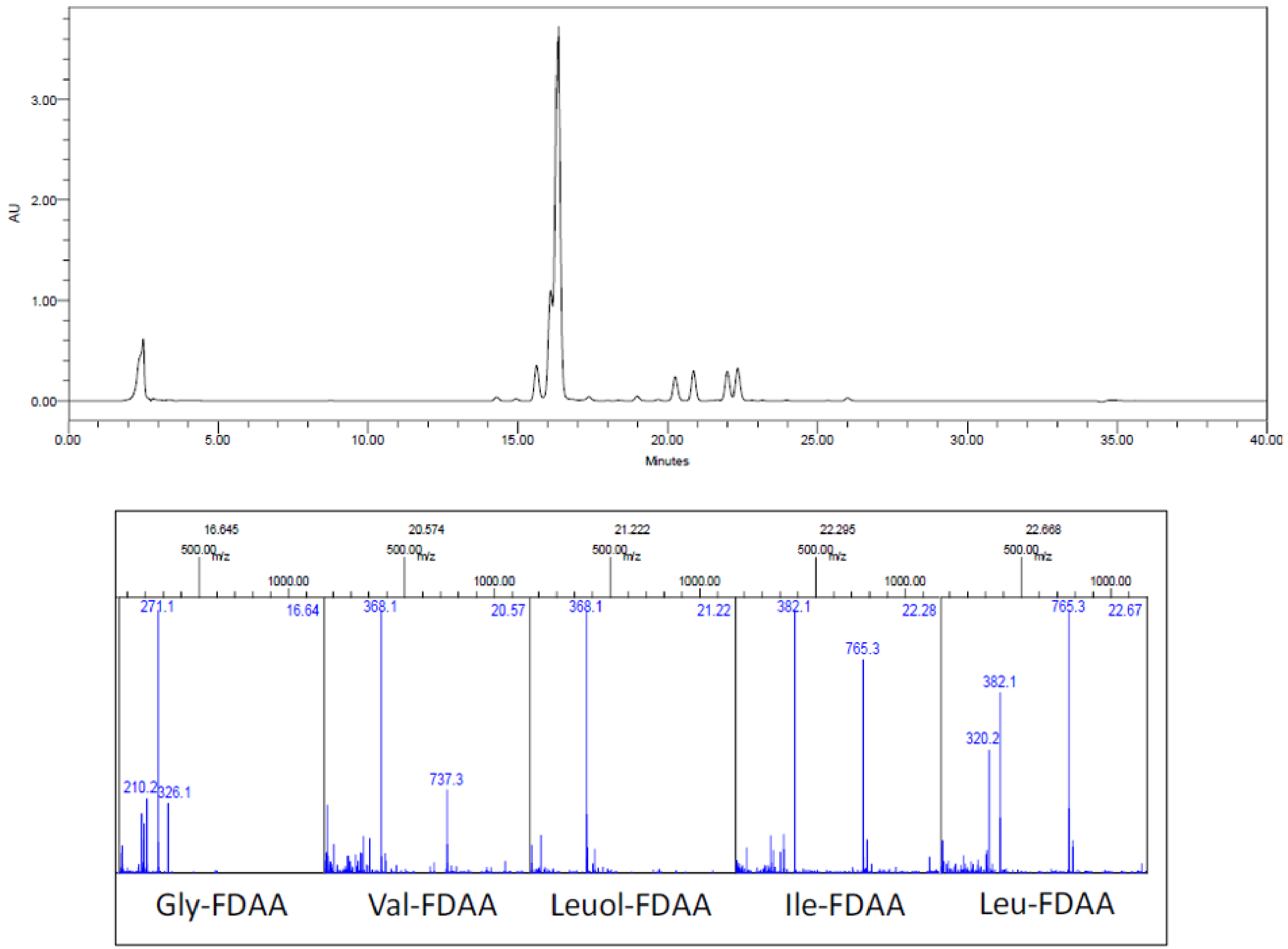

Figure S40. Chromatogram of the Marfey reaction for the hydrolyzed hypocrin NPDG H (**5**) (280 nm), UV and MS spectrum (ESI+).

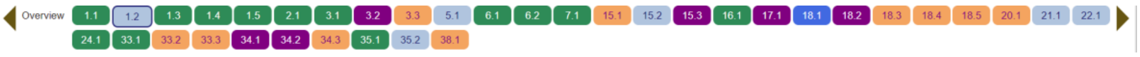

Figure S41. The BGC overview by fungiSMASH. Scaffold 1 and contig 2 (1.2) has 21 modules for peptaibol biosynthesis.

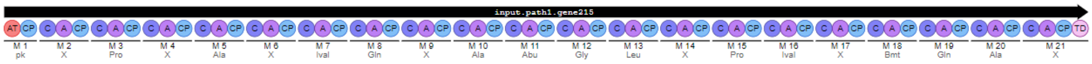

Figure S42. The 21-module PKS-NRPS from the putative peptaibol BGC 1.2 by fungiSMASH. AT: Acyltransferase domain; CP: carrier protein domain; C: Condensation domain; A: Adenylation domain.

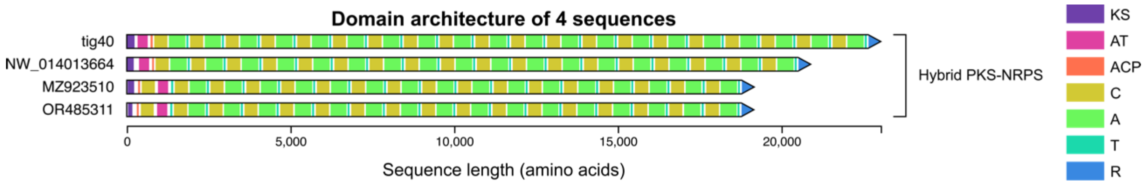

Figure S43. Synthaser analysis of domains contained within peptaibol PKS-NRPS enzymes with different size of peptaibols. KS: ketosynthase domain AT: acyltransferase domain; ACP: acyl carrier protein domain; C: condensation domain; A: adenylation domain; T: thiolation domain; R: terminal reductase domain.

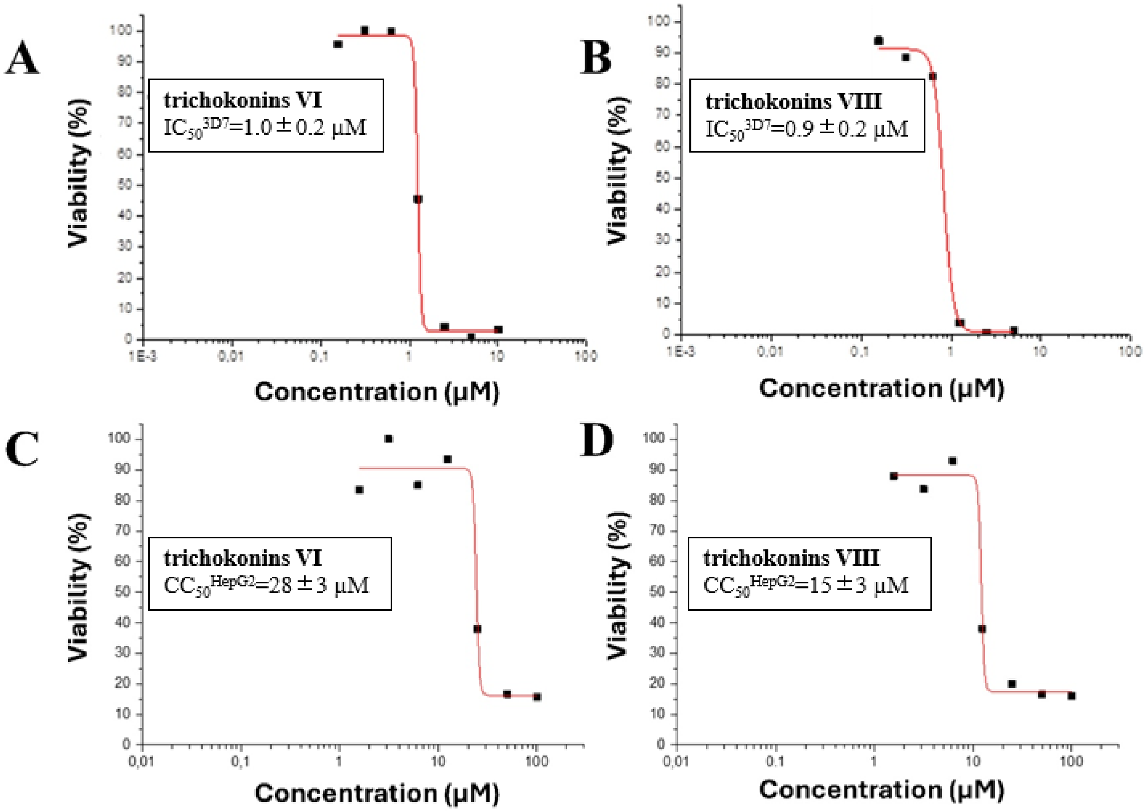

Figure S44. Representative concentration-response curves of (**A**) Trichokonins VI (**1**) and (**B**) VIII (**2**) against *P. falciparum* (3D7 strain, chloroquine-sensitive). Representative concentration-response curves of (**C**) Trichokonins VI (**1**) and (**D**) VIII (**2**) against human hepatocellular carcinoma cells (HepG2 cell line).

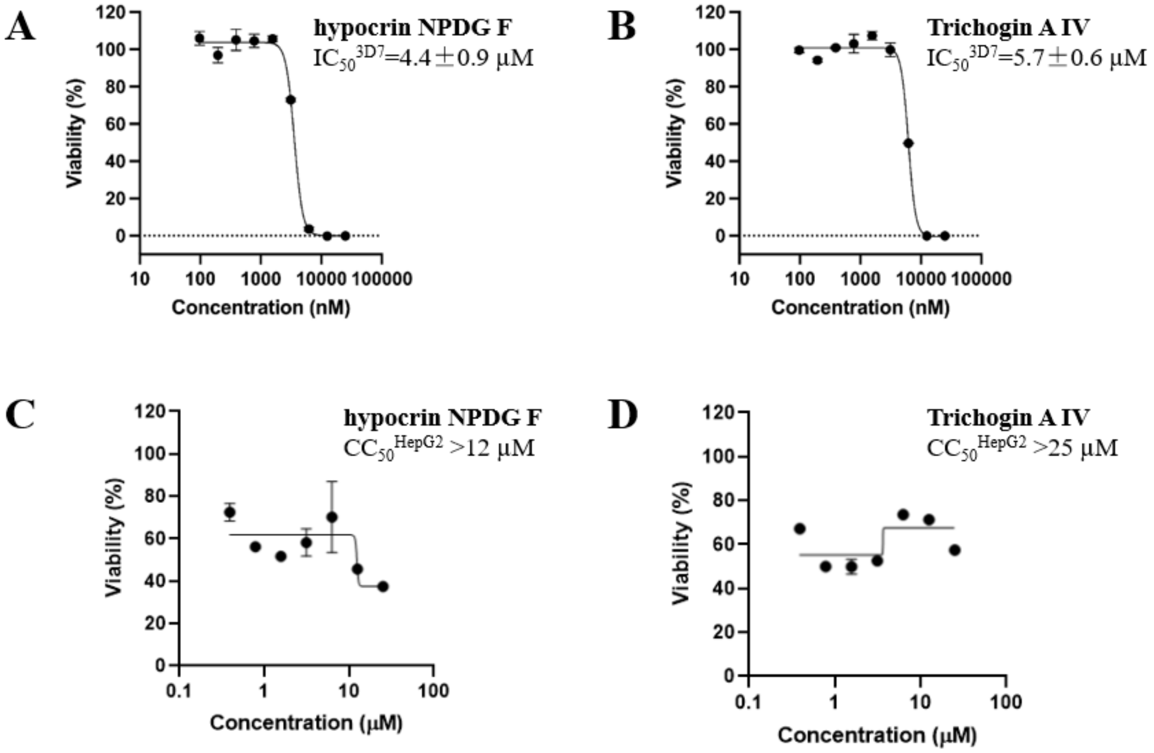

Figure S45. Representative concentration-response curves of (**A**) hypocrin NPDG F (**4**) and (**B**) trichogin A IV (**3**) against *P. falciparum* (3D7 strain, chloroquine-sensitive). Representative concentration-response curves of (**C**) hypocrin NPDG F (**4**) and (**D**) trichogin A IV (**3**) against human hepatocellular carcinoma cells (HepG2 cell line).

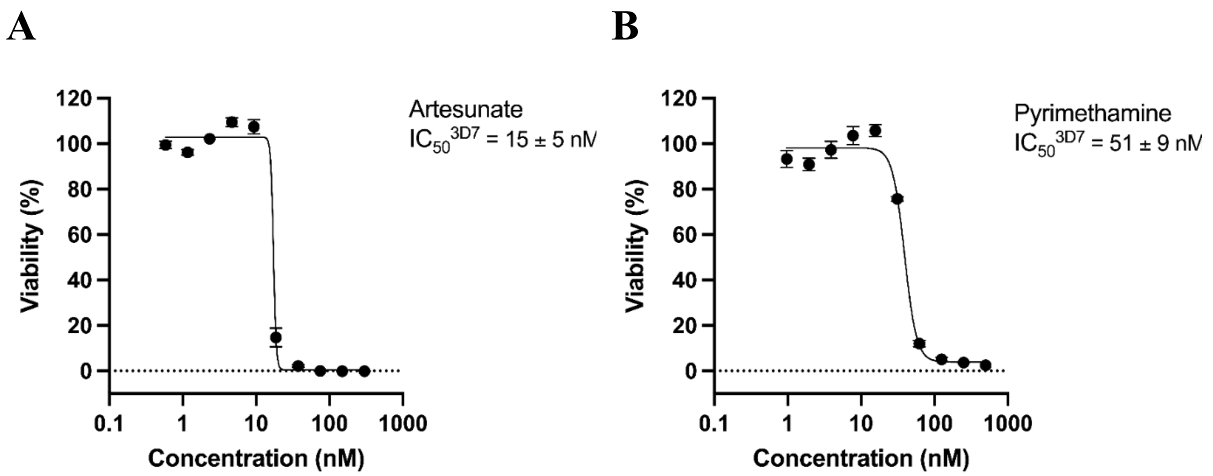

Figure S46. Representative concentration-response curves of positive controls (**A**) artesunate and (**B**) pyrimethamine against *P. falciparum* (3D7 strain, chloroquine-sensitive).
